# Data simulation to optimize frameworks for genome-wide association studies in diverse populations

**DOI:** 10.3389/fgene.2025.1559496

**Published:** 2025-06-18

**Authors:** Jacquiline W. Mugo, Nicola Mulder, Emile R. Chimusa

**Affiliations:** ^1^ Allergology and Clinical Immunology Unit, Department of Medicine, Faculty of Health Sciences, University of Cape Town, Cape Town, South Africa; ^2^ Division of Computational Biology, Department of Biomedical Sciences, Medical School Cape Town, Institute of Infectious Disease and Molecular Medicine, University of Cape Town, Cape Town, South Africa; ^3^ Department of Applied Sciences, Faculty of Health and Life Sciences, Northumbria University, Newcastle Upon Tyne, United Kingdom

**Keywords:** GWAS, whole-genome sequencing, genetic diversity, genetic risk, admixture, admixture mapping, population genetics

## Abstract

Whole-genome or genome-wide association studies (GWAS) have become a fundamental part of modern genetic studies and methods for dissecting the genetic architecture of common traits based on common polymorphisms in random populations. It is hoped that there would be many potential uses of these identified variants, including a better understanding of the pathogenesis of traits, disease risk prediction, discovery of biomarkers, and clinical prediction of drug treatments for populations and global health. Questions have been raised about whether associations that are largely discovered in European ancestry populations are replicable in diverse populations, can inform medical decision-making globally, and how efficiently current GWAS tools perform in populations of high genetic diversity, multi-wave genetic admixture, and low linkage disequilibrium, such as African populations. Here, we discuss some of the challenges in association mapping and leverage genomic data simulation to mimic structured African, European, and multi-way admixed populations to evaluate the replicability of association signals from current state-of-the-art GWAS tools. We use the results to discuss optimized frameworks for the analysis of GWAS data in diverse populations. Finally, we outline the implications, challenges, and opportunities these studies present for populations of non-European descent.

## Highlights

• Variability in the predictive methods and categorization of functionally relevant genetic variants pose specific challenges in GWAS of diverse populations.
• Current GWAS mixed models may not fully control for sub-variant structure between affected and unaffected samples, especially if there is an environmental component to phenotypic associations with ancestry at local variants or locus-specific ancestry due to admixture and inadequate or closely related reference ancestral populations.
• Methodological development is still needed to directly control for local-specific ancestry tracts in variant-level GWAS, which may further improve the power and reduce false positives in mixed-ancestry or multi-ancestry samples.

## Introduction

The frequent occurrence of population differences in phenotypic outcomes, drug, and treatment responses has important consequences for biomedical sciences. This has been shown to be result of variations in host genomes and differing environments (Tishkoff and Williams, 2002; Goetz et al., 2014; Mao et al., 2007; Martin et al., 2017; Campbell and Tishkoff, 2008). For more than two decades, genome-wide association studies (GWAS) have been common approaches in genetic studies to identify molecular variants underlying these variations and have been used successfully for detecting variants in linkage disequilibrium (LD) within causal genes (direct association) or genes associated to causal genes (indirect association) (Martin et al., 2017; Campbell and Tishkoff, 2008; Seldin et al., 2011). These approaches have become a fundamental part of modern genetic studies, and methods for dissecting the genetic architecture of common traits based on common polymorphisms in different populations have been developed (Seldin et al., 2011). They have enhanced our knowledge on the genetic architecture of many complex diseases, such as heritability estimation, the individual’s genetic reliability of disease (polygenic risk scores, PRSs), the genetic correlation between diseases, number of loci, and their effect sizes (Brody et al., 2017; Chimusa et al., 2018; Sirugo and Tishkoff, 2019). So far, many new genetic associations to diseases have been identified (Chimusa et al., 2018; Sirugo and Tishkoff, 2019). Currently, approaches developed to identify the association between genetic variability and human phenotypes have mostly been designed to capture genomes with a long range of LD and haplotypes, such as those found in European descent populations, who have mostly undergone a population bottleneck (Seldin et al., 2011; Chimusa et al., 2018). For example, a review by Sirugo and Tishkoff (2019) of GWAS diversity in the GWAS catalog up to January 2019 revealed that the reported GWAS were dominated by two populations, with Europeans accounting for 52% and Asian populations accounting for 21% (Sirugo and Tishkoff, 2019). Although the contribution by the Asian population was commendable, further analysis of the individuals in the GWAS by ethnicity revealed a persistent gap: 78% were of European ancestry, 10% were of Asian origin, 10% were from Africa, and 1% were of Hispanic origin, while other ethnicities accounted for less than 1% of the individuals (Sirugo and Tishkoff, 2019; Mills and Rahal, 2019; Visscher et al., 2017). These have raised concerns about healthcare disparity when GWAS results are translated into clinical relevance for global health. Mills and Rahal (2019) analyzed 3,639 GWAS and found that 86.03% of discovery, 76.69% of replication, and 83.19% of combined ancestry diversity in GWAS were mostly from individuals of European descent. The finding corroborates those of other studies on Asian descent populations (Kim et al., 2011) (9.92% discovery, 17.97% replication, and 12.37% combined), African American or Afro-Caribbean populations (1.96% discovery, 1.96% replication, and 1.96% combined), Hispanic or Latin American populations (1.30% discovery, 1.33% replication, and 1.30% combined), other or mixed-ancestry individuals (0.48% discovery, 1.77% replication, and 0.87% combined), and African ancestry populations (0.31% discovery, 0.28% replication, and 0.30% combined). These studies suggest the inclusion of diversity in data and recognize the consequences of the lack of diversity. Kim et al. (2018) examined the risk posed by genetic disease across global populations using GWAS and showed that the ancestral risk allele discovered is 5.1% higher and the derived risk allele discovered is 5.40% lower in African populations. Further investigation using different populations showed that non-African groups yielded disease associations that have biased allele frequencies, while the African populations yielded disease associations that are relatively free of bias. Caution must, therefore, be taken when using GWAS results from one population to predict disease risk in another.

Research continues to reveal that current GWAS results from European cohorts cannot be generalized to diverse populations due to confounding environmental factors across populations, differing patterns of LD, differences in allelic architecture, and other contributing factors (Visscher et al., 2017; Shriner et al., 2011a; Shriner et al., 2011b; Shriner, 2017). Significant differences in European and diverse populations have also been observed in the genetic determinants of both common and rare diseases and their effect sizes (Visscher et al., 2017; Shriner et al., 2011a; Shriner et al., 2011b; Shriner, 2017). Nevertheless, GWAS are now slowly being extended to diverse populations. Non-European populations are now included in large disease-analysis studies, and new consortia have been established in countries with diverse populations. The Human Heredity and Health in Africa (H3Africa) consortium has spearheaded GWAS on the African continent (Mulder et al., 2018). The INdian DIabetes COnsortium (INDICO) (INdian DIabetes COnsortium, 2011) and the GenomeAsia 100K Project (Author anonymous, 2019) are other examples of consortia in diverse populations.

On the other hand, it is still being observed that large numbers of modern drugs approved by the Food and Drug Administration and similar organizations have been developed with relevance to European ancestry populations while not addressing the fact that subtle differences in the genetic make-up of other populations, such as Asian, South American, and African populations, can affect drug efficacy or response (Visscher et al., 2017; Hassan et al., 2020). This has been evidenced by the fact that hundreds of thousands of people still die each year because of adverse drug reactions, which may result from multiple factors, including disease determinants, environmental exposure, human microbiome profiles, and genetic factors (Visscher et al., 2017; Hassan et al., 2020). The use of genetic information to inform medical decision-making, however, raises questions as to whether such use could be equitable. Given differences in allelic architecture, differing patterns of LD, and confounding of environmental factors across populations, the richer mixtures of African genetic variants and differing environments are likely to contribute to wider phenotypic and individual microbiome profile variability (Tishkoff and Williams, 2002; Awany et al., 2018). It is, therefore, crucial to advance GWAS research and assess how well current approaches can capture diverse global population cohorts (Campbell and Tishkoff, 2008; Chimusa et al., 2018; Visscher et al., 2017).

We hypothesize that understanding and then appropriately modeling different aspects of genetic architecture in the African population has the potential to achieve unbiased and powerful estimates of genetic risk in them, as well as in multi-ethnic and admixed populations such as African Americans. Here, we leverage genomic data simulation that mimics African, European, and multi-way admixed populations to evaluate the replicability of association signals from current state-of-the-art GWAS tools. We dissect reasons from the biological and methodological perspectives that account for the replicability of GWAS and identify the challenges ahead. In contrast to the exemplary success of disease gene discovery, currently, GWAS findings are not fully useful for predicting phenotypes. Finally, we provide an overview of the prospects for individual prediction of disease risk and its foreseeable impact on clinical practice in populations of non-European descent.

## Genome disease mapping

The delineation of health and complex diseases from polymorphism-based association mapping holds promise to bridge the gap between clinical translation and statistical association, thereby improving diagnostics, screening, genetic testing, and counseling in global clinical populations (Sirugo and Tishkoff, 2019; Mills and Rahal, 2019; Martin et al., 2019). It has been shown that variants associated with diseases found in populations of European ancestry do not always replicate in diverse populations such as African populations (Chimusa et al., 2018; Kim et al., 2018; Martin et al., 2019) for several reasons, including confounding of environmental factors across populations, differing patterns of LD, and differences in the allelic architecture.

Although efforts such as the China Kadoorie Biobank (Chen et al., 2011), Global Screening Array (GSA) (Nelson et al., 2017; Kalra et al., 2018), Multi-Ethnic Global Array (MEGA) (Nelson et al., 2017), and H3Africa array (Mulder et al., 2018) have recently enabled effective genome-wide DNA microarrays in diverse populations, there are still many issues in GWAS, such as (1) GWAS small sample size in diverse populations, including in African populations (Swart et al., 2022); (2) stratification due to the correlation of environmental exposures and genetic correlation background due to common ancestry or multi-wave admixture and pre-/post-admixture selection pressure (Chimusa et al., 2018); (3) translation of associated loci into suitable biological hypotheses (Chimusa et al., 2018); (4) the understanding of how multiple modestly associated loci within genes interact to influence a phenotype (Chimusa et al., 2018). Control of population stratification in GWAS has been one of the biggest concerns to ensure that observed associations reflect the genetic effects of each genomic locus rather than correlations with ancestry (Author anonymous, 2019; Korte and Farlow, 2013).

**FIGURE 1:**
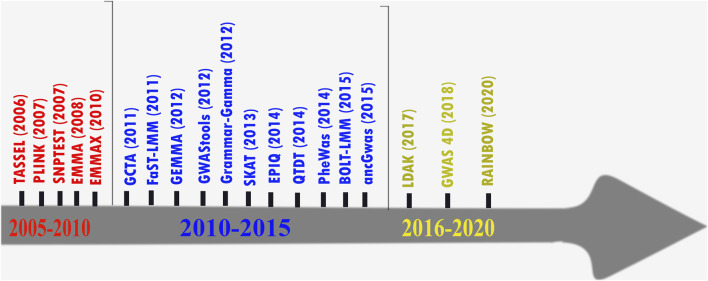
Examples illustrating the chronological order of a partial list of GWAS tools.

For decades, mixed-model approaches have been attractive in GWAS as they allow the inclusion of all samples irrespective of ancestry. Mixed-model approaches control for population stratification by modeling distant relatedness between samples due to ancestry (Korte et al., 2012). Several implementations exist, and we list some of them chronologically up to the latest in 2020 in Figure 1. Mixed models may yield greater statistical power, both through increased sample size and by controlling for the variance explained by the genetic relatedness between individuals (i.e., a random-effect component) (Zhou and Stephens, 2012; Chimusa et al., 2014). However, there is evidence that variants with low frequency (1%–5%) or at the boundary may not often attain genome-wide significance in mixed models due to their imperfect asymptotic distribution (Chimusa et al., 2014). In addition, they may not fully control for sub-variant structure between affected and unaffected samples, especially if there is an environmental component to phenotypic associations with ancestry at local variants or locus-specific ancestry due to admixture (Seldin et al., 2011; Winkler et al., 2010; Brody et al., 2017; Shriner et al., 2011b).

Non-genetic factors such as environmental exposures may be correlated with genetic ancestry due to the shared local environment (familiar or community effects) or the relationship between ancestry and sociocultural factors such as ethnicity and religious background (McGrath et al., 2013). Effective methodological development is still needed to directly control local-specific ancestry tracts in variant-level GWAS, which may further improve power and reduce false positives in mixed-ancestry or multi-ancestry samples (Visscher et al., 2017; McGrath et al., 2013; Marigorta et al., 2018). However, it is worth noting that several efforts and advances have been made in leveraging the complementarity of single-nucleotide polymorphism (SNP) association signals captured through GWAS and admixture signals to calibrate and improve GWAS power in admixed populations (Shriner et al., 2011a; Shriner et al., 2011b; Shriner, 2017; Chimusa et al., 2014). In doing so, numerous studies have leveraged local-specific ancestry tracts in variant-level association analyses to African Americans (Peprah et al., 2015), Latinos (Gonzalez et al., 2016), South African Coloureds (Chimusa et al., 2014), and Hispanic cohorts (Chen et al., 2015), demonstrating added value beyond standard association testing (Geza et al., 2019; Chimusa et al., 2016). Admixture association is critically reliant on accurate locus ancestry inference (LAI), which requires well-specified founding population reference samples (Chakraborty and Weiss, 1988). Power can be optimized by combining admixture mapping and association testing (Kim et al., 2018), but this approach is rarely adopted because of the multi-stage process required and the challenge in application to complex multi-way admixed samples (Thornton and Bermejo, 2014).

For example, currently available joint ancestry and SNP association methods (Mugo et al., 2023; Atkinson et al., 2020; Atkinson et al., 2021) have primarily been tailored to African American populations (two-way admixed populations), despite the authors’ suggestion that, in theory, these methods could be extended to more than three-way admixed populations. This has either been left for future research or not optimized for multi-way admixed populations in most of these tools. In addition, the accuracy of these approaches relies on the use of accurate knowledge of ancestry inference, particularly when applying to complex multi-way admixed populations (Mugo et al., 2023). Although recently introduced locus-specific ancestry methods for multi-way admixed populations achieved equivalent accuracy (Geza et al., 2019; Honorato-Mauer et al., 2025; Sun et al., 2025), they still suffer from spurious deviations in average local ancestry at some chromosomal locations of cases and controls, where the modeled ancestral population is unusually different from the true ancestral population due to historical actions such as natural selection and also inaccurate or closely related reference ancestral populations (Chimusa et al., 2014; Geza et al., 2019; Sun et al., 2025). Furthermore, application of these methodologies to multi-way admixed populations, like most African populations known to have high diversity within differing environments, remains mostly less explored (Author anonymous, 2019). There remains a critical need to (1) improve LAI accuracy (Chimusa et al., 2014; Geza et al., 2019; Thornton and Bermejo, 2014; Mugo et al., 2023; Atkinson et al., 2020); (2) develop methods for optimizing the power of association testing (Chimusa et al., 2014; Geza et al., 2019; Mugo et al., 2023; Atkinson et al., 2020; Atkinson et al., 2021) and PRSs in admixed data (Sirugo and Tishkoff, 2019; Coram et al., 2017; Marnetto et al., 2020); (3) build integrative software for running a multi-stage admixture analysis pipeline (Geza et al., 2019).

## Replication in genetic association studies

In general, the replication of reported results is the most reliable validation of scientific discoveries (National Academies of Sciences et al., 2019; Kraft et al., 2009). In complex trait genetics, replication occurs when the same genetic marker is consistently associated with the same phenotype in independent cohorts. In addition, a positive association at variants in strong LD with the original marker SNP is often used as evidence of replication, even if formal exact replication is not achieved. Cross-ancestry replicability has the power to shed light on the genetic architecture of complex traits, informing the reliability of effect estimations and their variability across human ancestries.

GWAS meta-analysis has increasingly that leverages association summary statistics to facilitate and encourage *silico* replication to maintain reliability in genetic association findings has increasingly been adopted. A meta-analysis framework combines results from different GWAS cohorts into a single analysis framework to recover signals that a single GWAS cohort study might miss and to address the between-study and between-population heterogeneity (Chimusa et al., 2016). Recently, meta-analysis has shown remarkable discovery results and helped us better understand and validate association results from different studies. Meta-analysis is considered a post-genome-wide association study method; however, heterogeneity among GWAS meta-analyses remains an issue, particularly with increasing number of studies (Kraft et al., 2009). Variation in the cohort size across independent studies is challenging, especially when these studies are conducted from distinct populations of different ancestry and patterns of LD (Kraft et al., 2009). Similarly, the list of new post-GWAS tools, such as multi-marker analyses, which go beyond single-SNP tests, or the inclusion of functional evidence to reweight GWAS results, is growing by the day (Han and Eskin, 2011). The heterogeneity in these methodologies will necessarily complicate the evaluation of replicability.

Another caveat is that GWAS conducted in non-European ancestry populations usually include fewer samples (Mieth et al., 2016), making the current picture of genetic association to disease across diverse populations incomplete (Manolio, 2017). This creates a challenge for the power of GWAS meta-analysis across diverse population cohorts of differing genetic ancestry. Moreover, caution is required as incomplete replication can also be informative; several studies have reported a lack of interpopulation replicability, indicating that some risk variants are population-specific (Martin et al., 2017; Nakagome et al., 2012). For example, comparing Asian and European associations with major depression, the failure to replicate is largely due to differences in patterns of LD, which reduced power in one population since the proportion of attributable risk decreases with population-specific minor allele frequency (Wang et al., 2007).

## GWAS application for disease risk prediction

The exceptional polygenicity of human traits makes unraveling mechanisms from whole-genome or GWAS daunting (Mills and Rahal, 2019). PRSs, which estimate an individual’s genetic liability to disease or traits compared to others with a different genetic constitution (Wray et al., 2021; Choi et al., 2020), are still mostly derived from European ancestry GWAS data, making their predictive power substantially lower when computed in non-European samples, particularly those of African ancestry (Sirugo and Tishkoff, 2019). Furthermore, the development of disease association studies and PRS methods (Choi et al., 2020; Euesden et al., 2015; Choi and O'Reilly, 2019; Vilhjálmsson et al., 2015), their applications to understand disease etiology, and their evaluation for clinical utility have been explored almost entirely in European ancestry populations (Sirugo and Tishkoff, 2019).

PRS portability and generalizability have been widely reported in recent years (Martin et al., 2017), yet PRSs in non-European ancestry samples are still routinely calculated using the same European GWAS data and PRS methods as applied to Europeans. This takes no account of known population genetic factors affecting the data, such as marked LD differences, genetic drift, natural selection, daily nutrition, family history, and gene–environment interactions. Consequently, the clinical utility and etiological insights provided by PRS may have little relevance to Africans and African Americans. In addition, PRS calculations are inherently dependent on the quality of the underlying GWAS data. If the GWAS used to derive the PRS model is underpowered or has biases, the PRS may not be accurate or reliable and makes its application to a multi-way admixed population even worse. Furthermore, current PRS methods are limited in their ability to integrate epigenetic factors and interactions between different genetic regions. Although PRS can summarize the effects of individual genetic variants, they do not yet account for how gene expression may be regulated by epigenetic modifications or how different genetic variants may interact to influence disease risk. While it is worth noting the advances currently being made in more sophisticated PRS models (Wray et al., 2021; Klau et al., 2023), there is an increasing risk of overfitting, particularly when the models incorporate many genetic variants. Overfitting occurs when a model is too closely aligned with the training data, resulting in a model that does not generalize well to new data sets (Wray et al., 2021; Klau et al., 2023). There is still a need to ensure that PRS models maintain robustness and a similar magnitude of accuracy in different populations through careful validation and cross-validation strategies, as well as by integrating an explainable predictive model within PRS (Klau et al., 2023).

All these raise the question as to how the clinical utility of these methods can be made equitable across multi-ethnic populations and, specifically, how to accurately predict health and disease risk in multiway admixed and African populations.

## Leveraging the data simulation framework to dissect GWAS in diverse populations

Simulation of homogeneous and mixed-ancestry case/control populations with well-known structures that mimic real populations may help better understand the genetic variation of these populations and evaluate different existing GWAS tools of genetic variations undermining complex diseases.

The genetic structure of populations, as well as other controllable factors, including allele frequency and LD patterns of genetic markers, is important for the simulation of genotyping data for GWAS (Mugo et al., 2017). It is important to note that the power of a statistical test to detect a risk locus relies heavily on the allelic spectrum (numbers and frequencies of alleles) and the LD structure around the locus (Mugo et al., 2017). Therefore, it has been suggested that simulated data should possess both local and long-range LD (LRLD) patterns and maintain allelic frequencies like real data (Turgut and Koca, 2024). The resampling approach starts with real data and avoids the use of an evolutionary process. It has been shown that this method has its advantages, compared to other approaches, in retaining real data properties, such as allele frequency and LD of the initial pool data (Mugo et al., 2017).

### Simulation of multiple disease loci

To facilitate benchmarking common GWAS tools, we simulated homogeneous and heterogeneous GWAS datasets based on haplotypes from the 1000 Genomes Project spanning the genome and realistic enough to mimic African, European, and admixed populations to challenge the statistical methods for association testing under real-world conditions.

We used resampling and population growth models with recombination breakpoints while mimicking mutation rates as described in our previous simulation tool FractalSIM (Mugo et al., 2017). African and European populations were simulated under a homogeneous simulation model. We merged five European and two West African populations to form the reference population for the simulation. Merged populations, the corresponding sample sizes, and the abbreviations for the populations used are listed in Supplementary Table S1.

We selected 9,139,969 common biallelic SNPs in both European and African populations. Two sets of case/control datasets with an equal number of cases and controls (500 cases, 500 controls and 2,500 cases, 2,500 controls) were simulated for each merged population, while maintaining population substructure (Mugo et al., 2017), in generating simulated genome datasets and mimicking each reference panel. Although it is well known that GWAS power is correlated with increasing samples, these sample sizes were modestly chosen to (1) reflect current African GWAS sample size affordability (Fatumo and Inouye, 2023; Olono et al., 2024) and (2) allow a realistic evaluation of the association power on the most popular and commonly used GWAS tools through comparing GWAS summary statistics in European versus African populations. Our decision here relies on the fact that most GWAS in non-European populations still suffer from small sample sizes, and we, therefore, base our evaluation on the minimum and maximum sample sizes that African studies can currently afford, as indicated in the current literature (Mills and Rahal, 2019; Zhou and Stephens, 2012; Turgut and Koca, 2024; Fatumo and Inouye, 2023; Olono et al., 2024).

**FIGURE 2:**
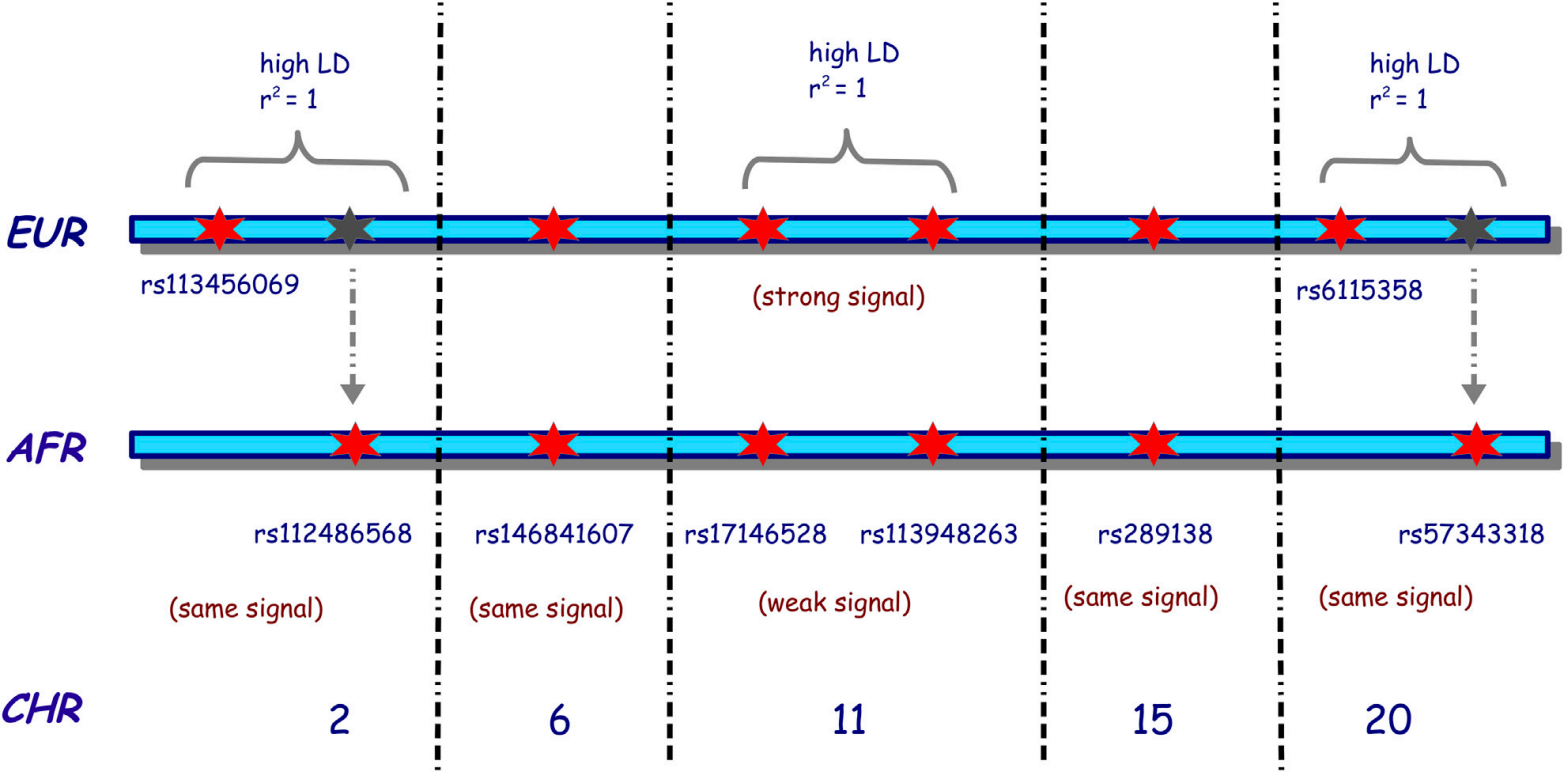
Illustration of the choice of the risk SNPs selected for the simulation of homogeneous European and African populations. EUR indicates European, AFR indicates African, and CHR indicates the chromosome. A red star indicates that the SNP was simulated as a risk SNP in that population. In contrast, a gray star indicates that an SNP is present in the European population, in high LD with the risk SNP simulated on that chromosome but simulated as a risk SNP in the African population. The black dotted vertical lines indicate the presence of other chromosomes between the two chromosomes. The risk SNPs on chromosomes 2, 6, 15, and 20 were simulated with the same risk strength in both populations, while on chromosome 11, the risk SNPs of Europeans were simulated with a strong risk signal, and those of Africans were simulated with a weak risk signal.

To assess the associated risk effect, a total of eight fixed SNPs were simulated with risk effects through all simulation scenarios. These SNPs were randomly selected across the genome, and as such, we simulated the risk effect on SNPs on chromosomes 2, 6, 11, 15, and 20. Figure 2 illustrates our choice of the tag SNPs (representative SNPs that represent a group of SNPs in a genomic region) and risk SNPs across different chromosomes. On chromosome 2, we chose two SNPs, rs113456069 and rs112486568, which were selected, such that they were in complete LD (*r* ^2^ = 1) in the simulated European dataset. SNP rs113456069 was then simulated with a risk effect in the simulated European population, while rs112486568 was simulated with a risk effect in the simulated African population and no effect in the simulated European population. Both SNPs were simulated with the same signal strength in both simulated datasets. A similar process was applied in simulating SNPs with risk effects on chromosome 20. SNPs rs6115358 and rs7343318 were in complete LD in the European reference dataset, but only rs6115358 was simulated with a risk effect in the simulated European dataset, and rs57343318 was simulated with a risk effect in the African populations. The objective of this design in simulating the risk-effect SNPs on chromosomes 2 and 20 was to enable investigating GWAS replicability from simulated European into simulated African GWAS datasets and evaluate the rate of misassociation and misreplication. The SNPs with risk effects on chromosome 11 were simulated such that they were in complete LD in both simulated datasets, but in the European dataset, they were simulated to have a strong signal, while in the simulated African dataset, they were simulated to have a weak signal. This was to enable examining the power from different tools for capturing weak association signals in simulated African population and understanding if increasing samples may have contributions. On chromosomes 6 and 15, both SNPs were simulated with the same risk signal strength in both simulated datasets. In addition, we considered the same homozygosity and heterozygosity relative risks for these eight risk tag SNPs in all simulation scenarios. The list of these SNPs and the corresponding relative risks in the simulated European and African populations are listed in Supplementary Table S2. The cases and controls were then simulated using a multiple logistic regression model implemented in FractalSIM (Mugo et al., 2017).

The heterogeneous (admixed) datasets were generated under a single-point admixture scenario, where the admixture process occurs at a single point in history, such that the current generation is the offspring of the admixed population that has interbred over subsequent generations. Considering a random mating model where interbreeding has occurred for 10 generations, the admixture simulation first mimicked the isolated growth of each parental (reference) population, where a disease model (risk or null) was simulated in the isolated homogeneous simulation for each of the parental populations, like the case–control homogeneous simulation of the European and African datasets detailed above. At generation 0, the isolated parental populations were allowed to interbreed. We simulated both three-way and five-way admixture scenarios. Supplementary Table S3 lists the reference parental populations used in these two scenarios, their corresponding initial sample sizes, and the proportion of ancestry contribution from each of these parental/reference populations.

In the three-way simulation, we included 466,142 biallelic SNPs that were the intersection between the three parental populations. We simulated eight risk SNPs as described above and generated 2,500 cases and 2,500 controls. In the five-way admixture scenario, we incorporated 623,330 biallelic SNPs that were present in the intersection between all five parental populations and simulated eight risk SNPs on chromosomes 2, 6, 11, 15, and 20 as described above. In the five-way scenario, however, we simulated two sets of datasets of different sample sizes: a dataset of 500 cases and 500 controls and another of 2,500 cases and 2,500 controls.

We simulated different ancestry risk scenarios on different chromosomes by varying the presence and strength of genotype risk on the risk variant simulated and the ancestry risk on the genomic region containing the variant. We simulated ancestry risk by simulating ancestry deviation between cases and controls in the region that contained risk variants. In the three-way simulation on chromosomes 2 and 11, we simulated strong genotype and ancestry risks; on chromosome 6, we simulated very strong ancestry risk and weak genotype risk; and on chromosome 15, we simulated weak genotype and ancestry risks. All the other chromosomes were simulated under a null model in this scenario. In the five-way simulation, we simulated similar levels of risk in the 500 cases and 500 controls, as well as 2,500 cases and 2,500 control sample sizes. On chromosome 2, we simulated strong genotype and ancestry risks; on chromosomes 6 and 20, we simulated a strong genotype and no ancestry risk; on chromosomes 11 and 15, we simulated weak genotype and ancestry risks, along with a null model on all the other chromosomes. The risk SNPs simulated in the three-way and five-way scenarios, and their respective homozygosity and heterozygosity relative risks specified in cases are listed in Supplementary Table S4. Depending on the MAF of the risk SNPs, the specified risk effects introduced risk signal strength, as indicated in Supplementary Table S5.

### Assessing simulated GWAS datasets through population structure

We first assessed the structure of the simulated data for both the homogeneous and admixed populations. Since the simulation process was similar for the two sets of case–control datasets in the homogeneous populations and the five-way admixture simulation, we used the 500 cases and 500 controls for this assessment.

In the three simulated datasets, European, African, and admixed, we first merged the simulated GWAS datasets with their corresponding reference populations used in generating simulated data. We then obtained the first 10 principal components (PCs) using principal component analysis (PCA) implemented in genome-wide complex trait analysis (GCTA) and proceeded to plot the first and second PCs using the GENESIS tool (Buchmann and Hazelhurst, 2014). We used two approaches to assess the global ancestry in the admixture simulation. We first ran the ADMIXTURE tool (Alexander et al., 2009), using the supervised option, for the merged admixed datasets; second, we calculated the simulated global ancestry from the local ancestry block estimates generated through FractalSIM. We then plotted the two admixture tract plots for each scenario using the GENESIS tool.

Supplementary Figures S2, S3 show PCA plots for the African and European population simulations, respectively, while Supplementary Figures S4, S6 show the PCA plots for the simulated three-way and five-way admixed populations, respectively. The admixture tract plots for the three-way and five-way admixture simulations are illustrated in Supplementary Figures S5, S7, respectively. From Supplementary Figures S2, S3, we observed that from the simulated African and European populations, both cases and controls, clustered together, as would be expected in a homogenous population, with no population sub-structures. The simulated populations were also positioned between merged reference populations on the PCA 2 axis for the real African population and the PCA 1 axis for the real European population. On the PCA 1 axis, the simulated African population was very close to the reference population by considering the range of the axis, and similarly, on the PCA 2 axis, the simulated European population was also very close to the reference population, based on the range of the axis. This implies that the simulated cases/controls were genetically close to the respective merged African and European reference samples. For the admixed population, we observed on the PCA plots in Supplementary Figures S4, S6 that the admixed samples were confined within their respective reference parental populations, for both the three-way and five-way populations. Furthermore, we observed that the simulated population was spread out, as would be expected for an admixed population. The simulated three-way admixed population was closer to the YRI population, which contributed 70% of the ancestry, while the five-way admixed population is spread out further away from the EAS population but closer to the MAFR and SAS populations, which contributed larger proportions of the ancestry. For the admixture tract, for both the three-way and five-way scenarios, Supplementary Figures S5, S7 demonstrate that the ADMIXTURE tool estimates the global ancestry close to the true estimates but performs better in a three-way simulation than in a five-way simulation. This is expected as the model accuracy decreases with increasing parameter space (Zhou and Stephens, 2012). The PCA and admixture plots indicate that the structure of the simulated populations met the criteria of the population that we required for the downstream analysis.

### Assessing genome association studies

We further examined state-of-the-art and commonly used GWAS tools representing major GWAS models using the simulated GWAS datasets described above. We included linear mixed-model (LMM)-based approaches Efficient Mixed-Model Association eXpedited (EMMAX) (Kang et al., 2010), GCTA (Yang et al., 2011) and genome-wide efficient mixed model association (GEMMA) (Zhou and Stephens, 2012), as well as the most widely used GWAS analysis tools PLINK (Purcell et al., 2007) and SNPTEST (Marchini et al., 2007). For the homogeneous African and European population, we considered the standard PLINK association test under a logistic model that allowed the inclusion of covariates, which we labeled PLINK-Logistic.

For the admixture simulations, we only considered PLINK-Logistic. For GCTA, we considered two association approaches included in the tool. In the first approach, the GRM used includes the chromosome with the SNP being tested for association, which we labeled GCTA, while the second approach uses a GRM that excludes the chromosome that contains the SNP being tested for association, which we label GCTA-LOCO (leave one chromosome out). This approach is an extension of GCTA to eliminate proximal contamination that may be introduced in the association analysis when this chromosome is included in the calculation of the GRM. Similarly, in SNPTEST, we considered both the frequentist association approach (which we refer to as SNPTEST-Frequentist) and the Bayesian approach (which we refer to as SNPTEST-Bayesian). We also assessed TRACTOR (Atkinson et al., 2020; Atkinson et al., 2021) and joint ancestry and SNP association method for a multi-way admixed population (JasMAP) (Mugo et al., 2023), two recently joint SNP and admixture association approaches tailored for admixed populations. TRACTOR and JasMAP require LAIs as input, which we obtained by running RFMIX (Maples et al., 2013). We used RFMIX for ancestry deconvolution as this is the recommended tool by TRACTOR and JasMAP.

First, we obtained the first 10 PCs under each simulated set of data using GCTA. For the homogeneous populations, we included five PCs as covariates when running PLINK-Logistic and SNPTEST; based on the PCA plots, we did not observe any population structure in the homogeneous cohorts. In the admixture populations, however, we included 10 PCs as covariates in the association test to control for global ancestry. No missingness was observed in the datasets, and all the simulated samples were retained for the association analysis.

We considered only common SNPs when running the association tests. We thus ran the association analysis using eight disease-scoring statistics for the homogeneous population and seven for the admixed population. We then obtained the corresponding summary statistics and Manhattan plots. To correct for multiple tests, we used the Bonferroni correction approach. Since the number of SNPs in our homogeneous population was >1, 000, 000, we used a genome-wide significance of 5.0 × 10^−08^ for all the frequentist tests. The significance threshold for the three-way admixed population was 1.57 × 10^−07^; for the five-way admixed population, it was 8.48 × 10^−08^ for the smaller sample size and 8.47 × 10^−08^ for the larger sample size. We used log(BF) of 4.61 as the significant threshold for the Bayes factor (BF) for the SNPTEST-Bayesian test, using Jeffrey’s scale of evidence (Marchini et al., 2007; Kosheleva et al., 2021; Wakefield, 2009; Jeffreys, 1961). JasMAP outputs the posterior probability of association (PPA) as the final summary statistics, and a significance threshold of PPA = 0.5 is used.

### Evaluating European and African simulated GWAS

Results in Supplementary Figures S8–S11 are based on the homogeneous European and African populations, and the corresponding summary statistics for the simulated SNPs with risk effects are displayed in Supplementary Tables S6–S21. In both simulations, we observed that, for all the tools assessed with the small sample size, none detected the signals on chromosome 11. However, for the European population, the LMM-based tools, GEMMA, GCTA, and GCTA-LOCO, and the standard PLINK approach captured the signals on four of the chromosomes, while EMMAX, PLINK-Logistic, and SNPTEST detected significant signals on three of the chromosomes. Despite EMMAX and SNPTEST detecting three out of the five simulated risk regions at this sample size for the European population, they eliminated the SNP with simulated risk effects on chromosome 6 from the analysis as part of internal quality controls, and thus, no significant SNP was observed. In comparison, in the African simulated GWAS dataset with the smaller sample size, we observed that all the tools, except for SNPTEST, were only able to capture the signals on chromosomes 2, 6, and 20 at significant levels and the signals on chromosomes 15 only at marginal significance thresholds.

On increasing the sample size for the European population, we observed that all the tools were able to capture the simulated disease signals, and although EMMAX and SNPTEST excluded the risk SNP on chromosome 6 by internal quality control, SNPs in LD with these risk SNPs were captured for this population, and a significant signal was detected. However, in the African population, the signals at chromosomes 15 and 20 showed improvement with increased sample sizes, but with less significant thresholds compared to the European population. We also note that at chromosome 11, where a weak signal was simulated for the African simulation, even with a larger sample size, the signals were still at a marginal significance threshold with all assessed scoring statistics.

The findings suggested that in a homogeneous European population with small sample sizes, GEMMA, GCTA, GCTA-LOCO, and PLINK were more robust in capturing most of the simulated risk variants at significant levels, with PLINK-Logistic following suit. With large sample sizes, all the tools were effective in capturing the simulated risk at significance levels. We also noted that internal quality control checks implemented in EMMAX, SNPTEST-Frequentist, and SNPTEST-Bayesian might remove risk variants, especially in analyses with small sample sizes, and thus miss out significant associations. In addition, our results revealed that with a small sample size, most tools were underpowered to detect some of the risk variants present at a significant level in the African GWAS, and even with an increased sample size, as observed on chromosome 11, some risk variants struggled to reach the stringent standard GWAS threshold in the presence of a signal. Similar significant thresholds were observed for the risk SNPs simulated on chromosome 2, rs113456069 and rs112486568, in the European and African populations, respectively, and similarly on chromosome 20, rs6115358 and rs57343318, in the European and African populations, respectively. It is noteworthy that only SNPs rs113456069 on chromosome 2 and rs6115358 on chromosome 20 were simulated with strong risk effects in the European dataset, but not in the African data set, while SNPs rs112486568 on chromosome 2 and rs7343318 on chromosome 20 were simulated with strong risk effects in the African population, but not in the European dataset (Figure 2). Although SNPs rs113456069 and rs112486568 on chromosome 2 were simulated to be in high LD in the European population, we observed that these SNPs were also in high LD in the African population. Similarly, SNPs rs6115358 and rs57343318 on chromosome 20 were also in high LD in the African datasets. We thus deduce that if strong risk signals exist in both European and African populations with high-powered studies, cross-population replication is possible using most of the tools assessed. In addition, we noticed that the local LD pulls out several non-risk variants (variants that were not simulated with risk effect) to reach genome-wide significance, suggesting that current tools might not distinguish between background LD (correlation due to nearby markers) and the linkage or correlation to true SNPs with risk effect.

### Assessment of association tests from simulated admixed GWAS data

The association tests of GWAS tools assessed using the three-way admixed population simulation are displayed in Supplementary Figure S12, while Supplementary Tables 22–S28 present the summary statistics of the risk SNPs simulated. LMM-based tools EMMAX, GEMMA, and GCTA performed quite similarly in detecting the simulated risk variants and captured the risk variants on chromosomes 2 and 6 at a significant threshold. These three tools detected the risk variants simulated on chromosomes 11 and 15 at marginal significance thresholds; the SNPs in LD with the risk variant on chromosome 11 were detected as significant. GCTA-LOCO, an LMM-based approach, performed quite similarly to PLINK-Logistic, SNPTEST-Frequentist, and SNPTEST-Bayesian in capturing the risk variants on chromosomes 2, 6, and 11 as significant, while capturing the signal on chromosome 15 at a marginal significance threshold. On chromosome 11, however, we note that the four approaches detected a second region that was not simulated with a risk variant and, thus, a false-positive association that could be due to admixture was observed. The four approaches also captured a significant signal on chromosome 12 that was not simulated as significant but detected at a marginal significance threshold using the other tools. We, therefore, noted that the LMM-based approaches EMMAX, GEMMA, and GCTA were more robust in capturing a wide range of population structures, which enabled them to control for any spurious associations. However, GCTA-LOCO, also an LMM-based approach, was ineffective in capturing the sample structures, and we hypothesize that the LOCO approach might have missed accounting for a significant level of the sample structure in the analysis.

The association tests using five-way admixed population simulation are displayed in Supplementary Figures S13–S14, while Supplementary Tables S29–S35 present the summary statistics of the risk SNPs simulated. We observed that for the small sample size of 500 cases and 500 controls, all the tools could capture the simulated risk variants on chromosomes 2, 6, and 20. However, none of the tools captured the risk variants on chromosomes 11 and 15 at a significant level. With a large sample size, we observed that all the tools could capture one of the risk variants on chromosome 11, but the signal at chromosome 15 could still not reach the significance threshold. We thus noted that when the genotype risk was strong, irrespective of the presence and strength of the ancestry association, all the tools were also able to detect the risk variant at a significant level, as observed on chromosomes 2 and 6 in the three-way simulation and on chromosomes 2, 6, and 20 in the five-way simulation analysis. This was true for most tools, even with the smaller sample size in the five-way simulation analysis. However, when the genotype risk was weak and the ancestry risk present was weak or strong, most of the tools were limited in their ability to detect the simulated risk variant at a significant level, as observed on chromosome 15 in the three-way simulation and on chromosomes 11 and 15 in the five-way simulation.

Although GCTA-LOCO, PLINK-Logistic, SNPTEST-Frequentist, and SNPTEST-Bayesian were able to detect the risk SNPs simulated on chromosome 11 in the three-way admixed simulation at significance thresholds, they were limited in capturing the admixture-LD on this chromosome and resulted in spurious association signals, which GEMMA, EMMAX, and GCTA successfully controlled for; however, they detected this risk variant at marginal significance thresholds. By increasing the sample size, one simulated risk SNP on chromosome 11 in the five-way admixed population association was also detected as significant by all tools. The simulated ancestry risk on this chromosome was weak, which implied that the increase in power to detect the risk variant was highly likely due to the increase in sample size and not associated with ancestry risk.

We observed that TRACTOR performed quite similarly to the PLINK-Logistic, GCTA-LOCO, SNPTEST-Frequentist, and SNPTEST-Bayesian in capturing the simulated risk SNPs in the three-way admixed population (Supplementary Figure S15; Supplementary Table S36) and in the smaller sample size for the five-way admixed population (Supplementary Figures S16, S17; Supplementary Tables S37–S38). JasMAP (Supplementary Figures S18–S20) was able to improve the power to detect the risk SNP as significant when both the genotypic and ancestry risk signals were marginal in both simulated three-way (Supplementary Table S39) and five-way admixed datasets (Supplementary Tables S40, S41). Using the larger sample size, TRACTOR was able to capture the risk region that was close to the simulated risk SNP as significant but not the simulated risk SNP. We noted that for quite a number of ancestry backgrounds, TRACTOR was not able to generate a result for most SNPs, possibly due to the fact that it has not yet been optimized for multi-way admixed populations. Overall, joint association implemented in TRACTOR and JasMAP showed significant improvement in association power when the genotype risk effects are strong, irrespective of the strength of the ancestry risk and sample size (Supplementary Table S41).

Our results support the need for better calibrated methods of association in a multi-way admixed population that control population structure not only at a global level but also at a more local level by incorporating the effect of local ancestry.

### Evaluating the replication of European GWAS in African populations

**FIGURE 3:**
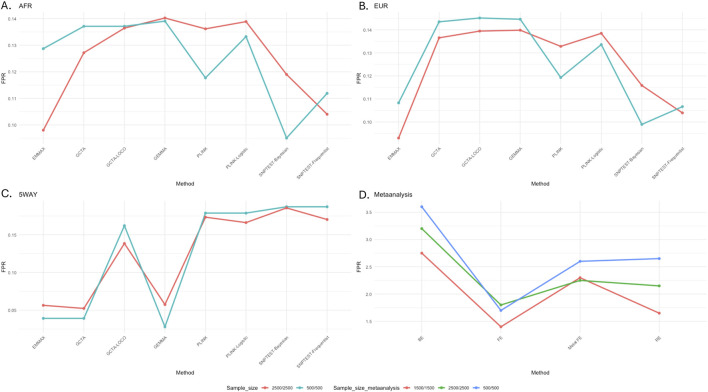
Proportion of non-risk simulated variants reaching genome-wide significance across GWAS tools. **(A)** From African (AFR); **(B)** from European (EUR); **(C)** admixed (five-way simulation); **(D)** cross meta-analysis of AFR and EUR simulated datasets.

To assess the false-positive rate (FPR) for the association analysis for the different tools, using the GWAS summary statistics generated from each tool, we computed the proportion of non-risk SNPs that reached genome-wide significance; FPR = false positive/(false positives + true negatives). Based on our results, all the tools had some percentages of false positives in the association test of the African (Figure 3A), European (Figure 3B), and admixed (Figure 3C) populations. In the simulated European and African populations, FPR seemed to be lower for EMMAX and SNPTEST, but the difference among the tools was very marginal. In the admixture simulation, based on five-way simulated datasets, it was clear that the LMM-based tools, except for GCTA-LOCO, had relatively lower FPR compared to the other tools. By eliminating a substantial number of SNPs in the calculation of the GRM, the LOCO approach possibly missed accounting for a significant level of the population structure in the analysis, which could have resulted in the relatively high FPR.

To evaluate the replication of European GWAS results in African GWAS, we generated three GWAS (sample sizes 500, 1,500, and 2,500) in each of the simulated African and European datasets. For an easier presentation, we focused on these simulated SNPs on chromosomes 2 and 20 (Figure 2) and used the resulting summary statistics from the LMM model EMMAX to carry out meta-analysis across African and European datasets. We assessed the level of false replication by comparing fixed, random, and binary effects (Figure 3D). The results from cross meta-analysis suggest that replication between European and African GWAS is possible if the effect exists across the two populations.

Considering our findings in Supplementary Tables S6–21, we observe that cross-population GWAS replication through meta-analysis is possible and increases with sample size when a similar magnitude of risk effect exists across populations, and a fixed effect can be applied. Random effects are commonly used in meta-analysis as we often do not know the existence of the risk effect; however, our simulation results show that the random effect may suffer from false positives due to heterogeneity and differing population-specific LD patterns (Figure 3D). In addition, the implemented fixed effect in METASOFT (Han and Eskin, 2011) has fewer false positives than that in METAL (Willer et al., 2010). Our recommendation is to use METASOFT as it enables reporting on fixed, random, and binary effects with m-values [posterior distribution (≥0.9) that effects exist in the GWAS cohort] from each GWAS cohort in the meta-analysis.

## What lessons can be drawn from European GWAS to benefit diverse populations?

In the last decade, the increase in human genomic data has led to more than 3,700 GWAS (Welter et al., 2014; Buniello et al., 2019). These studies predicted thousands of genetic risk variants, enabling gene discovery, biological function analysis, and the prediction of the genetic liability of various human phenotypes. The majority of these GWAS have been conducted in European decent populations. The variability in the predictive methods and categorization of functionally relevant genetic variants still pose specific challenges in diverse populations, particularly mixed-ancestry populations (Kim et al., 2018; Shriner et al., 2011a; Shriner et al., 2011b; Shriner, 2017). In addition, GWAS tools and pipelines commonly used in European descent populations may lead to high rates of false-positive/-negative results if stratification is not carefully controlled, particularly in African genomes that harbor the highest genetic diversity and are currently disproportionately under-represented in public databases and reference panels (Buniello et al., 2019). The lesson learnt from various GWAS from European descent populations is that one should consider (i) applying population-specific GWAS pipelines; (ii) choosing appropriate GWAS tool among the existing tools or possibly casting a vote of the association results from running multiple GWAS tools to allow genome-wide level of significance to have a consensus across many tools; (iii) the direction of effect size in each study in meta-analysis with African populations to replicate European GWAS; and (iv) reporting the minor allele frequency, effect size, standard error of the effect size, and LD of the associated variants in African versus non-African populations to enable improved interpretation of the results.

Despite most of the current well-established GWAS tools (Figure 1) being benchmarked using populations of European ancestry, several studies demonstrated that the high genetic diversity found in African and admixed populations makes their genomic studies more likely to detect many novel variants that are yet to be described in current public databases such as the GWAS Catalog (Sirugo and Tishkoff, 2019; Mills and Rahal, 2019; Welter et al., 2014; Buniello et al., 2019) and the Phenotype–Genotype Integrator (PheGenI) (Javed et al., 2014). Due to differing patterns of LD, population-specific allele frequencies, and the proportion of derived/ancestral risk alleles, caution must be used in (1) performing a meta-analysis which combines data from participants across multiple datasets within/between African and non-African populations to analyze millions of variants to increase the power of GWAS and (2) interpreting results from risk prediction and estimation of heritability.

Consistent with other recent studies (Shriner et al., 2011a; Shriner et al., 2011b; Shriner, 2017; Patron et al., 2019), the lesson learnt from various benchmarks of GWAS analysis is that one should consider A) applying significance thresholds and population-specific GWAS pipelines, B) choosing appropriate GWAS tools among the existing tools or considering a consensus approach by possibly running multiple GWAS tools to allow a genome-wide level of significance to have consensus across many tools, C) reporting population-specific minor allele frequency, effect size, standard error of the effect size, and LD of the associated variants in diverse populations to enable improved interpretation of the results, and D) the direction of effect size in each study in meta-analysis with diverse populations to replicate European or other population-specific GWAS.

Although increasing sample size improved the association power for most current GWAS tools, our simulation demonstrated that some risk variants could not reach the genome threshold level. As GWAS extends to diverse populations, the following should be noted:

1) Although increasing sampling may improve the association power, the more the samples, the more genetic variability within such data is swallowed, resulting in current GWAS tools possibly failing to detect some risk variants in large-scale GWAS data (Tam et al., 2019).
2) Diverse or admixture populations may harbor several disease-relevant rare, unique, or population-specific variants compared to Europeans who have undergone population bottlenecks (with more disease-specific common variants); thus, the current GWAS assumption based on “*common disease – common variant*” (Peng and Kimmel, 2007) may have a reduced benefit to diverse populations, mostly characterized by high genetic variation and low LD, or to admixture populations (Tishkoff and Williams, 2002; Martin et al., 2017; Campbell and Tishkoff, 2008; Chimusa et al., 2018; Chimusa et al., 2016; Saint Pierre and Génin, 2014).
3) Despite joint SNP and admixture association tests improving the association power and demonstrating added value beyond standard GWAS tools (Figure 1), they critically rely on accurate LAI, which also requires well-specified founder (reference) populations (Mugo et al., 2023; Atkinson et al., 2020; Atkinson et al., 2021).
4) It remains a challenge to construct appropriate reference or founders’ panels for LAI that accurately characterize admixed populations. A consensus has not yet been reached about best practices for reference panels, including the use of continental versus sub-continental reference populations (Geza et al., 2019).
5) LAI methods in multi-way admixed populations may suffer from spurious deviations in average local ancestry at some chromosomal locations of cases/controls, where the modeled ancestral population is unusually different from the true ancestral population, due to historical actions such as natural selection (Chimusa et al., 2014; Secolin et al., 2019). This is still a serious unresolved weakness of admixture association (Chimusa et al., 2014; Secolin et al., 2019; Mani, 2017) in most multi-way admixed populations, which worsens when two or more reference populations are genetically and closely related, resulting in the ancestry being inaccurately assigned or misassigned to admixed individuals (Chimusa et al., 2014; Mugo et al., 2023; Atkinson et al., 2020; Atkinson et al., 2021; Secolin et al., 2019; Mani, 2017).
6) There are very few joint SNP and admixture tools, possibly because of their multi-stage process requiring (A) improved LAI accuracy (Mugo et al., 2023; Atkinson et al., 2020; Atkinson et al., 2021); (B) building integrative software for running multi-way admixture deconvolution analysis (Geza et al., 2019); (C) persisting dilemma in modeling effect sizes conditional on local ancestry, resulting in significant reduction in association power (Mester et al., 2023).
7) It is critical to develop new or adapted pipelines for diverse genetic data or to evaluate existing bioinformatics pipeline tools using diverse populations to account for diverse genetic and environmental characteristics that could differently shape phenotypic variation.

## Concluding remarks and future perspectives

GWAS have significantly contributed to medical genomics and understanding of complex traits; however, large numbers of false positives and the small effect size of genetic risk variants have induced a need for calibrated sample sizes and a culture of FAIR (findability, accessibility, interoperability, and reusability) data and sharing. Although GWAS has not yet fully translated into an ability to predict phenotypes in real-world applications based on genetic markers, polygenic and transcriptional risk scores (PRSs and TRSs) for complex diseases hold potential for stratification according to risk, and there is a critical need for new approaches, methodologies, and diverse large data to address questions about the genetic architecture of complex traits and applicability of findings to clinical settings. In this study, we leveraged FractalSIM to generate simulated GWAS datasets mimicking European, African, and admixed populations to evaluate commonly used GWAS tools, as well as newly joint SNP association and admixture tools on their performance, using our simulated GWAS datasets. Our results suggested that LMM-based tools were more robust in capturing risk variants present in the European population with smaller samples but with increased samples in African and admixed populations. All the tools performed similarly and were limited in their ability to capture risk variants present in small sample sizes when using simulated African-specific data. Although increasing the sample size did improve the power to capture risk variants, when the signal was weak, some risk variants still struggled to reach the significance levels set in GWAS. Given the increased frequency of independent testing in simulated African population GWAS analysis due to the generally higher number of SNPs and short LD blocks, it has been suggested and shown that a stricter significance threshold should be considered (Choi et al., 2020). In considering this, it raises the question of whether the risk signals observed at the near marginal significance thresholds in the African GWAS simulated datasets with increased sample sizes would still be significant with more stringent thresholds. This, therefore, emphasizes the dire need for increased sampling in African populations if African GWAS is to catch up with European GWAS, given that small sample sizes still pose a limitation for African GWAS.

When the genotype risk was weak in the presence of a strong ancestry risk, the association between joint SNPs and admixture was successful in leveraging the ancestry risk to enhance the power to detect the signal even when the sample size was small. We observed that joint associations implemented in TRACTOR and JasMAP are calibrated for admixed populations and have significantly improved the association’s power in detecting risk effect signals when the genotype risk was strong, irrespective of the strength of the ancestry risk and sample size.

We observed that cross-population replication in the presence of strong risk signals in both European and African populations is possible when applying most of the current state-of-the-art tools from homogeneous population-based association analysis, provided the studies are high-powered. However, caution should be exercised while using EMMAX, SNPTEST-Frequentist, and SNPTEST-Bayesian approaches as internal quality control procedures in these tools may eliminate risk variants from the analysis. Furthermore, we observed that the LMM-based models, except for GCTA-LOCO, performed better at controlling for spurious associations in the admixture context. However, they were limited in detecting the simulated risk variant when the genotype risk was very weak, irrespective of whether the ancestry risk was very high or moderate at the genomic region containing the risk SNP.

Overall, GWAS reproducibility is critical, and it is also important to keep fostering a culture of replication to maintain reliability in findings. As sequencing and, consequently, the availability of genomic information on African populations increases, there are new opportunities to design next-generation disease scoring statistical models that capture not only common variants but also rare and population-specific variants. These new approaches should be tailored to and leverage the characterization of diverse populations with longer histories, high genetic diversity, and environmental heterogeneity, as well as varying types of LD patterns. This will enable us to better understand and elucidate the genetic architecture of African complex traits, variation in drug/treatment response, and disease outcomes. There are potentially many uses of novel disease-scoring statistics models that further leverage the environmental diversity, such as on the African continent, including a better understanding of the pathogenesis of diseases of global health relevance, new leads for studying underlying risk prediction, and advancing clinical prediction of global treatment. The findings from these African-specific disease scoring statistics on African data will pave the way for a new, more diverse research dimension in public health translation^1^ ^,^ ^2^ ^,^ ^3^ ^,^ ^4^ ^,^ ^5^ ^,^ ^6^ ^,^ ^7^ ^,^ ^8^ ^,^ ^9^ ^,^ ^10^ ^,^ ^11^ ^,^ ^12^ ^,^ ^13^ ^,^ ^14^ ^,^ ^15^ ^,^ ^16^ ^,^ ^17^ ^,^ ^18^ ^,^ ^19^.

## Data Availability

The original contributions presented in the study are included in the article/Supplementary Material; further inquiries can be directed to the corresponding author.

## Footnotes

1 TASSEL, https://www.maizegenetics.net/tassel

2 PLINK, https://www.cog-genomics.org/plink/

3 SNPTEST, https://www.chg.ox.ac.uk/∼gav/snptest/

4 EMMA, https://academic.oup.com/genetics/article/178/3/1709/6,061,473

5 EMMAX, https://genome.sph.umich.edu/wiki/EMMAX

6 GCTA, https://yanglab.westlake.edu.cn/software/gcta/#Overview

7 GEMMA, https://github.com/genetics-statistics/GEMMA

8 FaST-LMM, https://fastlmm.github.io/

9 GWAStools, https://www.bioconductor.org/packages/release/bioc/html/GWASTools.html

10 Grammar-Gamma, https://lomereiter.github.io/2015/03/27/overview.html

11 SKAT, http://www.hsph.harvard.edu/∼xlin/software.html

12 EPIQ, https://bioinformaticshome.com/tools/gwas/descriptions/EPIQ.html#gsc.tab=0

13 QTDT, https://bioinformaticshome.com/tools/gwas/descriptions/QTDT.html#gsc.tab=0

14 PheWAS, https://phewascatalog.org/phewas/#home

15 BOLT-LMM, https://alkesgroup.broadinstitute.org/BOLT-LMM/BOLT-LMM_manual.html

16 ancGWAS, https://github.com/echimusa/ancGWAS

17 LDAK, https://dougspeed.com/downloads/

18 GWAS 4D, http://www.mulinlab.org/gwas4d/gwas4d/gwas4d/gwas4d_about

19 RAINBOW, https://github.com/KosukeHamazaki/RAINBOWR

## Abbreviations

EMMAX, Efficient Mixed-Model Association eXpedited; FAIR, findability, accessibility, interoperability, and reusability; FPR, false-positive rate; GEMMA, genome-wide efficient mixed model association; GCTA, genome-wide complex trait analysis; GWAS, genome-wide association studies; JasMAP, joint ancestry and SNP association method for a multi-way admixed population; LAI, local ancestry inference; LD, linkage disequilibrium; LMM, linear mixed model; LOCO, leave one chromosome out; PC(s), principal components; PCA, principal component analysis; PheGenI, Phenotype–Genotype Integrator; PRS, polygenic risk score; SNP, single-nucleotide polymorphism; TRS, transcriptional risk score.
