# Supplementary material for: Data simulation to optimize frameworks for genome-wide association studies in diverse populations
Source: Front Genet. 2025 Jun 18;16:1559496. doi: 10.3389/fgene.2025.1559496 (PMC12213643; doi:10.3389/fgene.2025.1559496)
Supplement: Supplementary file 1 [file Supplementaryfile1.pdf]

Data Simulation to Optimize the GWAS Framework in Diverse Populations

Jacquiline Wangui Mugo[1], Nicola Mulder[1] and Emile Rugamika Chimusa[2*]

[*]correspondence:emile.chimusa@northumbria.ac.uk

Affiliations:

1. Department of Integrative Biomedical Sciences, Computational Biology Division, Institute of Infectious Disease and Molecular Medicine, University of Cape Town, Medical School, Observatory, Cape Town 7925, South Africa.

2. Department of Applied Sciences, Faculty of Health and Life Sciences, Northumbria University, Newcastle, Tyne and Wear, NE1 8ST, United Kingdom.

0.1 Methods

0.1.1 Leveraging Data Simulation Framework to Dissect GWAS in Diverse Populations

Table 1: The table lists the different populations, their abbreviations, and the corresponding sample sizes for the populations merged to obtain the reference population for the simulation of European and African populations.

**Simulation**	**Reference Population**	**Sample Size**
European	British (GBR)	85
	Iberian Spanish (IBS)	107
	Finnish (FIN)	99
	Toscani in Italy (TSI)	104
	Utah residents with Northern and Western European ancestry (CEU)	94
	**Merged European reference**	**489**
African	Gambian Mandinka (GWD)	113
	Yoruba (YRI)	108
	**Merged African reference**	**221**

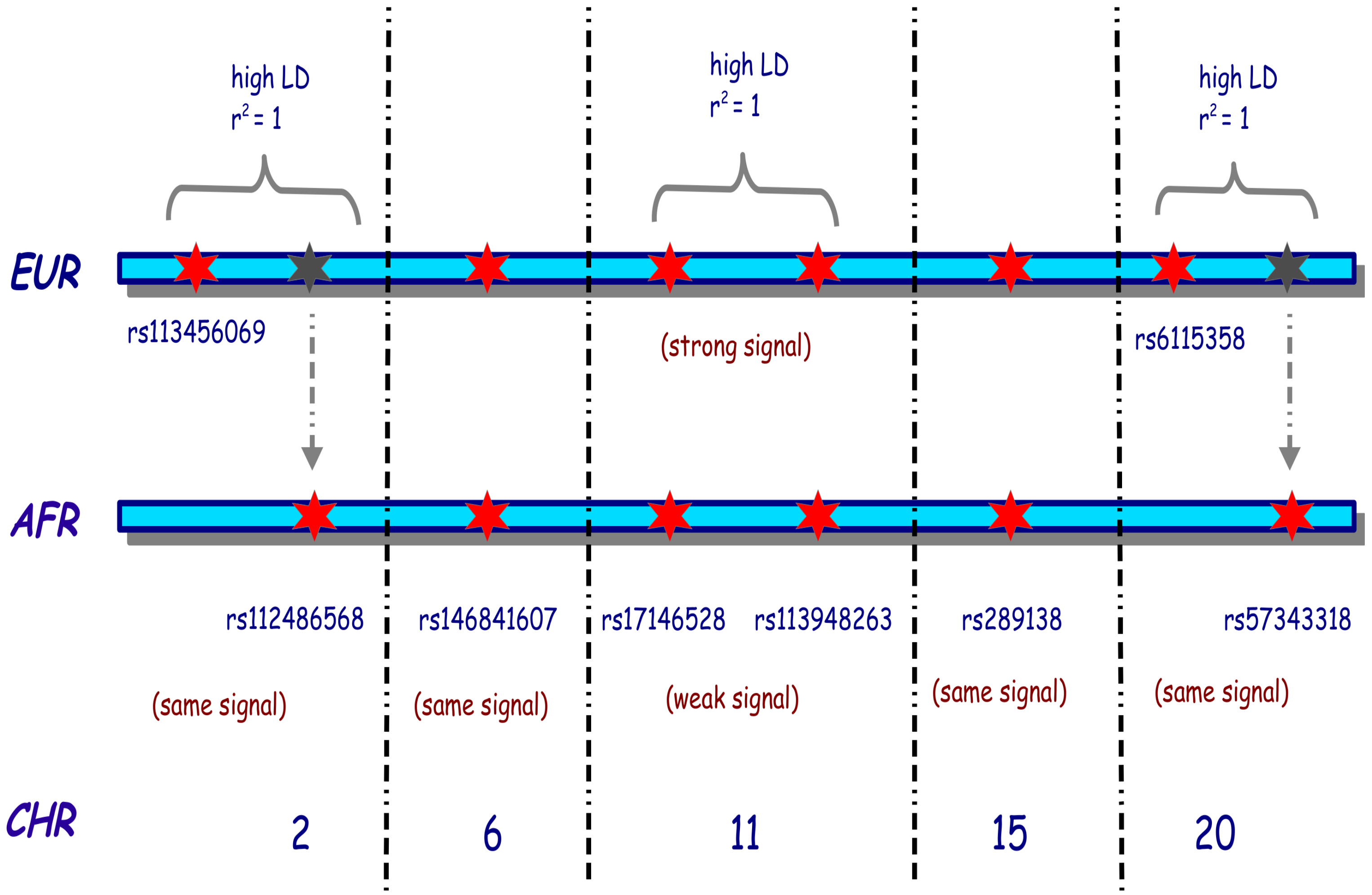

Figure 1: The figure illustrates the choice of the risk SNPs selected for the simulation of homogeneous European and African populations. EUR indicates European, AFR indicates African, and CHR indicates the chromosome. A red star indicates the SNP was simulated as a risk SNP in that population. In contrast, a grey star indicates a SNP is present in the European population, in high LD with the risk SNP simulated on that chromosome, but simulated as a risk SNP in the African population. The black dotted vertical lines indicate the presence of other chromosomes between the two chromosomes. The risk SNPs on chromosomes 2, 6, 15, and 20 were simulated with the same risk strength in both populations, while on chromosome 11, the risk SNPs of Europeans were simulated with a strong risk signal, while those of Africans were simulated with a weak risk signal.

Table 2: The table lists the simulated risk SNPs and their corresponding homozygosity (HOM) and heterozygosity (HET) risks specified during simulation; - indicates the SNP was not simulated in that population.

**Chr**	**rsID**	**Position**	**European Population**		**African Population**	
			**HOM**	**HET**	**HOM**	**HET**
2	*rs*113456069	113842451	2.5004	0.124	-	-
2	*rs*112486568	113842455	-	-	2.5004	0.124
6	*rs*146841607	29942575	2.5004	0.051	2.5004	0.051
11	*rs*17146528	64732006	1.5004	0.304	1.5004	0.704
11	*rs*113948263	64733224	1.5004	0.304	1.5004	0.704
15	*rs*289138	62599775	1.5004	0.104	1.5004	0.104
20	*rs*6115358	25856361	1.5004	0.304	-	-
20	*rs*7343318	25856699	-	-	0.5004	0.304

Table 3: The table provides information on the parental reference populations for the 3-way and 5-way admixture simulations, their abbreviations, initial sample sizes, and the percentage of ancestry each population contributed in each scenario.

**Simulation**	**Parental Population**	**Sample Size**	**Ancestry Proportion**
3-way	Utah residents with Northern and Western European ancestry (CEU)	94	20%
	Han Chinese from Beijing, China (CHB)	103	10%
	Yoruba (YRI)	108	70%
5-way	Europeans (EUR)	305	15%
	South Asians (SAS)	386	35%
	East Asians (EAS)	441	10%
	Other African ancestries (MAFR)	256	10%
	West Africans (WAFR)	405	30%

Table 4: A list of simulated risk SNPs and the corresponding homozygosity (HOM) and heterozygosity (HET) relative risks specified during the isolated simulation of Europeans (EUR), East Asians (EAS), West Africans (WAFR), South Asians (SAS), and other African populations (MAFR) before the admixture process.

**3-way Simulation**								
**Chr**	**rsID**	**Position**	**CEU**		**CHB**		**YRI**	
			**HOM**	**HET**	**HOM**	**HET**	**HOM**	**HET**
2	*rs*76091761	119924776	2.5004	0.054	2.5004	0.054	2.5004	0.054
6	*rs*79354975	78841154	2.5004	0.054	2.5004	0.054	2.5004	0.054
11	*rs*73417185	4119431	2.5004	0.054	2.5004	0.054	2.5004	0.054
15	*rs*2960806	60169020	2.5004	0.054	2.5004	0.054	2.5004	0.054

**5-way Simulation**												
**Chr**	**rsID**	**Position**	**EUR**		**EAS**		**MAFR**		**SAS**		**WAFR**	
			**HOM**	**HET**	**HOM**	**HET**	**HOM**	**HET**	**HOM**	**HET**	**HOM**	**HET**
2	*rs*13410964	113843283	1.2	2.44	1.0	1.0	1.2	2.44	1.0	1.0	1.0	1.0
2	*rs*17042838	113843337	2.20	2.405	1.0	1.0	2.20	2.405	1.0	1.0	1.0	1.0
6	*rs*2232238	29942857	2.20	2.405	2.20	2.405	2.20	2.405	2.20	2.405	2.20	2.405
11	*rs*7106136	64748278	1.0	1.0	2.33	2.33	1.0	1.0	1.0	1.0	1.0	1.0
11	*rs*10897540	64757496	1.0	1.0	2.33	2.33	1.0	1.0	1.0	1.0	1.0	1.0
15	*rs*11853943	48779402	2.33	2.33	1.0	1.0	1.0	1.0	1.0	1.0	1.0	1.0
20	*rs*6115375	25871801	2.0	2.24	2.0	2.24	2.0	2.24	2.0	2.24	2.0	2.24
20	*rs*6107104	25922993	2.0	2.005	2.0	2.005	2.0	2.005	2.0	2.005	2.0	2.005

Table 5: The table lists the disease risk scenarios simulated in the 3-way and 5-way admixture simulations and the chromosomes containing the risk SNP. ✓ indicates a strong risk was simulated, (✓) indicates a weak risk was simulated, while ✗ indicates no risk was simulated.

**Simulation**	**Chromosome**	**Genotype Risk**	**Ancestry Risk**
3-way	2	✓	✓
	6	✓	✓
	11	(✓)	✓
	15	(✓)	(✓)
	Others	✗	✗
5-way	2	✓	✓
	6 & 20	✓	✗
	11	(✓)	(✓)
	15	(✓)	(✓)
	Others	✗	✗

0.1.2 Assessing Simulated GWAS Data Sets through Population Structure

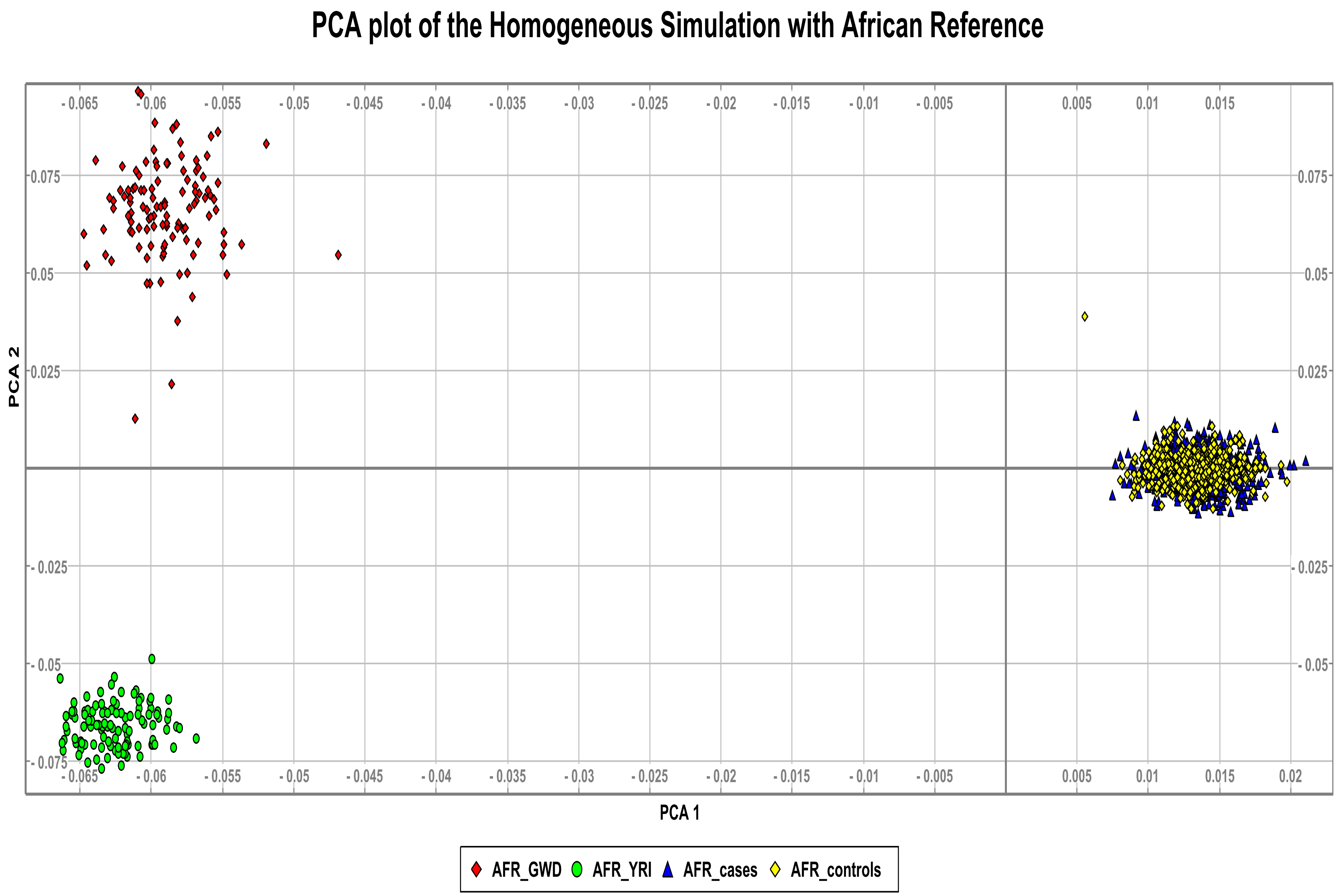

Figure 2: PCA plot of the simulated African population (500 cases and 500 controls) and the corresponding reference populations used in the simulation. AFR_GWD indicates the Gambian Mandinka, AFR_YRI indicates the Yoruba population, while AFR_cases and AFR_controls are the African simulated cases and controls.

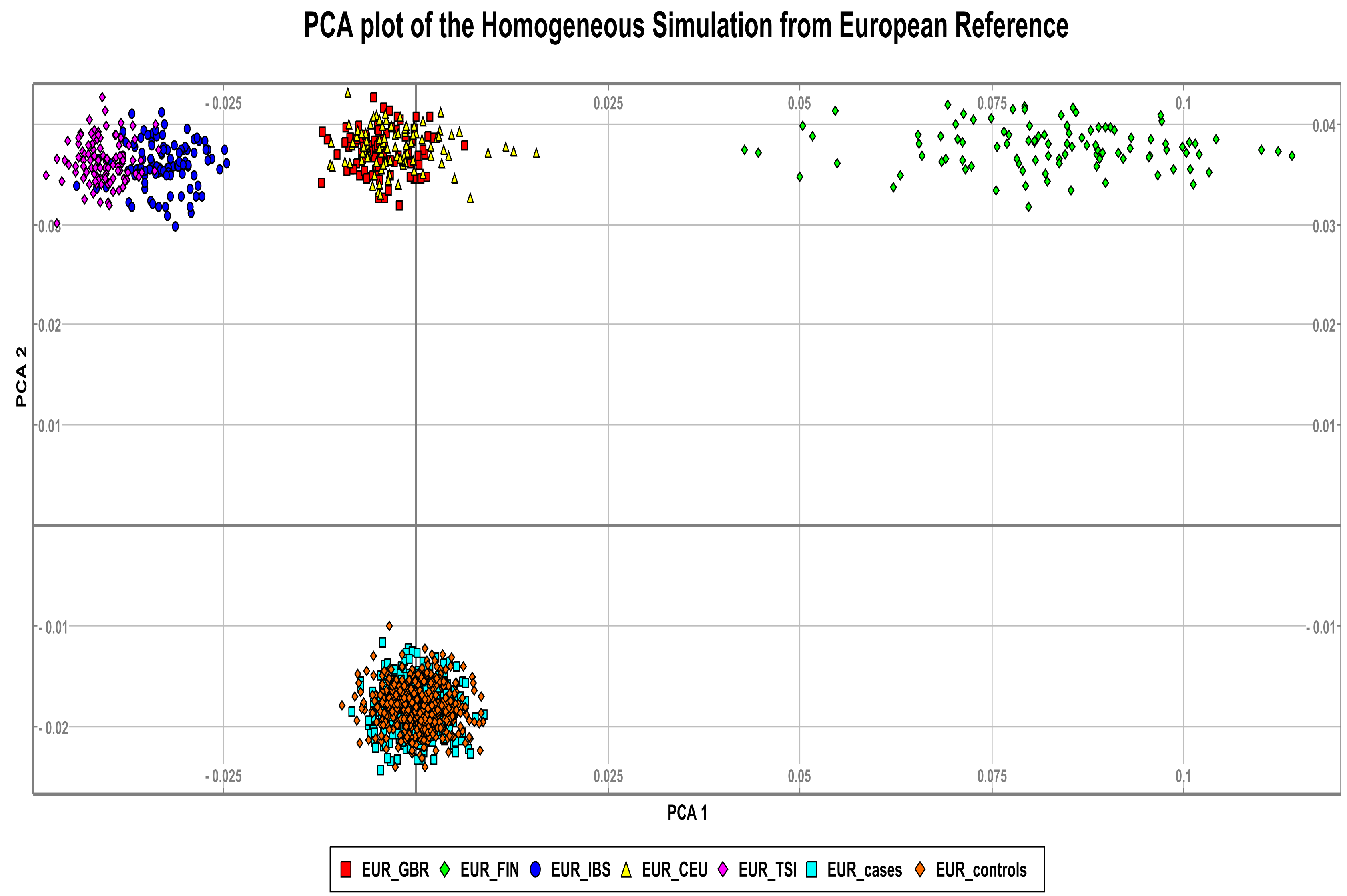

Figure 3: PCA plot of the simulated European population (500 cases and 500 controls) and the reference populations used in the simulation. EUR_GBR are the British, EUR_FIN are the Finnish, EUR_IBS are the Iberian Spanish, EUR_CEU are the Utah residents with Northern and Western European ancestry, and EUR_TSI are the Toscani in Italy. The EUR_cases and the EUR_controls indicate the simulated European population.

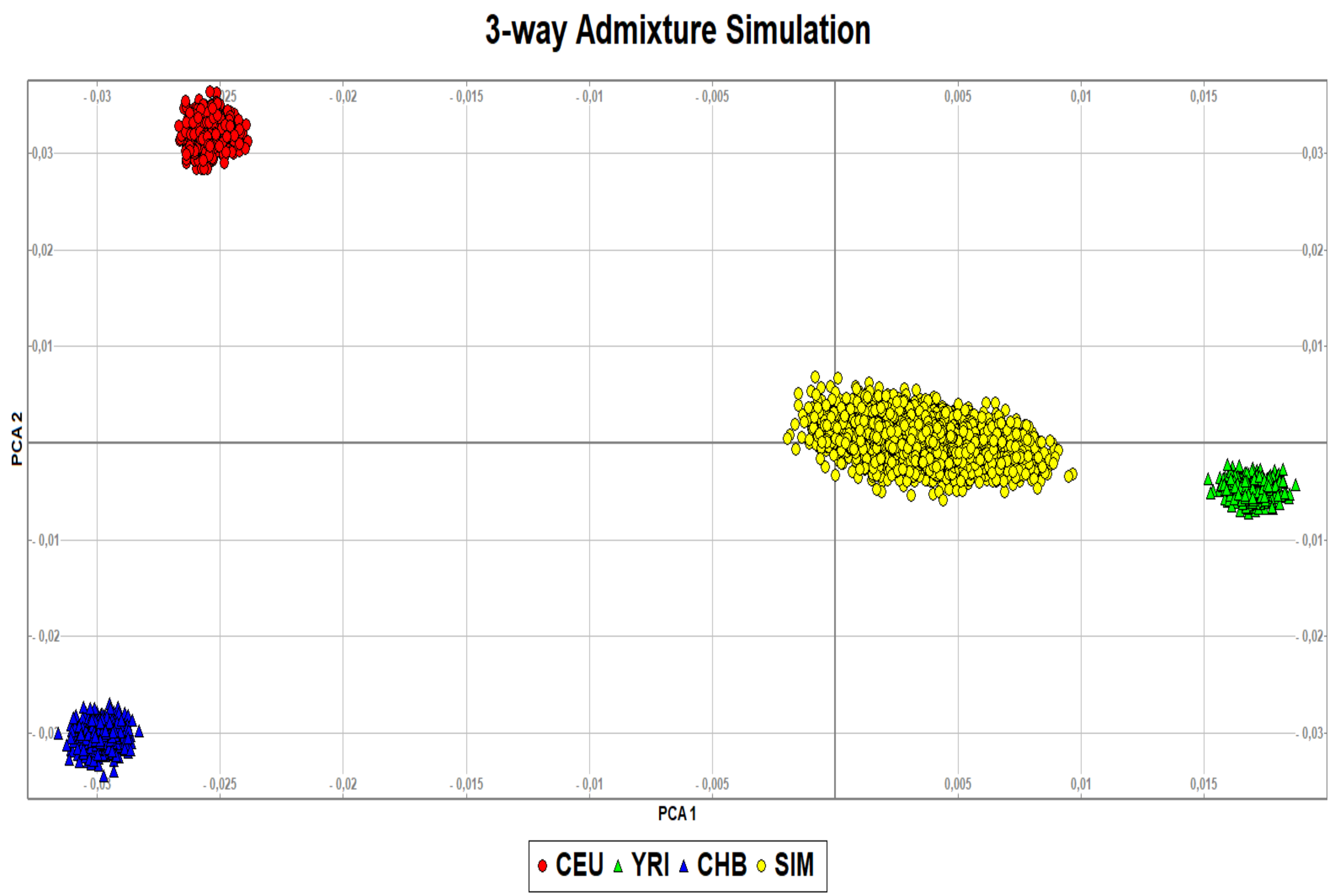

Figure 4: PCA plot of the 3-way admixture simulated population (2500 cases and 2500 controls) and the corresponding CEU, CHB, and YRI simulated in isolation from the reference populations. The simulated admixed population is labeled SIM.

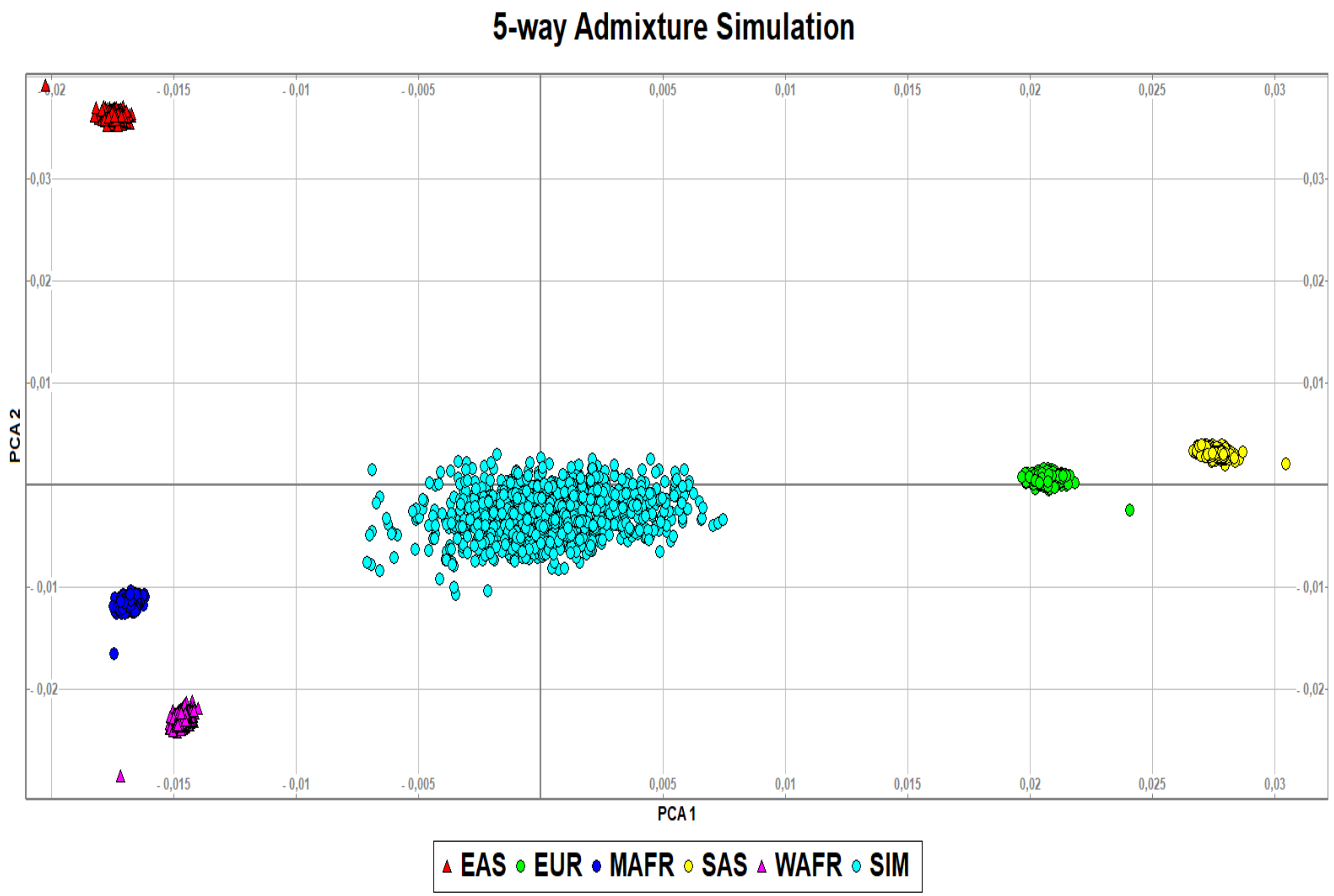

Figure 5: PCA plot of the 5-way admixed population simulation of 500 cases and 500 controls. SIM is the admixed group, while EAS, EUR, MAFR, SAS, and WAFR are the parental populations simulated in isolation from the reference ancestral populations.

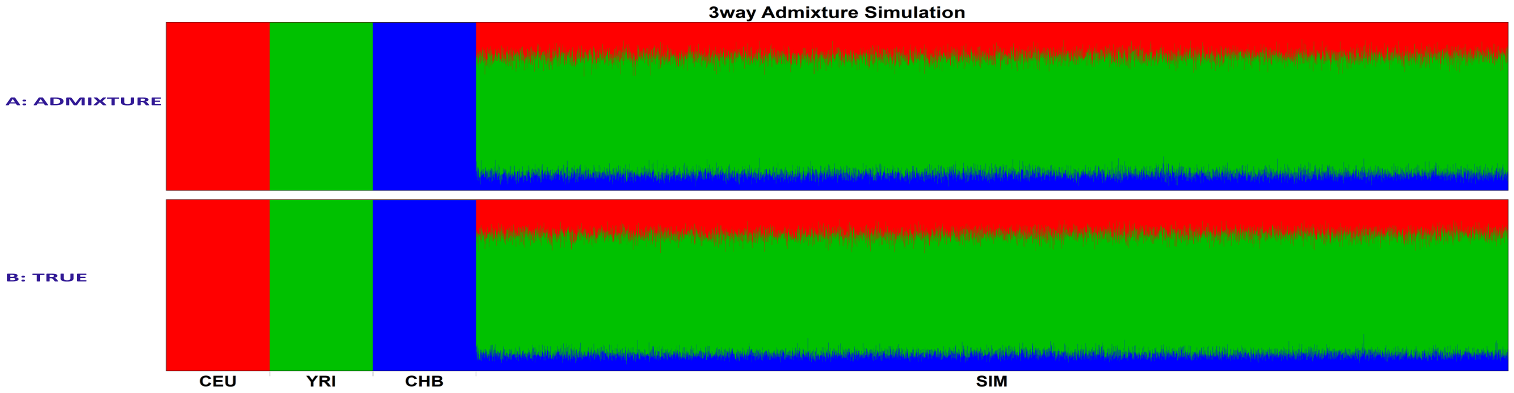

Figure 6: The admixture plot of the 3-way admixture simulated population (SIM), comprised of 2500 cases and 2500 controls, and the corresponding CEU, YRI, and CHB reference populations used in the simulation. A: is the admixture block estimated by the ADMIXTURE tool and B: is the admixture block of the true simulated global ancestry proportion calculated from the ancestry block output by FractalSIM.

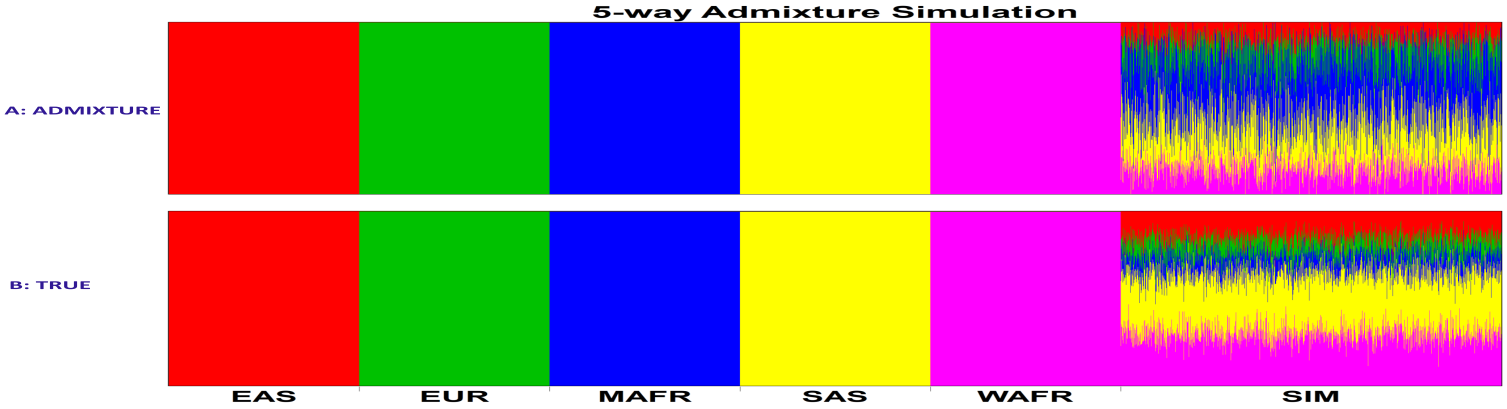

Figure 7: The admixture plot of the 5-way admixture simulation population (SIM), including 500 cases and 500 controls, and the respective EAS, EUR, MAFR, SAS, and WAFR parental populations. A: is the estimated admixture block produced by the ADMIXTURE tool, while B: is the estimated admixture block of the true simulated global ancestry proportion generated by FractalSIM.

0.1.3 Association Analysis of the Simulated Populations

0.2 Results

0.2.1 Assessment of the European and African Simulation GWAS Analysis.

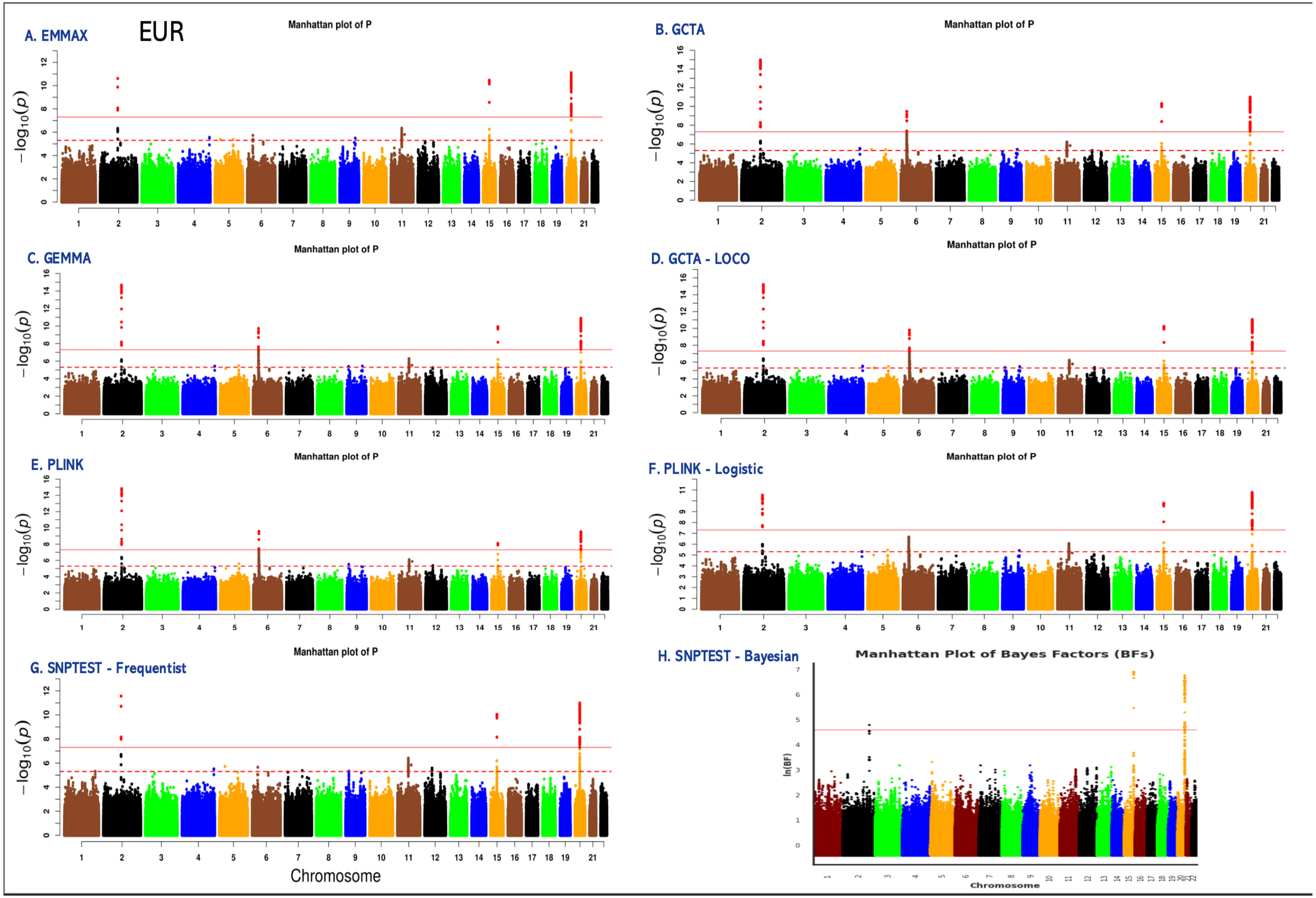

Figure 8: Manhattan plot of the GWAS summary statistics of the homogeneous simulation of 500 cases and 500 controls from the European reference population for 8 disease scoring statistics, corresponding to 5 tools. The red line indicates the GWAS significance level.

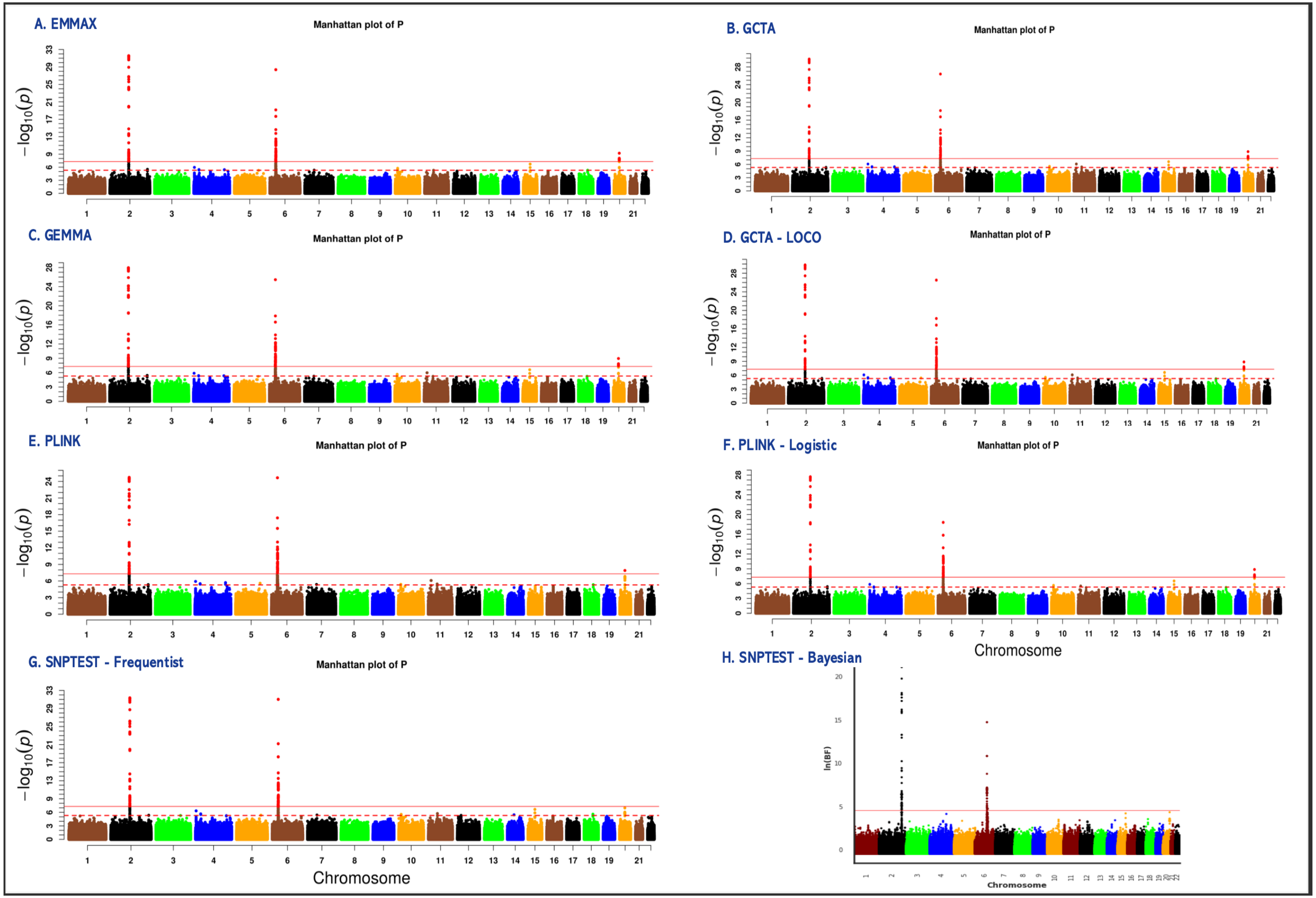

Figure 9: Manhattan plot of the GWAS summary statistics of the homogeneous simulation of 500 cases and 500 controls from the African reference population for 8 disease scoring statistics, corresponding to 5 tools. The red line indicates the GWAS significance level.

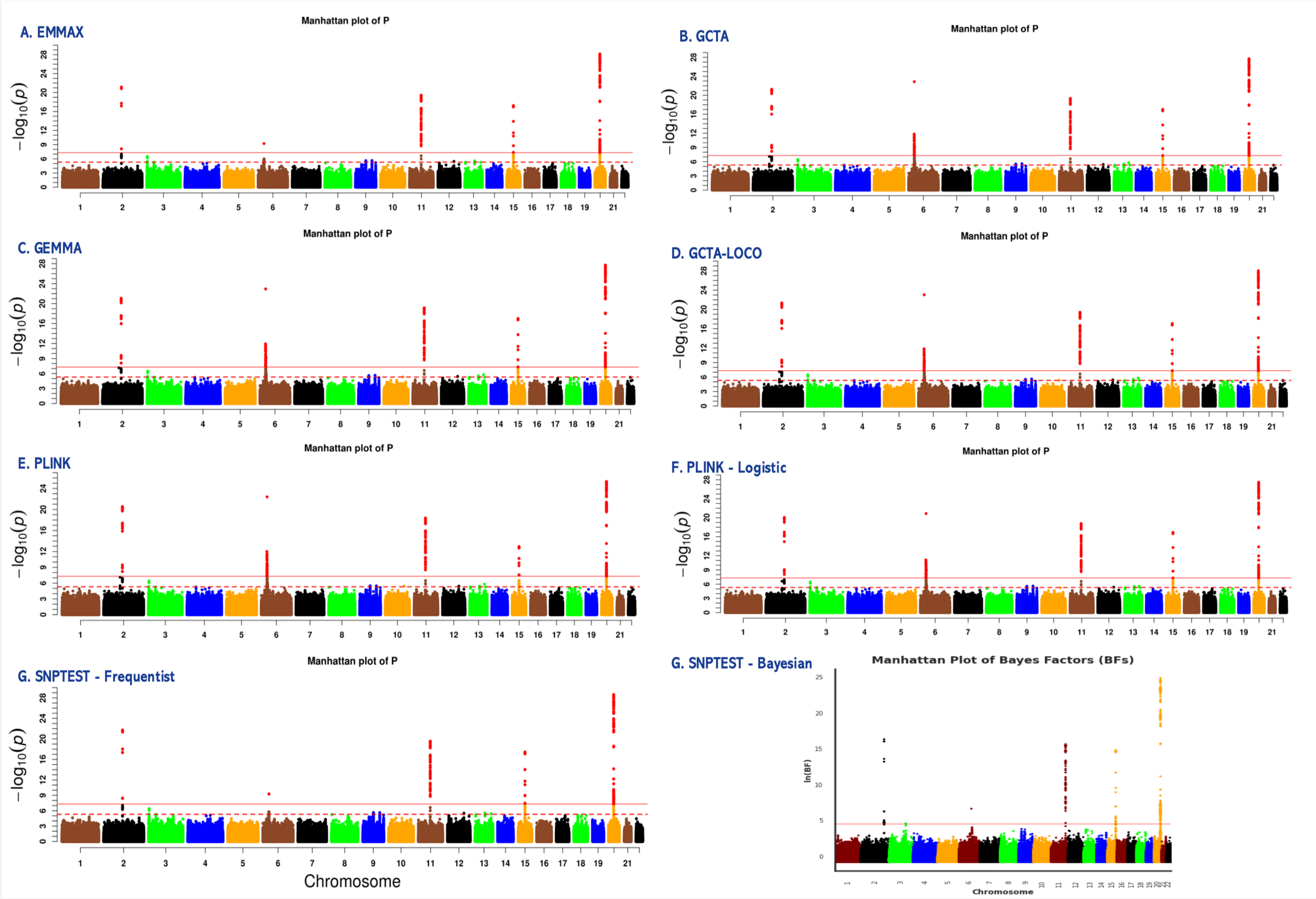

Figure 10: Manhattan plot of the GWAS summary statistics of the homogeneous simulation of 2500 cases and 2500 controls from the European reference population for 8 disease scoring statistics, corresponding to 5 tools. The red line indicates the GWAS significance level.

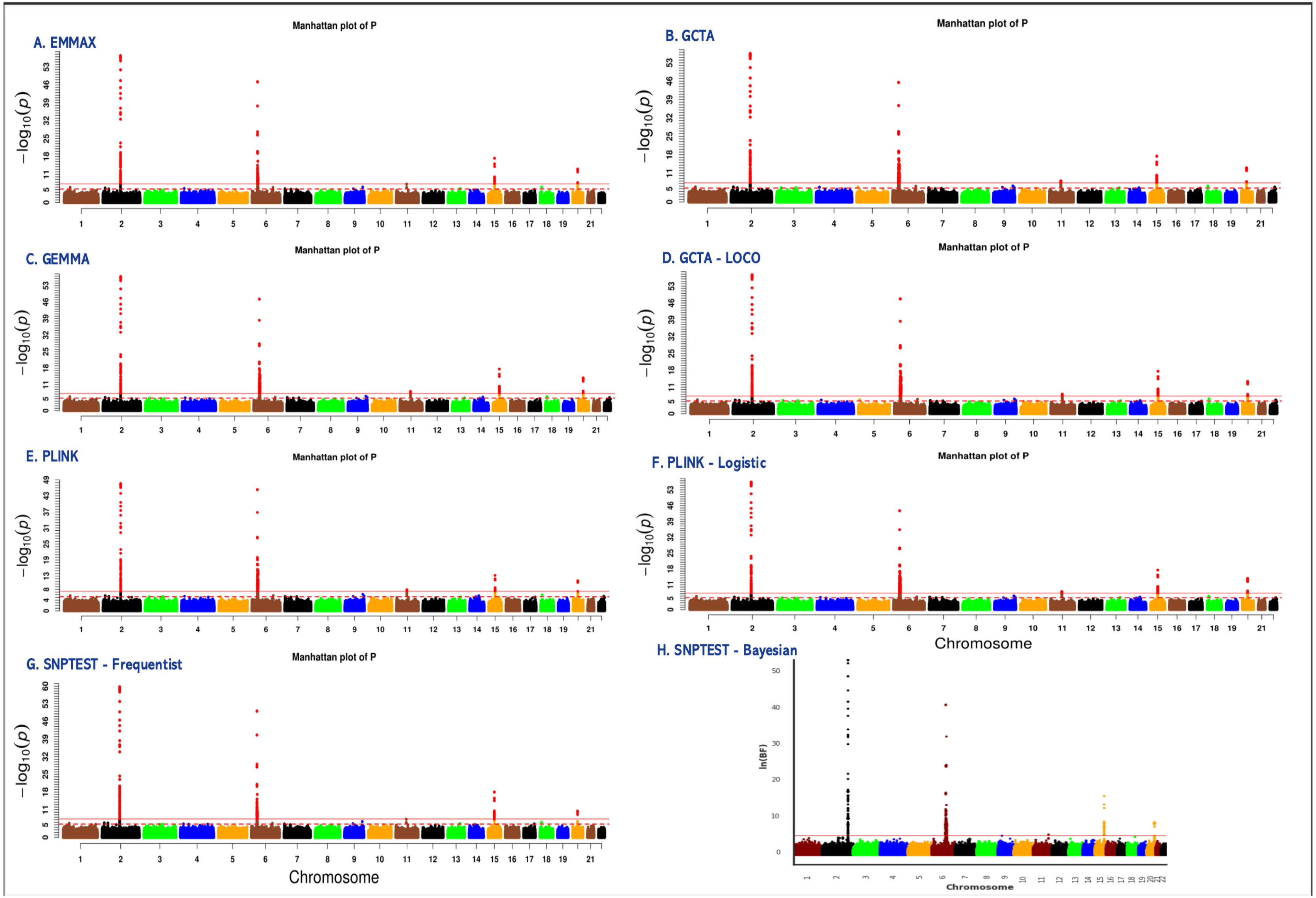

Figure 11: Manhattan plot of the GWAS summary statistics of the homogeneous simulation of 2500 cases and 2500 controls from the African reference population for 8 disease scoring statistics, corresponding to 5 tools. The red line indicates the GWAS significance level.

Table 6: GWAS summary statistics from EMMAX of the simulated risk SNPs in the homogeneous simulation of 500 and 2500 samples from a European reference population of 489 samples. $\mathbf{x(c/c)}$ refers to the $\mathbf{x}$ number of cases and controls.

**EMMAX (European Reference Population)**								
**Chr**	**rsID**	**Position**	**A1**	**A2**	**500 (c/c)**		**2500 (c/c)**	
					**OR**	**p-value**	**OR**	**p-value**
2	*rs*113456069	113842451	C	T	-	-	0.8002	1.2765×10^{-21}
2	*rs*112486568	113842455	G	A	-	-	-	-
6	*rs*146841607	29942575	A	G	-	-	-	-
11	*rs*17146528	64732006	A	G	0.8314	4.07188×10^{-06}	0.8402	5.44133×10^{-20}
11	*rs*113948263	64733224	T	C	0.8314	4.07188×10^{-06}	0.8399	5.00254×10^{-20}
15	*rs*289138	62599775	T	C	1.1797	3.50295×10^{-11}	1.1044	8.98615×10^{-18}
20	*rs*6115358	25856361	C	T	0.8108	2.25442×10^{-11}	0.8597	1.85633×10^{-28}
20	*rs*7343318	25856699	T	C	0.8107	2.3909×10^{-11}	0.8589	7.83493×10^{-28}

Table 7: GWAS summary statistics from GCTA of the simulated risk SNPs in the homogeneous simulation of 500 and 2500 samples from a European reference population of 489 samples. $\mathbf{x(c/c)}$ refers to the $\mathbf{x}$ number of cases and controls.

**GCTA**										
**Chr**	**rsID**	**Position**	**A1**	**A2**	**500 (c/c)**			**2500 (c/c)**		
					**OR(95% C.I.)**	**stderr**	**p-value**	**OR(95% C.I.)**	**stderr**	**p-value**
2	*rs*113456069	113842451	C	T	[1.441, 1.657]	0.0551	1.9126×10^{-15}	[1.205, 1.297]	0.02346	1.552×10^{-21}
2	*rs*112486568	113842455	G	A	[1.441, 1.657]	0.0551	1.9126×10^{-15}	[1.207, 1.300]	0.02352	7.611×10^{-21}
6	*rs*146841607	29942575	A	G	[1.406, 1.676]	0.06891	3.4439×10^{-10}	[1.269, 1.379]	0.0280	1.365×10^{-23}
11	*rs*17146528	64732006	A	G	[1.122, 1.279]	0.04016	5.4182×10^{-06}	[1.153, 1.228]	0.0191	7.0233×10^{-20}
11	*rs*113948263	64733224	T	C	[1.122, 1.279]	0.04016	5.4182×10^{-06}	[1.153, 1.228]	0.0191	6.381×10^{-20}
15	*rs*289138	62599775	T	C	[0.798, 0.897]	0.02514	4.9718×10^{-11}	[0.883, 0.929]	0.0116	1.487×10^{-17}
20	*rs*6115358	25856361	C	T	[1.171, 1.295]	0.0315	2.7381×10^{-11}	[1.136, 1.19]	0.0138	5.261×10^{-28}
20	*rs*7343318	25856699	T	C	[1.171, 1.295]	0.0315	2.9220e-11	[1.137, 1.191]	0.0137	2.256×10^{-28}

Table 8: GWAS summary statistics from GCTA - LOCO of the simulated risk SNPs in the homogeneous simulation of 500 and 2500 samples from a European reference population of 489 samples. $\mathbf{x(c/c)}$ refers to the $\mathbf{x}$ number of cases and controls.

GCTA - LOCO										
**Chr**	**rsID**	**Position**	**A1**	**A2**	**500 (c/c)**			**2500 (c/c)**		
					**OR(95% C.I.)**	**stderr**	**p-value**	**OR(95% C.I.)**	**stderr**	**p-value**
2	*rs*113456069	113842451	C	T	[1.445, 1.66]	0.0549	1.1085×10^{-15}	[1.205, 1.296]	0.02341	1.323×10^{-21}
2	*rs*112486568	113842455	G	A	[1.445, 1.66]	0.0549	1.1085×10^{-15}	[1.207, 1.299]	0.02347	6.479×10^{-22}
6	*rs*146841607	29942575	A	G	[1.413, 1.681]	0.0682	1.5787×10^{-10}	[1.269, 1.379]	0.0279	9.079×10^{-24}
11	*rs*17146528	64732006	A	G	[1.121, 1.278]	0.03998	5.4998×10^{-06}	[1.153, 1.228]	0.01906	5.827×10^{-20}
11	*rs*113948263	64733224	T	C	[1.121, 1.278]	0.03998	5.4998×10^{-06}	[1.153, 1.228]	0.0191	5.301×10^{-20}
15	*rs*289138	62599775	T	C	[0.799, 0.898]	0.0252	5.981×10^{-11}	[0.883, 0.928]	0.01159	1.357×10^{-17}
20	*rs*6115358	25856361	C	T	[1.171, 1.293]	0.0312	2.292×10^{-11}	[1.136, 1.19]	0.0137	2.607×10^{-28}
20	*rs*7343318	25856699	T	C	[1.171, 1.294]	0.0313	2.244×10^{-11}	[1.137, 1.191]	0.01369	1.111×10^{-28}

Table 9: GWAS summary statistics from PLINK of the simulated risk SNPs in the homogeneous simulation of 500 and 2500 samples from a European reference population of 489 samples. $\mathbf{x(c/c)}$ refers to the $\mathbf{x}$ number of cases and controls.

PLINK								
**Chr**	**rsID**	**Position**	**A1**	**A2**	**500 (c/c)**		**2500 (c/c)**	
					**OR**	**p-value**	**OR**	**p-value**
2	*rs*113456069	113842451	C	T	10.93	2.45×10^{-15}	2.483	7.961×10^{-21}
2	*rs*113456069	113842451	C	T	10.93	2.45×10^{-15}	5.34	7.961×10^{-21}
6	*rs*146841607	29942575	A	G	10.92	2.685×10^{-10}	3.345	3.995×10^{-23}
11	*rs*17146528	64732006	A	G	2.129	5.162×10^{-06}	1.97	6.266×10^{-19}
11	*rs*113948263	64733224	T	C	2.129	5.162×10^{-06}	1.973	5.712×10^{-19}
15	*rs*289138	62599775	T	C	0.5949	7.858×10^{-09}	0.7445	1.823×10^{-13}
20	*rs*6115358	25856361	C	T	2.072	6.907×10^{-10}	1.734	1.315×10^{-25}
20	*rs*7343318	25856699	T	C	2.086	5.854×10^{-10}	1.74	6.496×10^{-26}

Table 10: GWAS summary statistics from PLINK (Logistic) of the simulated risk SNPs in the homogeneous simulation of 500 and 2500 samples from a European reference population of 489 samples. $\mathbf{x(c/c)}$ refers to the $\mathbf{x}$ number of cases and controls.

PLINK (Logistic)								
**Chr**	**rsID**	**Position**	**A1**	**A2**	**500 (c/c)**		**2500 (c/c)**	
					**OR**	**p-value**	**OR**	**p-value**
2	*rs*113456069	113842451	C	T	11.58	7.871×10^{-11}	2.573	2.169×10^{-20}
2	*rs*112486568	113842455	G	A	11.58	7.871×10^{-11}	2.602	1.196×10^{-20}
6	*rs*146841607	29942575	A	G	11.66	2.119×10^{-07}	3.477	1.335×10^{-21}
11	*rs*17146528	64732006	A	G	2.161	6.762×10^{-06}	2.05	2.58×10^{-19}
11	*rs*113948263	64733224	T	C	2.161	6.762×10^{-06}	2.054	2.412×10^{-19}
15	*rs*289138	62599775	T	C	0.5053	1.694×10^{-10}	0.6688	1.872×10^{-17}
20	*rs*6115358	25856361	C	T	2.39	3.729×10^{-11}	1.856	8.445×10^{-28}
20	*rs*7343318	25856699	T	C	2.401	3.415×10^{-11}	1.864	3.712×10^{-28}

Table 11: GWAS summary statistics from GEMMA of the simulated risk SNPs in the homogeneous simulation of 500 and 2500 samples from a European reference population of 489 samples. $\mathbf{x(c/c)}$ refers to the $\mathbf{x}$ number of cases and controls.

GEMMA										
**Chr**	**rsID**	**Position**	**A1**	**A2**	**500 (c/c)**			**2500 (c/c)**		
					**OR(95% C.I.)**	**stderr**	**p-value**	**OR(95% C.I.)**	**stderr**	**p-value**
2	*rs*113456069	113842451	C	T	[1.447, 1.656]	0.0533	3.6277×10^{-15}	[1.204, 1.295]	0.0232	2.4661×10^{-21}
2	*rs*112486568	113842455	G	A	[1.447, 1.656]	0.0533	3.6277×10^{-15}	[1.207, 1.298]	0.0233	1.242×10^{-21}
6	*rs*146841607	29942575	A	G	[1.422, 1.685]	0.0672	1.8667×10^{-10}	[1.271, 1.379]	0.0277	1.165×10^{-23}
11	*rs*17146528	64732006	A	G	[1.124, 1.28]	0.0398	4.773×10^{-06}	[1.153, 1.227]	0.0189	1.0317×10^{-19}
11	*rs*113948263	64733224	T	C	[1.124, 1.28]	0.0398	4.773×10^{-06}	[1.153, 1.227]	0.0189	9.57×10^{-20}
15	*rs*289138	62599775	T	C	[0.8, 0.897]	0.0248	1.1861×10^{-10}	[0.883, 0.928]	0.0115	1.498×10^{-17}
20	*rs*6115358	25856361	C	T	[1.174, 1.294]	0.0307	3.0231×10^{-11}	[1.137, 1.19]	0.0135	5.006×10^{-28}
20	*rs*7343318	25856699	T	C	[1.175, 1.295]	0.0307	2.7338×10^{-11}	[1.138, 1.191]	0.0135	2.182×10^{-28}

Table 12: GWAS summary statistics from SNPTEST (Frequentist) of the simulated risk SNPs in the homogeneous simulation of 500 and 2500 samples from a European reference population of 489 samples. $\mathbf{x(c/c)}$ refers to the $\mathbf{x}$ number of cases and controls.

SNPTEST (Frequentist)										
**Chr**	**rsID**	**Position**	**A1**	**A2**	**500 (c/c)**			**2500 (c/c)**		
					**OR(95% C.I.)**	**stderr**	**p-value**	**OR(95% C.I.)**	**stderr**	**p-value**
2	*rs*113456069	113842451	C	T	-	-	-	[0.331, 0.49]	0.1022	3.65089×10^{-22}
2	*rs*112486568	113842455	G	A	-	-	-	-	-	-
6	*rs*146841607	29942575	A	G	-	-	-	-	-	-
11	*rs*17146528	64732006	A	G	[0.339, 0.657]	0.1718	3.80334×10^{-06}	[0.437, 0.591]	0.0799	4.32288×10^{-20}
11	*rs*113948263	64733224	T	C	[0.339, 0.657]	0.1718	3.80334×10^{-06}	[0.436, 0.591]	0.08	4.02425×10^{-20}
15	*rs*289138	62599775	T	C	[1.397, 1.989]	0.1068	9.21337×10^{-11}	[1.244, 1.455]	0.0473	5.99581×10^{-18}
20	*rs*6115358	25856361	C	T	[0.384, 0.613]	0.1343	2.70499×10^{-11}	[0.519, 0.639]	0.0574	6.63178×10^{-29}
20	*rs*7343318	25856699	T	C	[0.381, 0.61]	0.1346	2.5005×10^{-11}	[0.517, 0.637]	0.0573	2.71906×10^{-29}

Table 13: GWAS summary statistics from SNPTEST (Bayesian) of the simulated risk SNPs in the homogeneous simulation of 500 and 2500 samples from a European reference population of 489 samples. $\mathbf{x(c/c)}$ refers to the $\mathbf{x}$ number of cases and controls.

SNPTEST (Bayesian)										
**Chr**	**rsID**	**Position**	**A1**	**A2**	**500 (c/c)**			**2500 (c/c)**		
					**OR(95% C.I.)**	**stderr**	**ln(BF)**	**OR(95% C.I.)**	**stderr**	**ln(BF)**
2	*rs*113456069	113842451	C	T	-	-	-	[0.331, 0.49]	0.0888	16.1321
2	*rs*112486568	113842455	G	A	-	-	-	-	-	-
6	*rs*146841607	29942575	A	G	-	-	-	-	-	-
11	*rs*17146528	64732006	A	G	[0.339, 0.657]	0.1272	2.58078	[0.437, 0.591]	0.0735	15.4715
11	*rs*113948263	64733224	T	C	[0.339, 0.657]	0.1272	2.58078	[0.436, 0.591]	0.0736	15.4938
15	*rs*289138	62599775	T	C	[1.397, 1.989]	0.0926	6.89812	[1.244, 1.455]	0.046	14.7201
20	*rs*6115358	25856361	C	T	[0.384, 0.613]	0.1096	6.53781	[0.519, 0.639]	0.0549	24.5053
20	*rs*7343318	25856699	T	C	[0.381, 0.61]	0.1097	6.55545	[0.517, 0.637]	0.0549	24.8629

Table 14: GWAS summary statistics from EMMAX of the simulated risk SNPs in the homogeneous simulation of 500 and 2500 samples from an African reference population of 221 samples. $\mathbf{x(c/c)}$ refers to the $\mathbf{x}$ number of cases/controls.

EMMAX (African Reference Population)								
**Chr**	**rsID**	**Position**	**A1**	**A2**	**500 (c/c)**		**2500 (c/c)**	
					**OR**	**p-value**	**OR**	**p-value**
2	*rs*113456069	113842451	C	T	0.7188	3.24666×10^{-32}	0.814	1.25949×10^{-57}
2	*rs*112486568	113842455	G	A	0.7189	3.28526×10^{-32}	0.814	1.25949×10^{-57}
6	*rs*146841607	29942575	A	G	0.6198	4.23233×10^{-29}	0.7448	6.93739×10^{-48}
11	*rs*17146528	64732006	A	G	-	-	-	-
11	*rs*113948263	64733224	T	C	-	-	-	-
15	*rs*289138	62599775	T	C	1.1436	$1.85589\ \times10^{-07}$	0.9032	4.69036×10^{-18}
20	*rs*6115358	25856361	C	T	0.8713	$1.86356\ \times10^{-08}$	0.9178	8.26470×10^{-14}
20	*rs*7343318	25856699	T	C	0.8713	$1.86356\ \times10^{-08}$	0.9186	1.39689×10^{-13}

Table 15: GWAS summary statistics from GCTA of the simulated risk SNPs in the homogeneous simulation of 500 and 2500 samples from an African reference population of 221 samples. $\mathbf{x(c/c)}$ refers to the $\mathbf{x}$ number of cases/controls.

GCTA										
**Chr**	**rsID**	**Position**	**A1**	**A2**	**500 (c/c)**			**2500 (c/c)**		
					**OR(95% C.I.)**	**stderr**	**p-value**	**OR(95% C.I.)**	**stderr**	**p-value**
2	*rs*113456069	113842451	C	T	[1.334, 1.447]	0.02877	2.0873×10^{-30}	[1.204, 1.255]	0.01300	1.011×10^{-56}
2	*rs*112486568	113842455	G	A	[1.334, 1.447]	0.02877	2.0618×10^{-30}	[1.204, 1.255]	0.01300	1.011×10^{-56}
6	*rs*146841607	29942575	A	G	[1.518, 1.69]	0.0438	4.0449×10^{-27}	[1.301, 1.382]	0.02058	3.328×10^{-46}
11	*rs*17146528	64732006	A	G	[1.054, 1.263]	0.0531	0.00566	[1.097, 1.19]	0.02375	1.810×10^{-08}
11	*rs*113948263	64733224	T	C	[1.054, 1.263]	0.0531	0.00566	[1.096, 1.189]	0.02378	2.243×10^{-08}
15	*rs*289138	62599775	T	C	[0.825, 0.926]	0.0258	2.4723×10^{-07}	[1.085, 1.131]	0.01178	3.731×10^{-18}
20	*rs*6115358	25856361	C	T	[1.097, 1.193]	0.02449	3.02066×10^{-08}	[1.067, 1.112]	0.01150	1.046×10^{-13}
20	*rs*7343318	25856699	T	C	[1.097, 1.193]	0.02449	3.0206×10^{-08}	[1.066, 1.111]	0.01150	1.771×10^{-13}

Table 16: GWAS summary statistics from GCTA - LOCO of the simulated risk SNPs in the homogeneous simulation of 500 and 2500 samples from an African reference population of 221 samples. $\mathbf{x(c/c)}$ refers to the $\mathbf{x}$ number of cases/controls.

**GCTA - LOCO**										
**Chr**	**rsID**	**Position**	**A1**	**A2**	**500 (c/c)**			**2500 (c/c)**		
					**OR(95% C.I.)**	**stderr**	**p-value**	**OR(95% C.I.)**	**stderr**	**p-value**
2	*rs*113456069	113842451	C	T	[1.334, 1.447]	0.0288	1.97×10^{-30}	[1.204, 1.255]	0.0129	6.503×10^{-58}
2	*rs*112486568	113842455	G	A	[1.334, 1.447]	0.0288	1.95×10^{-30}	[1.204, 1.255]	0.0129	6.503×10^{-58}
6	*rs*146841607	29942575	A	G	[1.518, 1.689]	0.0437	2.963×10^{-27}	[1.304, 1.383]	0.0202	2.085×10^{-48}
11	*rs*17146528	64732006	A	G	[1.054, 1.263]	0.0531	0.00564	[1.097, 1.189]	0.0235	1.46×10^{-08}
11	*rs*113948263	64733224	T	C	[1.054, 1.263]	0.0531	0.00564	[1.096, 1.188]	0.0236	1.798×10^{-08}
15	*rs*289138	62599775	T	C	[0.825, 0.926]	0.0258	2.4438×10^{-07}	[1.085, 1.13]	0.0117	2.431×10^{-18}
20	*rs*6115358	25856361	C	T	[1.097, 1.193]	0.0245	3.0129×10^{-08}	[1.067, 1.111]	0.0113	4.080×10^{-14}
20	*rs*7343318	25856699	T	C	[1.097, 1.193]	0.0245	3.0129×10^{-08}	[1.066, 1.11]	0.0111	6.959×10^{-14}

Table 17: GWAS summary statistics from PLINK of the simulated risk SNPs in the homogeneous simulation of 500 and 2500 samples from an African reference population of 221 samples. $\mathbf{x(c/c)}$ refers to the $\mathbf{x}$ number of cases/controls.

**PLINK**								
**Chr**	**rsID**	**Position**	**A1**	**A2**	**500 (c/c)**		**2500 (c/c)**	
					**OR**	**p-value**	**OR**	**p-value**
2	*rs*113456069	113842451	C	T	3.103	3.476×10^{-25}	1.986	6.706×10^{-48}
2	*rs*112486568	113842455	G	A	3.115	2.987×10^{-25}	1.986	6.706×10^{-48}
6	*rs*146841607	29942575	A	G	11.18	2.225×10^{-25}	3.417	5.438×10^{-46}
11	*rs*17146528	64732006	A	G	1.77	0.007077	1.705	2.112×10^{-08}
11	*rs*113948263	64733224	T	C	1.77	0.007077	1.699	2.677×10^{-08}
15	*rs*289138	62599775	T	C	0.6694	7.744×10^{-06}	1.352	5.462×10^{-14}
20	*rs*6115358	25856361	C	T	1.596	2.94×10^{-07}	1.325	4.921×10^{-12}
20	*rs*7343318	25856699	T	C	1.596	2.94×10^{-07}	1.322	7.482×10^{-12}

Table 18: GWAS summary statistics from PLINK Logistic of the simulated risk SNPs in the homogeneous simulation of 500 and 2500 samples from an African reference population of 221 samples. $\mathbf{x(c/c)}$ refers to the $\mathbf{x}$ number of cases/controls.

**PLINK (Logistic)**								
**Chr**	**rsID**	**Position**	**A1**	**A2**	**500 (c/c)**		**2500 (c/c)**	
					**OR**	**p-value**	**OR**	**p-value**
2	*rs*113456069	113842451	C	T	4.271	2.459×10^{-28}	2.372	1.02×10^{-56}
2	*rs*112486568	113842455	G	A	4.270	2.488×10^{-28}	2.372	1.02×10^{-56}
6	*rs*146841607	29942575	A	G	13.4	3.916×10^{-19}	3.704	1.778×10^{-44}
11	*rs*17146528	64732006	A	G	1.827	0.006258	1.736	1.616×10^{-08}
11	*rs*113948263	64733224	T	C	1.827	0.006258	1.729	2.051×10^{-08}
15	*rs*289138	62599775	T	C	0.5762	2.861×10^{-07}	1.518	3.069×10^{-18}
20	*rs*6115358	25856361	C	T	1.763	3.203×10^{-08}	1.426	1.392×10^{-14}
20	*rs*7343318	25856699	T	C	1.763	3.203×10^{-08}	1.421	2.365×10^{-14}

Table 19: GWAS summary statistics from GEMMA of the simulated risk SNPs in the homogeneous simulation of 500 and 2500 samples from an African reference population of 221 samples. $\mathbf{x(c/c)}$ refers to the $\mathbf{x}$ number of cases/controls.

**GEMMA**										
**Chr**	**rsID**	**Position**	**A1**	**A2**	**500 (c/c)**			**2500 (c/c)**		
					**OR(95% C.I.)**	**stderr**	**p-value**	**OR(95% C.I.)**	**stderr**	**p-value**
2	*rs*113456069	113842451	C	T	[1.338, 1.444]	0.027	1.2738×10^{-28}	[1.207, 1.256]	0.0126	4.0675×10^{-57}
2	*rs*112486568	113842455	G	A	[1.338, 1.444]	0.027	1.2855×10^{-28}	[1.207, 1.256]	0.0126	4.0675×10^{-57}
6	*rs*146841607	29942575	A	G	[1.532, 1.694]	0.0414	3.513×10^{-26}	[1.308, 1.385]	0.0198	5.8603×10^{-48}
11	*rs*17146528	64732006	A	G	[1.054, 1.263]	0.0532	0.00581	[1.098, 1.19]	0.0235	1.1878×10^{-08}
11	*rs*113948263	64733224	T	C	[1.054, 1.263]	0.0532	0.00581	[1.097, 1.189]	0.0235	1.5194×10^{-08}
15	*rs*289138	62599775	T	C	[0.824, 0.925]	0.0255	2.4546×10^{-07}	[1.085, 1.131]	0.0116	2.369×10^{-18}
20	*rs*6115358	25856361	C	T	[1.1, 1.195]	0.0243	2.7626×10^{-08}	[1.070, 1.114]	0.0113	1.2400×10^{-14}
20	*rs*7343318	25856699	T	C	[1.1, 1.195]	0.0243	2.7626×10^{-08}	[1.069, 1.113]	0.0113	2.1165×10^{-14}

Table 20: GWAS summary statistics from SNPTEST (Frequentist) of the simulated risk SNPs in the homogeneous simulation of 500 and 2500 samples from an African reference population of 221 samples. $\mathbf{x(c/c)}$ refers to the $\mathbf{x}$ number of cases/controls.

**SNPTEST (Frequentist)**										
**Chr**	**rsID**	**Position**	**A1**	**A2**	**500 (c/c)**			**2500 (c/c)**		
					**OR(95% C.I.)**	**stderr**	**p-value**	**OR(95% C.I.)**	**stderr**	**p-value**
2	*rs*113456069	113842451	C	T	[0.257, 0.399]	0.1317	6.1438×10^{-32}	[0.459, 0.553]	0.0545	7.2405×10^{-60}
2	*rs*112486568	113842455	G	A	[0.256, 0.397]	0.1318	5.9524×10^{-32}	[0.459, 0.553]	0.0545	7.2405×10^{-60}
6	*rs*146841607	29942575	A	G	[0.046, 0.147]	0.2992	9.7078×10^{-32}	[0.245, 0.35]	0.0946	9.1593×10^{-51}
11	*rs*17146528	64732006	A	G	-	-	-	-	-	-
11	*rs*113948263	64733224	T	C	-	-	-	-	-	-
15	*rs*289138	62599775	T	C	[1.242, 1.767]	0.1074	2.2659×10^{-07}	[0.683, 0.799]	0.048	9.8459×10^{-19}
20	*rs*6115358	25856361	C	T	[0.527, 0.755]	0.1153	2.0967×10^{-06}	[0.697, 0.817]	0.0542	3.8735×10^{-11}
20	*rs*7343318	25856699	T	C	[0.527, 0.755]	0.1153	2.0967×10^{-06}	[0.698, 0.819]	0.0542	6.8517×10^{-11}

Table 21: GWAS summary statistics from SNPTEST (Bayesian) of the simulated risk SNPs in the homogeneous simulation of 500 and 2500 samples from an African reference population of 221 samples. $\mathbf{x(c/c)}$ refers to the $\mathbf{x}$ number of cases/controls.

**SNPTEST (Bayesian)**										
**Chr**	**rsID**	**Position**	**A1**	**A2**	**500 (c/c)**			**2500 (c/c)**		
					**OR(95% C.I.)**	**stderr**	**ln(BF)**	**OR(95% C.I.)**	**stderr**	**ln(BF)**
2	*rs*113456069	113842451	C	T	[0.257, 0.399]	0.1057	21.7211	[0.459, 0.553]	0.0523	53.508
2	*rs*112486568	113842455	G	A	[0.256, 0.397]	0.1057	21.7256	[0.459, 0.553]	0.0523	53.508
6	*rs*146841607	29942575	A	G	[0.046, 0.147]	0.1399	14.7896	[0.245, 0.35]	0.0825	40.6405
11	*rs*17146528	64732006	A	G	-	-	-	-	-	-
11	*rs*113948263	64733224	T	C	-	-	-	-	-	-
15	*rs*289138	62599775	T	C	[1.242, 1.767]	0.0936	4.25122	[0.683, 0.799]	0.0466	15.4393
20	*rs*6115358	25856361	C	T	[0.527, 0.755]	0.099	3.40901	[0.697, 0.817]	0.0523	8.269
20	*rs*7343318	25856699	T	C	[0.527, 0.755]	0.099	3.40901	[0.698, 0.819]	0.0522	8.04308

0.2.2 Assessment of the Admixture Simulation GWAS Analysis

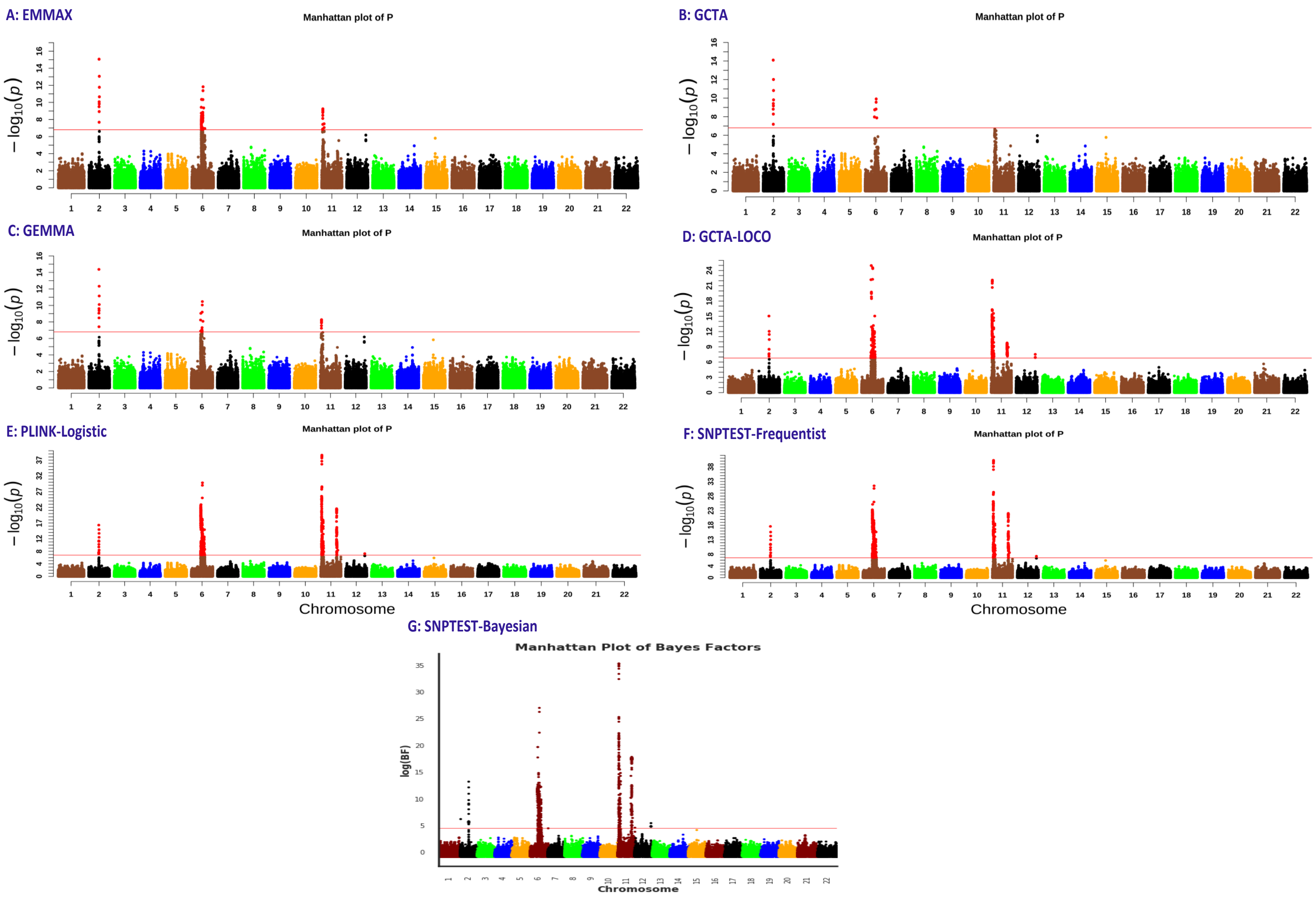

Figure 12: The Manhattan plots of GWAS of the simulated 3-way admixed population using EMMAX, GEMMA, PLINK, GCTA and SNPTEST tools. The red line represent the GWAS significance threshold line, and the significant SNPs are shown in red.

Table 22: GWAS statistics from EMMAX of the simulated risk SNPs in the 3-way admixture simulation for 2500 cases and 2500 controls.

**EMMAX**						
**Chr**	**rsID**	**Position**	**A1**	**A2**	**OR**	**p-value**
2	*rs*76091761	119924776	C	T	0.8284	8.70874 $\times10^{-16}$
6	*rs*79354975	78841154	T	C	0.9074	4.67783 $\times10^{-11}$
11	*rs*73417185	4119431	A	G	0.9452	6.73071$\times10^{-04}$
15	*rs*2960806	60169020	G	A	1.0497	1.54807 $\times10^{-06}$

Table 23: GWAS statistics from GEMMA of the simulated risk SNPs in the 3-way admixture simulation for 2500 cases and 2500 controls.

**GEMMA**							
**Chr**	**rsID**	**Position**	**A1**	**A2**	**OR(95% C.I.)**	**stderr**	**p-value**
2	*rs*76091761	119924776	C	T	[1.1627, 1.2533]	0.02312	4.31377 $\times10^{-15}$
6	*rs*79354975	78841154	T	C	[1.0722, 1.1297]	0.01467	6.39394 $\times10^{-10}$
11	*rs*73417185	4119431	A	G	[1.0232, 1.0879]	0.0165	1.51937 $\times10^{-03}$
15	*rs*2960806	60169020	G	A	[0.9329, 0.9724]	0.01009	1.46203 $\times10^{-06}$

Table 24: GWAS statistics from GCTA of the simulated risk SNPs in the 3-way admixture simulation for 2500 cases and 2500 controls.

**GCTA**							
**Chr**	**rsID**	**Position**	**A1**	**A2**	**OR(95% C.I.)**	**stderr**	**p-value**
2	*rs*76091761	119924776	C	T	[1.1573, 1.2511]	0.02392	7.99158 $\times10^{-15}$
6	*rs*79354975	78841154	T	C	[1.0651, 1.1236]	0.01492	1.52098 $\times10^{-09}$
11	*rs*73417185	4119431	A	G	[1.0182, 1.082]	0.01628	2.68599$\times10^{-03}$
15	*rs*2960806	60169020	G	A	[0.9329, 0.9726]	0.01012	1.69894 $\times10^{-06}$

Table 25: GWAS statistics from GCTA-LOCO of the simulated risk SNPs in the 3-way admixture simulation for 2500 cases and 2500 controls.

**GCTA-LOCO**							
**Chr**	**rsID**	**Position**	**A1**	**A2**	**OR(95% C.I.)**	**stderr**	**p-value**
2	*rs*76091761	119924776	C	T	[1.1378, 1.2175]	0.02034	8.93609 $\times 10^{-16}$
6	*rs*79354975	78841154	T	C	[1.0972, 1.142]	0.01144	5.40953 $\times 10^{-23}$
11	*rs*73417185	4119431	A	G	[1.0656, 1.111]	0.01158	2.66512 $\times 10^{-13}$
15	*rs*2960806	60169020	G	A	[0.9488, 0.9844]	0.00908	0.00018

Table 26: GWAS statistics from PLINK (Logistic) of the simulated risk SNPs in the 3-way admixture simulation for 2500 cases and 2500 controls.

**PLINK (Logistic)**						
**Chr**	**rsID**	**Position**	**A1**	**A2**	**OR**	**p-value**
2	*rs*76091761	119924776	C	T	2.263	4.564 $\times 10^{-17}$
6	*rs*79354975	78841154	T	C	1.835	1.022 $\times 10^{-25}$
11	*rs*73417185	4119431	A	G	1.711	3.868 $\times 10^{-19}$
15	*rs*2960806	60169020	G	A	0.8226	1.249 $\times 10^{-06}$

Table 27: GWAS statistics from SNPTEST of the simulated risk SNPs in the 3-way admixture simulation for 2500 cases and 2500 controls.

**SNPTEST - Frequentist**							
**Chr**	**rsID**	**Position**	**A1**	**A2**	**OR(95% C.I.)**	**stderr**	**p-value**
2	*rs*76091761	119924776	C	T	[0.2517, 0.6329]	0.09724	2.49893 $\times 10^{-18}$
6	*rs*79354975	78841154	T	C	[0.43, 0.6571]	0.05793	1.0629 $\times 10^{-26}$
11	*rs*73417185	4119431	A	G	[0.4674, 0.7028]	0.06005	1.47774 $\times 10^{-19}$
15	*rs*2960806	60169020	G	A	[1.1367, 1.2946]	0.04028	1.18143 $\times 10^{-06}$

Table 28: GWAS statistics from SNPTEST of the simulated risk SNPs in the 3-way admixture simulation for 2500 cases and 2500 controls.

**SNPTEST - Bayesian**							
**Chr**	**rsID**	**Position**	**A1**	**A2**	**OR(95% C.I.)**	**stderr**	**log(BF)**
2	*rs*76091761	119924776	C	T	[0.3474, 0.683]	0.08562	13.2702
6	*rs*79354975	78841154	T	C	[0.4611, 0.6782]	0.05539	22.4432
11	*rs*73417185	4119431	A	G	[0.4992, 0.7238]	0.0573	15.815
15	*rs*2960806	60169020	G	A	[1.129, 1.2838]	0.03947	4.22825

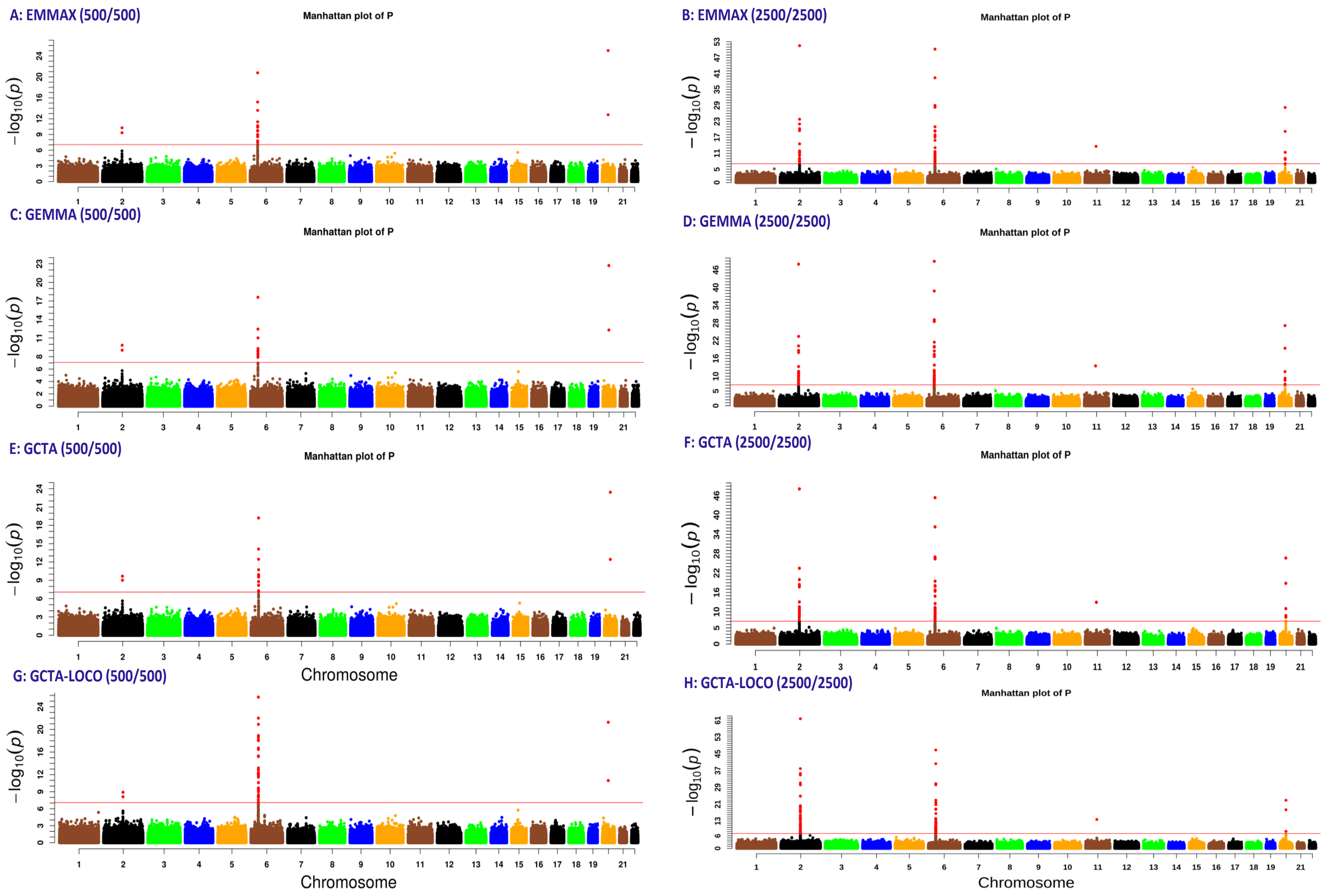

Figure 13: The Manhattan plots of GWAS of the simulated 5-way admixed population using EMMAX, GEMMA, and GCTA. Column A-C-E-G correspond to the 500 cases and 500 controls simulation, while column B-D-F-H correspond to 2500 cases and 2500 controls simulation. The significance threshold line and the significant SNPs are shown in red.

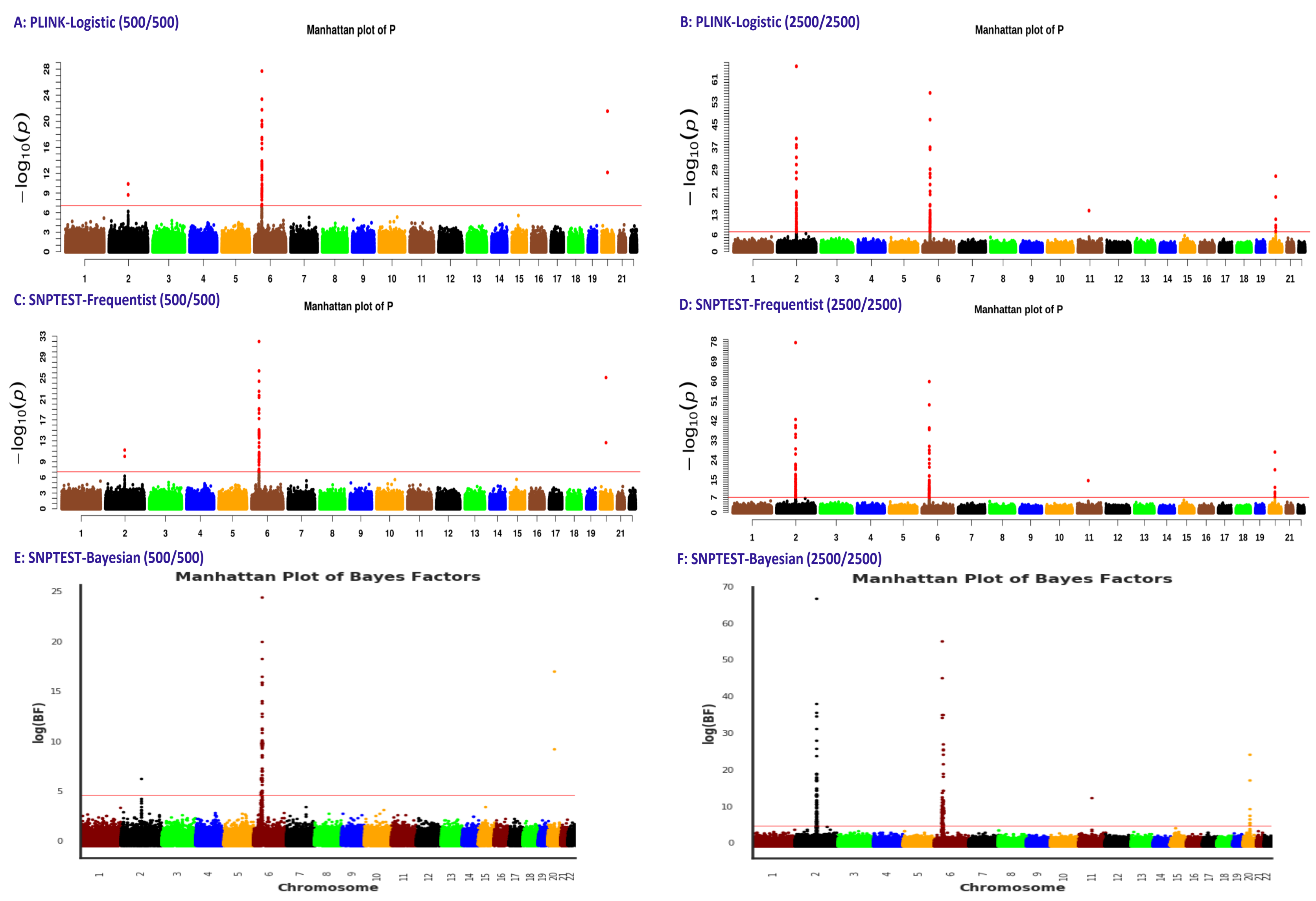

Figure 14: The Manhattan plots of GWAS of the simulated 5-way admixed population using PLINK and SNPTEST. Column A-C-E correspond to the 500 cases and 500 controls simulation, while column B-D-F correspond to 2500 cases and 2500 controls simulation. The significance threshold line and the significant SNPs are shown in red.

Table 29: GWAS statistics from EMMAX of the simulated risk SNPs in the 5-way admixture simulation for the 500 cases and 500 controls (500 (c/c)) and 2500 cases and the 2500 controls (2500 (c/c)) sample sizes.

EMMAX								
**Chr**	**rsID**	**Position**	**A1**	**A2**	**500 (c/c)**		**2500 (c/c)**	
					**OR**	**p-value**	**OR**	**p-value**
2	*rs*13410964	113843283	G	A	0.8991	1.25749 $\times 10^{-05}$	0.8945	1.55707 $\times 10^{-24}$
2	*rs*17042838	113843337	G	A	0.7824	5.29589 $\times 10^{-11}$	0.7578	3.23287 $\times 10^{-52}$
6	*rs*2232238	29942857	T	C	0.776	1.65525 $\times 10^{-21}$	0.8357	6.30461 $\times 10^{-51}$
11	*rs*7106136	64748278	G	T	0.9308	0.00207	0.9564	2.75317 $\times 10^{-05}$
11	*rs*10897540	64757496	C	T	0.9216	0.00106	0.916	2.34892 $\times 10^{-14}$
15	*rs*11853943	48779402	C	T	0.9161	0.0005	0.9613	0.00044
20	*rs*6115375	25871801	A	C	0.7415	9.36899 $\times 10^{-26}$	0.8584	5.57954 $\times 10^{-29}$
20	*rs*6107104	25922993	G	A	0.8463	1.69613 $\times 10^{-13}$	0.9118	5.73349 $\times 10^{-20}$

Table 30: GWAS statistics from GEMMA of the simulated risk SNPs in the 5-way admixture simulation for the 500 cases and 500 controls (500 (c/c)) and 2500 cases and the 2500 controls (2500 (c/c)) sample sizes.

					**GEMMA**					
**Chr**	**rsID**	**Position**	**A1**	**A2**	**500 (c/c)**			**2500 (c/c)**		
					**OR(95% C.I.)**	**stderr**	**p-value**	**OR(95% C.I.)**	**stderr**	**p-value**
2	*rs*13410964	113843283	G	A	[1.0659, 1.1603]	0.02409	1.52552 $\times10^{-05}$	[1.0979, 1.1402]	0.01079	3.30181 $\times10^{-24}$
2	*rs*17042838	113843337	G	A	[1.2049, 1.3497]	0.03694	1.43616 $\times10^{-10}$	[1.2953, 1.3645]	0.01768	1.15543 $\times10^{-48}$
6	*rs*2232238	29942857	T	C	[1.2466, 1.3435]	0.0247	2.56559 $\times10^{-18}$	[1.1713, 1.2125]	0.01053	1.26978 $\times10^{-49}$
11	*rs*7106136	64748278	G	T	[1.0278, 1.119]	0.02325	2.26595 $\times10^{-03}$	[1.0245, 1.0661]	0.01063	2.85096 $\times10^{-05}$
11	*rs*10897540	64757496	C	T	[1.0369, 1.1344]	0.02489	8.56753 $\times10^{-04}$	[1.0695, 1.1143]	0.01144	3.17538 $\times10^{-14}$
15	*rs*11853943	48779402	C	T	[1.0415, 1.1406]	0.02529	4.81497 $\times10^{-04}$	[1.0184, 1.062]	0.01114	4.06325 $\times10^{-04}$
20	*rs*6115375	25871801	A	C	[1.2982, 1.4079]	0.02797	1.93975 $\times10^{-23}$	[1.1397, 1.1933]	0.01367	7.21483 $\times10^{-28}$
20	*rs*6107104	25922993	G	A	[1.1383, 1.2257]	0.02229	5.0082 $\times10^{-13}$	[1.0772, 1.1165]	0.01002	3.4046 $\times10^{-20}$

Table 31: GWAS statistics from GCTA of the simulated risk SNPs in the 5-way admixture simulation for the 500 cases and 500 controls (500 (c/c)) and 2500 cases and the 2500 controls (2500 (c/c)) sample sizes.

GCTA										
**Chr**	**rsID**	**Position**	**A1**	**A2**	**500 (c/c)**			**2500 (c/c)**		
					**OR(95% C.I.)**	**stderr**	**p-value**	**OR(95% C.I.)**	**stderr**	**p-value**
2	*rs*13410964	113843283	G	A	[1.0611, 1.1569]	0.02442	2.2691 $\times 10^{-05}$	[1.0965, 1.1396]	0.01099	3.17726 $\times 10^{-24}$
2	*rs*17042838	113843337	G	A	[1.1968, 1.3447]	0.03772	2.11839 $\times 10^{-10}$	[1.2806, 1.3541]	0.01877	7.82272 $\times 10^{-49}$
6	*rs*2232238	29942857	T	C	[1.2272, 1.3332]	0.02702	6.18364 $\times 10^{-20}$	[1.1584, 1.2041]	0.01168	3.93074 $\times 10^{-46}$
11	*rs*7106136	64748278	G	T	[1.026, 1.116]	0.02296	0.00283	[1.0232, 1.0646]	0.01056	4.73877 $\times 10^{-05}$
11	*rs*10897540	64757496	C	T	[1.0332, 1.1289]	0.02442	0.00141	[1.0658, 1.1105]	0.01138	1.1595 $\times 10^{-13}$
15	*rs*11853943	48779402	C	T	[1.037, 1.1349]	0.02497	0.00096	[1.0156, 1.0572]	0.01062	0.00075
20	*rs*6115375	25871801	A	C	[1.2863, 1.4005]	0.02912	3.77319 $\times 10^{-24}$	[1.131, 1.1839]	0.01349	2.16219 $\times 10^{-27}$
20	*rs*6107104	25922993	G	A	[1.1361, 1.2259]	0.02292	3.88002 $\times 10^{-13}$	[1.0763, 1.1162]	0.01016	1.47909 $\times 10^{-19}$

Table 32: GWAS statistics from GCTA-LOCO of the simulated risk SNPs in the 5-way admixture simulation for the 500 cases and 500 controls (500 (c/c)) and 2500 cases and the 2500 controls (2500 (c/c)) sample sizes.

GCTA-LOCO										
**Chr**	**rsID**	**Position**	**A1**	**A2**	**500 (c/c)**			**2500 (c/c)**		
					**OR(95% C.I.)**	**stderr**	**p-value**	**OR(95% C.I.)**	**stderr**	**p-value**
2	*rs*13410964	113843283	G	A	[1.0672, 1.1595]	0.02355	5.11541 $\times 10^{-06}$	[1.1061, 1.1457]	0.01009	6.95639 $\times 10^{-32}$
2	*rs*17042838	113843337	G	A	[1.161, 1.2931]	0.0337	1.27083 $\times 10^{-09}$	[1.2648, 1.3256]	0.01551	2.0582 $\times 10^{-62}$
6	*rs*2232238	29942857	T	C	[1.2207, 1.3069]	0.022	1.95672 $\times 10^{-26}$	[1.1319, 1.1699]	0.0097	1.49418 $\times 10^{-47}$
11	*rs*7106136	64748278	G	T	[1.0269, 1.111]	0.02145	0.00188	[1.0252, 1.064]	0.0099	1.05493 $\times 10^{-05}$
11	*rs*10897540	64757496	C	T	[1.0265, 1.1162]	0.0229	0.00261	[1.0647, 1.1066]	0.01071	1.67974 $\times 10^{-14}$
15	*rs*11853943	48779402	C	T	[1.0259, 1.1169]	0.02323	0.003	[1.0094, 1.0496]	0.01025	0.00456
20	*rs*6115375	25871801	A	C	[1.2513, 1.3597]	0.02765	5.40007 $\times 10^{-22}$	[1.1083, 1.1571]	0.01243	1.22554 $\times 10^{-23}$
20	*rs*6107104	25922993	G	A	[1.1174, 1.2032]	0.02187	1.0686 $\times 10^{-11}$	[1.0718, 1.11]	0.00974	4.1109 $\times 10^{-19}$

Table 33: GWAS statistics from PLINK (Logistic) of the simulated risk SNPs in the 5-way admixture simulation for the 500 cases and 500 controls (500 (c/c)) and 2500 cases and the 2500 controls (2500 (c/c)) sample sizes.

PLINK (Logistic)								
**Chr**	**rsID**	**Position**	**A1**	**A2**	**500 (c/c)**		**2500 (c/c)**	
					**OR**	**p-value**	**OR**	**p-value**
2	*rs*13410964	113843283	G	A	1.651	6.591 $\times10^{-07}$	1.69	3.833 $\times10^{-34}$
2	*rs*17042838	113843337	G	A	2.949	4.087 $\times10^{-11}$	4.054	2.047 $\times10^{-66}$
6	*rs*2232238	29942857	T	C	3.335	2.023 $\times10^{-28}$	2.08	5.581 $\times10^{-57}$
11	*rs*7106136	64748278	G	T	1.338	0.00205	1.213	6.187 $\times10^{-06}$
11	*rs*10897540	64757496	C	T	1.416	0.00065	1.449	2.694 $\times10^{-15}$
15	*rs*11853943	48779402	C	T	1.442	0.00049	1.172	0.00041
20	*rs*6115375	25871801	A	C	3.849	2.811 $\times10^{-22}$	1.849	1.772 $\times10^{-27}$
20	*rs*6107104	25922993	G	A	2.009	6.975 $\times10^{-13}$	1.457	3.907 $\times10^{-20}$

Table 34: GWAS statistics from SNPTEST of the simulated risk SNPs in the 5-way admixture simulation for the 500 cases and 500 controls (500 (c/c)) and 2500 cases and the 2500 controls (2500 (c/c)) sample sizes.

**SNPTEST - Frequentist**										
**Chr**	**rsID**	**Position**	**A1**	**A2**	**500 (c/c)**			**2500 (c/c)**		
					**OR(95% C.I.)**	**stderr**	**p-value**	**OR(95% C.I.)**	**stderr**	**p-value**
2	*rs*13410964	113843283	G	A	[0.4118, 0.8067]	0.10075	6.00277×10^{-07}	[0.508, 0.6769]	0.04308	4.25803×10^{-35}
2	*rs*17042838	113843337	G	A	[0.0206, 0.6624]	0.16372	6.01465×10^{-12}	[0.0875, 0.4062]	0.0813	2.76036×10^{-78}
6	*rs*2232238	29942857	T	C	[0.087, 0.5135]	0.10881	1.04881×10^{-32}	[0.3903, 0.5708]	0.04605	1.58376×10^{-60}
11	*rs*7106136	64748278	G	T	[0.5693, 0.9391]	0.09433	0.00267	[0.7403, 0.9077]	0.0427	5.58253×10^{-06}
11	*rs*10897540	64757496	C	T	[0.5123, 0.9123]	0.10204	0.00082	[0.5991, 0.7831]	0.04692	2.16326×10^{-15}
15	*rs*11853943	48779402	C	T	[0.4931, 0.9049]	0.10505	0.00061	[0.7644, 0.9401]	0.0448	0.00035
20	*rs*6115375	25871801	A	C	[-0.0105, 0.5334]	0.13876	8.36717×10^{-26}	[0.4309, 0.6527]	0.05657	2.11273×10^{-28}
20	*rs*6107104	25922993	G	A	[0.3106, 0.6912]	0.0971	2.37625×10^{-13}	[0.6068, 0.7674]	0.04097	2.44542×10^{-20}

Table 35: GWAS statistics from SNPTEST of the simulated risk SNPs in the 5-way admixture simulation for the 500 cases and 500 controls (500 (c/c)) and 2500 cases and the 2500 controls (2500 (c/c)) sample sizes.

**SNPTEST - Bayesian**										
**Chr**	**rsID**	**Position**	**A1**	**A2**	**500 (c/c)**			**2500 (c/c)**		
					**OR(95% C.I.)**	**stderr**	**log(BF)**	**OR(95% C.I.)**	**stderr**	**log(BF)**
2	*rs*13410964	113843283	G	A	[0.4981, 0.8478]	0.08923	4.01393	[0.524, 0.6886]	0.042	31.1146
2	*rs*17042838	113843337	G	A	[0.2777, 0.7571]	0.12231	6.27021	[0.1564, 0.4418]	0.0728	66.6651
6	*rs*2232238	29942857	T	C	[0.2111, 0.5701]	0.09159	24.4739	[0.411, 0.586]	0.04463	55.13
11	*rs*7106136	64748278	G	T	[0.627, 0.9606]	0.08512	1.24261	[0.7492, 0.9128]	0.04175	3.60867
11	*rs*10897540	64757496	C	T	[0.5863, 0.9413]	0.09056	1.60045	[0.6151, 0.794]	0.04562	12.3313
15	*rs*11853943	48779402	C	T	[0.5734, 0.9367]	0.09267	1.68192	[0.7731, 0.9444]	0.0437	1.98332
20	*rs*6115375	25871801	A	C	[0.1833, 0.6061]	0.10785	17.051	[0.4607, 0.673]	0.05416	24.1036
20	*rs*6107104	25922993	G	A	[0.4017, 0.7389]	0.08601	9.23123	[0.619, 0.7761]	0.04008	17.1318

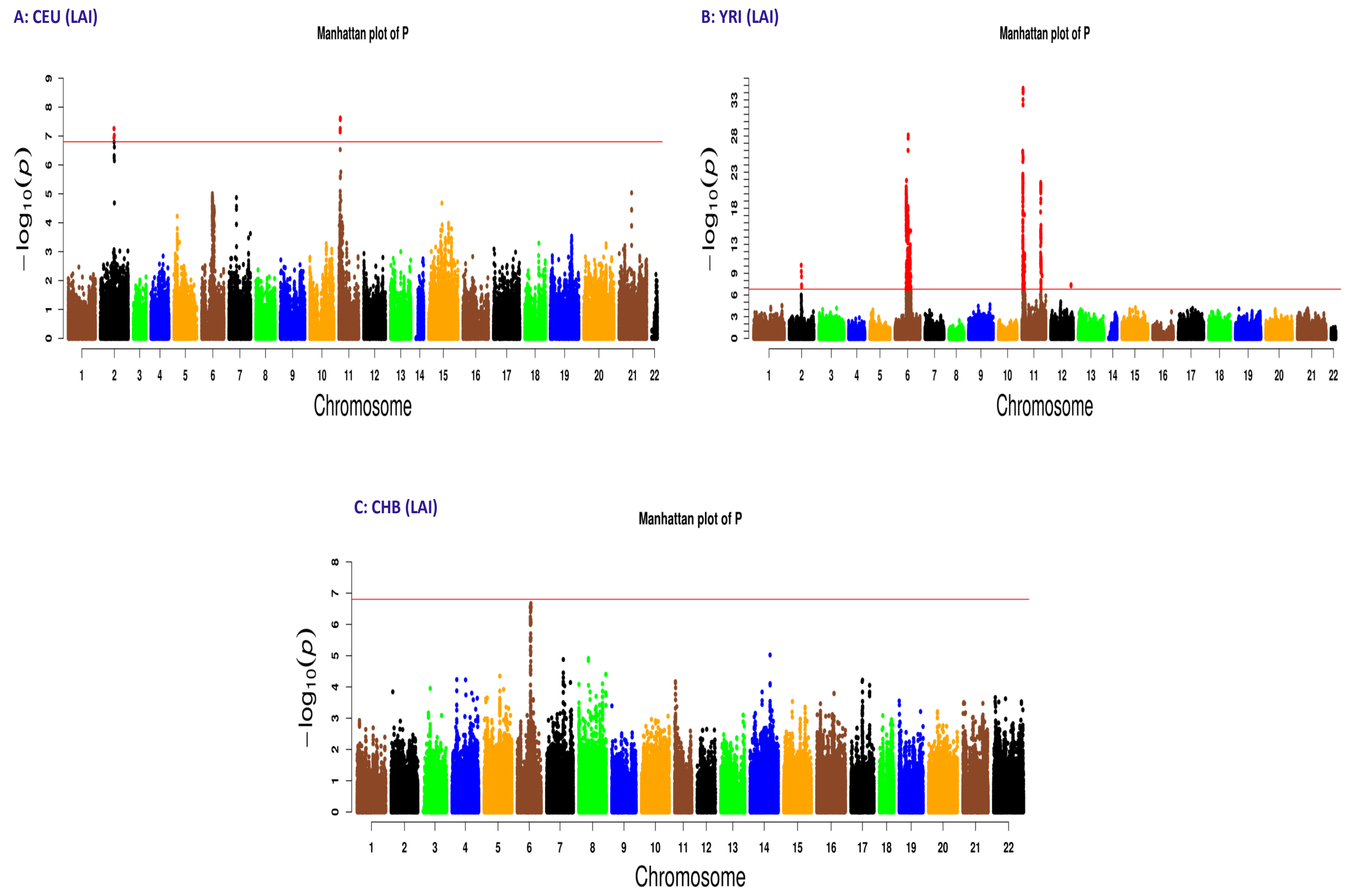

Figure 15: The Manhattan plots of GWAS of the simulated 3-way admixed population using TRACTOR corresponding to A) CEU, B) YRI and C) CHB ancestral populations. The red line represent the GWAS significance threshold line, and the significant SNPs are shown in red.

Table 36: Joint association summary statistics from TRACTOR of the simulated risk SNPs in the 3-way admixture simulation for 2500 cases and 2500 controls, for joint association of each of the parental populations.

**TRACTOR**										
					**CEU**		**YRI**		**CHB**	
**Chr**	**rsID**	**Position**	**A1**	**A2**	**OR**	**p-value**	**OR**	**p-value**	**OR**	**p-value**
2	*rs*76091761	119924776	C	T	0.4045	5.5393 $\times 10^{-08}$	0.4564	7.70116 $\times 10^{-11}$	73289	0.95465
6	*rs*79354975	78841154	T	C	0.5664	0.17695	0.5206	9.71775 $\times 10^{-27}$	0.7256	0.1448
11	*rs*73417185	4119431	A	G	0.7201	0.01217	0.5631	3.46656 $\times 10^{-18}$	0.6586	0.46555
15	*rs*296080	60169020	G	A	1.369	2.09975 $\times 10^{-05}$	1.1681	0.00156	0.7727	0.29236

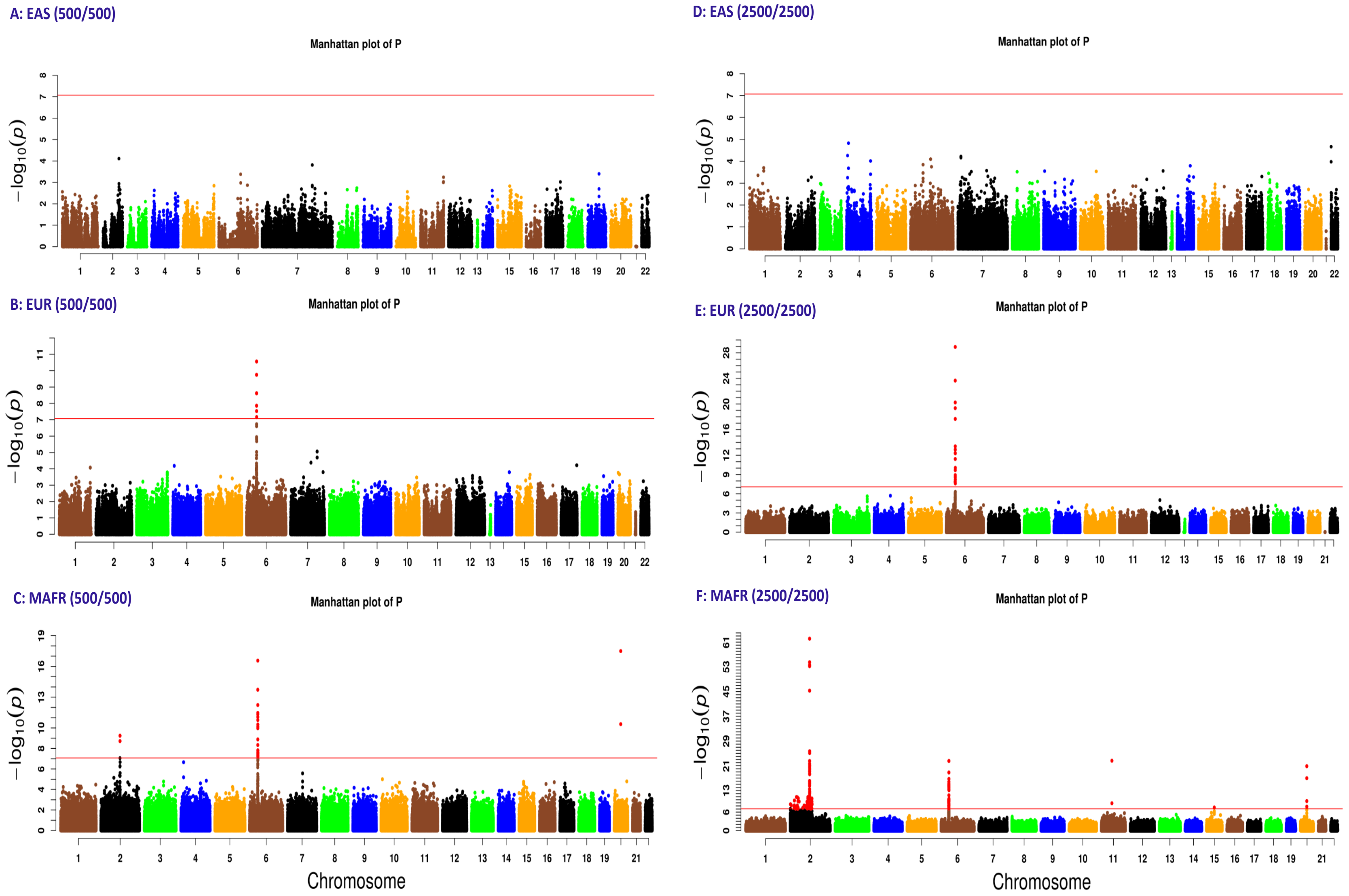

Figure 16: The Manhattan plots of joint association of the simulated 5-way admixed population using TRACTOR with A-C corresponding to the 500 cases and 500 controls 5-way admixture simulation, while D-F corresponds to the 2500 cases and 2500 controls 5-way admixture simulation for EAS, EUR and MAFR ancestral populations. The red line represent the GWAS significance threshold line, and the significant SNPs are shown in red.

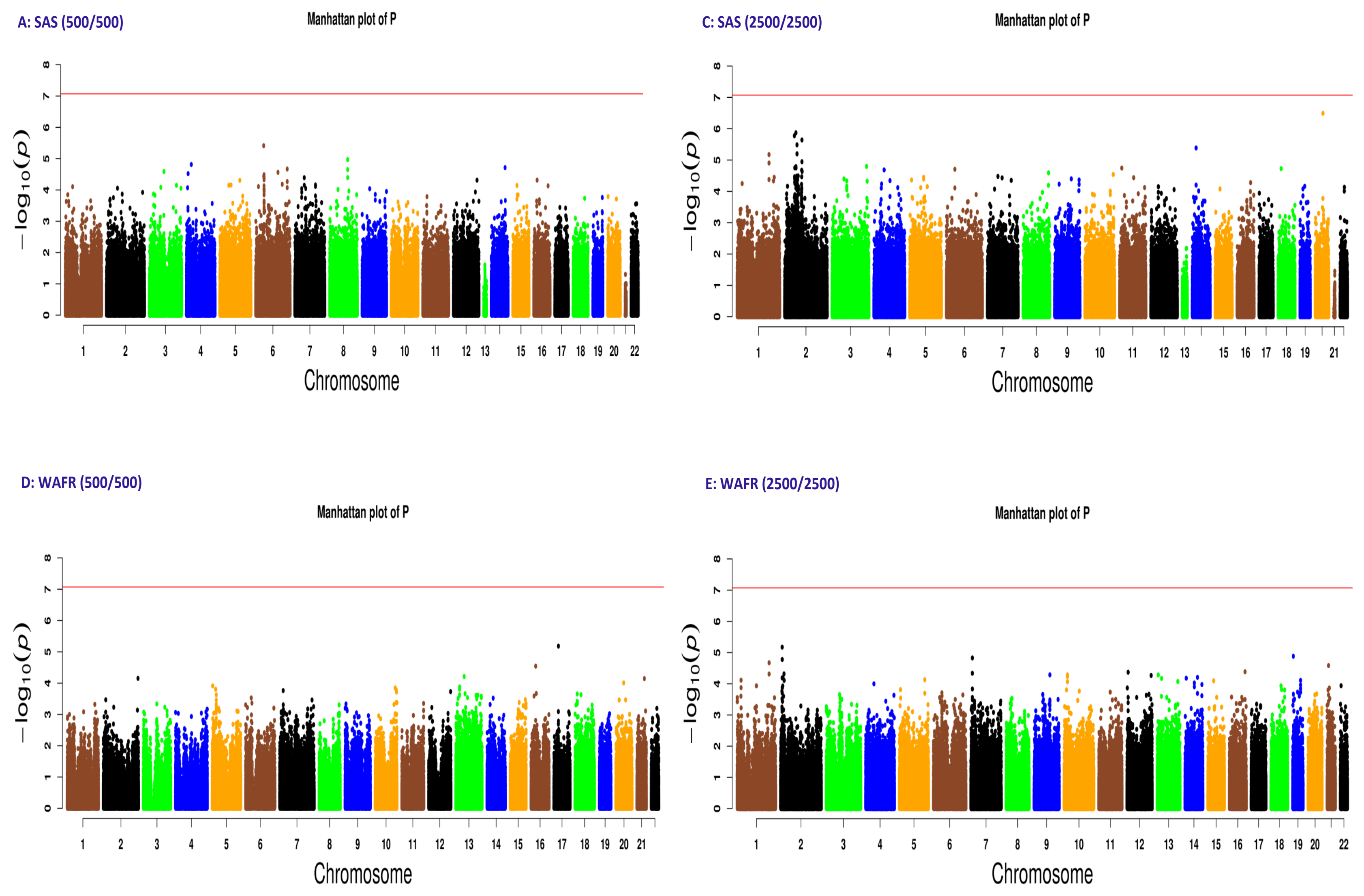

Figure 17: The Manhattan plots of the joint association of the simulated 5-way admixed population using TRACTOR with A-B corresponding to the 500 cases and 500 controls 5-way admixture simulation, while C-D corresponds to the 2500 cases and 2500 controls 5-way admixture simulation for SAS and WAFR ancestral populations. The red line represent the GWAS significance threshold line, and the significant SNPs are shown in red.

Table 37: Association statistics from TRACTOR of the simulated risk SNPs in the 5-way admixture simulation for the 500 cases and 500 controls corresponding to the joint association with EAS, EUR, MAFR, SAS and WAFR ancestral populations. TRACTOR did not generate results for most of the SNPs, and this has been indicated by "-".

**TRACTOR**														
					**EAS**		**EUR**		**MAFR**		**SAS**		**WAFR**	
**Chr**	**rsID**	**Position**	**A1**	**A2**	**OR**	**p-value**	**OR**	**p-value**	**OR**	**p-value**	**OR**	**p-value**	**OR**	**p-value**
2	rs13410964	113843283	G	A	-	-	426208.0985	0.96816	0.6023	8.6422 $\times 10^{-07}$	0.252	0.04373	2.4466	0.45348
2	rs17042838	113843337	G	A	-	-	0.4032	0.49828	0.3475	4.3531 $\times 10^{-10}$	0.1676	0.02638	-	-
6	rs2232238	29942857	T	C	0.0	0.98518	0.3198	3.33735 $\times 10^{-12}$	0.2278	1.02741 $\times 10^{-18}$	0.5682	0.03592	0.0	0.9786
11	rs10897540	64757496	C	T	-	-	-	-	-	-	-	-	-	-
11	rs7106136	64748278	G	T	-	-	-	-	0.7477	0.0021	0.0	0.97861	-	-
15	rs11853943	48779402	C	T	-	-	-	-	0.6665	0.00111	0.775	0.17258	-	-
20	rs6115375	25871801	A	C	-	-	-	-	0.2488	2.54561 $\times 10^{-19}$	-	-	0.3165	9.85935 $\times 10^{-05}$
20	rs6107104	25922993	G	A	-	-	-	-	0.4871	3.19736 $\times 10^{-11}$	-	-	0.5499	0.00648

Table 38: Association statistics from TRACTOR of the simulated risk SNPs in the 5-way admixture simulation for the 2500 cases and 2500 controls corresponding to the joint association with EAS, EUR, MAFR, SAS and WAFR ancestral populations. TRACTOR did not generate results for most of the SNPs, and this has been indicated by "-".

TRACTOR														
					**EAS**		**EUR**		**MAFR**		**SAS**		**WAFR**	
**Chr**	**rsID**	**Position**	**A1**	**A2**	**OR**	**p-value**	**OR**	**p-value**	**OR**	**p-value**	**OR**	**p-value**	**OR**	**p-value**
2	rs13410964	113843283	G	A	-	-	1.6501	0.0953	1.2927	4.60272 $\times 10^{-08}$	1.5227	0.02882	0.4684	0.25958
2	rs17042838	113843337	G	A	-	-	0.2018	0.00199	0.3492	2.66103 $\times 10^{-62}$	0.3985	0.00083	0.2512	0.12316
6	rs2232238	29942857	T	C	0.382	0.01196	0.4321	2.56822 $\times 10^{-33}$	0.4234	6.44012 $\times 10^{-28}$	0.6992	1.975 $\times 10^{-05}$	0.1547	0.0002
11	rs10897540	64757496	C	T	-	-	524770.8657	0.95193	0.6843	8.15043 $\times 10^{-16}$	1.9015	0.39792	-	-
11	rs7106136	64748278	G	T	-	-	555662.2919	0.95376	0.8198	3.46212 $\times 10^{-06}$	3.2144	0.12739	-	-
15	rs11853943	48779402	C	T	-	-	-	-	0.8267	0.00125	0.8846	0.07095	0.3202	0.36222
20	rs6115375	25871801	A	C	-	-	-	-	0.5461	3.24976 $\times 10^{-25}$	0.2021	0.18699	0.4459	0.00023
20	rs6107104	25922993	G	A	-	-	-	-	0.6948	9.98254 $\times 10^{-18}$	0.0229	0.01392	0.6009	0.00127

Table 39: The list of risk SNPs simulated under the 3-way admixture scenario, with the correponding most informative ancestry (**MIA**) selected, the odds ratio and p-value under the admixture mapping (**MIA OR** and **MIA Pval**, respectively) the genotype association odds ratio and p-value (**Genotype OR** and **Genotype Pval**, respectively) and the PPA for the joint association (**Joint PPA**) using the RFMIX inferences (**RFMIX**) and the true local ancestry (**TRUE**).

**rsID**	**CHR**	**Position**	**RFMIX**					
			**MIA**	**MIA OR**	**MIA Pval**	**Genotype OR**	**Genotype Pval**	**Joint PPA**
*rs*76091761	2	119924776	CEU	0.9862	2.07860 $\times 10^{-03}$	1.0188	4.69869 $\times 10^{-15}$	0.9999
*rs*79354975	6	78841154	YRI	1.0140	1.32868 $\times 10^{-03}$	1.0313	1.82564 $\times 10^{-10}$	0.9999
*rs*734171851	11	4119431	YRI	0.9694	6.17746 $\times 10^{-06}$	1.0213	7.54124 $\times 10^{-05}$	0.9999
*rs*2960806	15	60169020	CHB	0.9920	3.81523 $\times 10^{-03}$	1.0245	1.68258 $\times 10^{-06}$	0.9997

**rsID**	**CHR**	**Position**	**TRUE**					
			**MIA**	**MIA OR**	**MIA Pval**	**Genotype OR**	**Genotype Pval**	**Joint PPA**
*rs*76091761	2	119924776	YRI	1.0454	1.63152 $\times 10^{-25}$	1.0188	4.69869 $\times 10^{-15}$	0.9999
*rs*79354975	6	78841154	CEU	1.1404	1.73069 $\times 10^{-100}$	1.0313	1.82564 $\times 10^{-10}$	0.9999
*rs*734171851	11	4119431	CEU	1.1449	2.22346 $\times 10^{-101}$	1.0213	7.54124 $\times 10^{-05}$	0.9999
*rs*2960806	15	60169020	CHB	0.9687	7.98378 $\times 10^{-10}$	1.0245	1.68258 $\times 10^{-06}$	0.9999

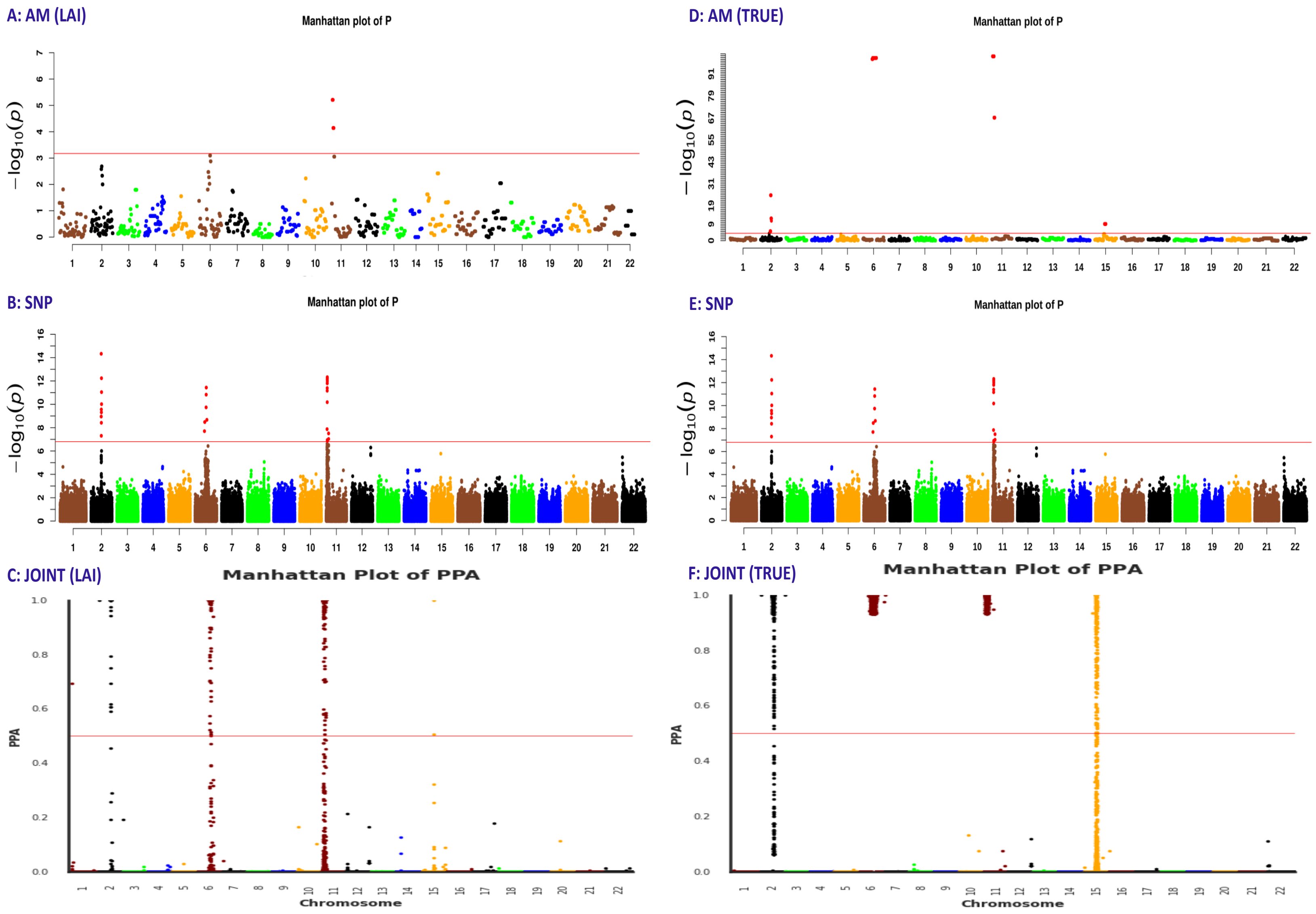

Figure 18: The Manhattan plots A and C correspond to the association analysis for the 3-way admixed population analysis using the RFMIX inferences (LAI), while D and F correspond to the association analysis using the TRUE local ancestry output by FractalSIM. A and D correspond to the p-values of the admixture mapping, B and E to the genotype association, and C and F to the joint PPA. The significance threshold line is shown in red.

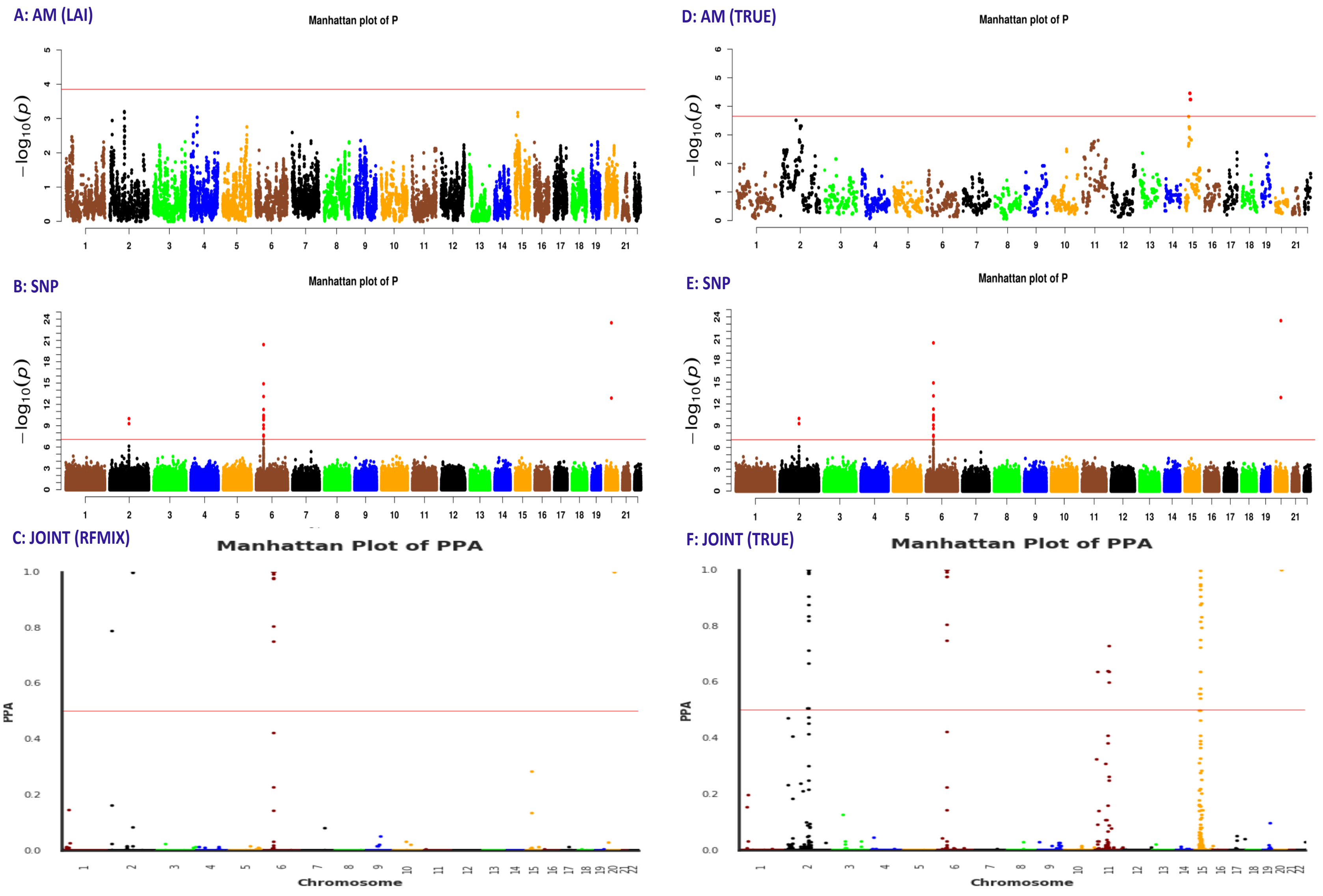

Figure 19: The Manhattan plots A and C correspond to the association analysis using the RFMIX inferences (LAI), while D and F correspond to the association analysis using the TRUE local ancestry output by FractalSIM. A and D correspond to the p-values of the admixture mapping, B and E to the genotype association, and C and F to the joint PPA for the 500 cases and 500 controls in the 5-way admixed population analysis. The significance threshold line is shown in red.

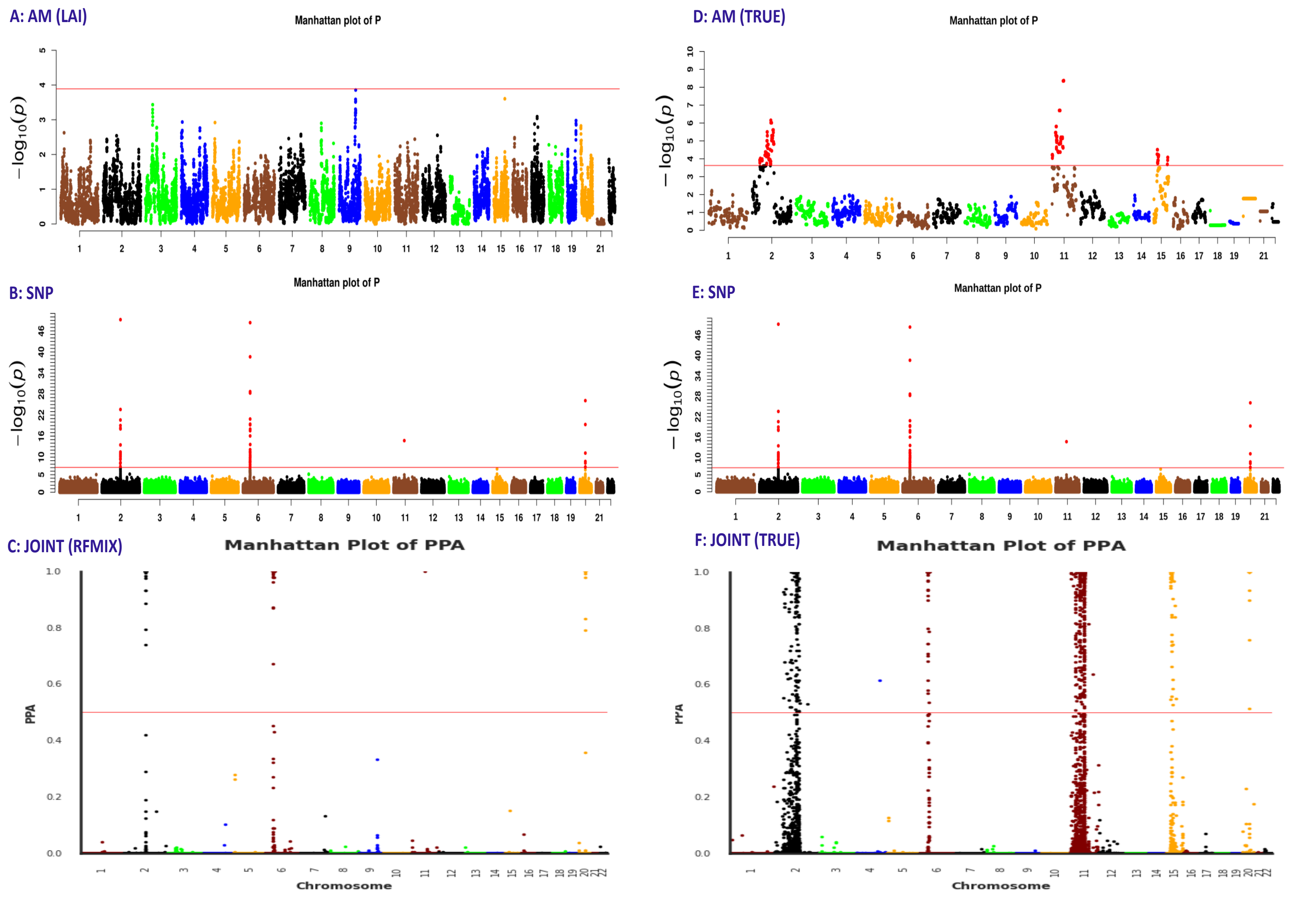

Figure 20: The Manhattan plots A and C correspond to the association analysis using the RFMIX inferences (LAI), while D and F correspond to the association analysis using the TRUE local ancestry output by FractalSIM. A and D correspond to the p-values of the of the admixture mapping, B and E to the genotype association, and C and F to the joint PPA, for the 2500 cases and 2500 controls in the 5-way admixed population analysis. The significance threshold line is shown in red.

Table 40: JasMAP: The list of risk SNPs simulated under the 500 cases 500 controls 5-way admixture scenario, with the correponding most informative ancestry (**MIA**) selected, the odds ratio and p-value under the admixture mapping (**MIA OR** and **MIA Pval**, respectively) the genotype association odds ratio and p-value (**Genotype OR** and **Genotype Pval**, respectively) and the PPA for the joint association (**Joint PPA**) using the RFMIX inferences (**RFMIX**) and the true local ancestry (**TRUE**) output by FractalSIM.

rsID	CHR	Position	RFMIX					
			MIA	MIA OR	MIA Pval	Genotype OR	Genotype Pval	Joint PPA
*rs*13410964	2	113843283	SAS	1.0031	0.35931	0.9559	2.0230 $\times 10^{-05}$	1.3677 $\times 10^{-03}$
*rs*17042838	2	113843337	SAS	1.0031	0.35931	0.9493	1.01154 $\times 10^{-10}$	0.9991
*rs*2232238	6	29942857	EUR	0.9916	0.39185	0.8849	3.86936 $\times 10^{-21}$	0.9999
*rs*7106136	11	64748278	MAFR	0.9995	0.6543	0.9623	7.13465 $\times 10^{-04}$	6.8095 $\times 10^{-07}$
*rs*10897540	11	64757496	MAFR	0.9995	0.6543	0.9620	2.58913 $\times 10^{-04}$	3.606 $\times 10^{-06}$
*rs*11853943	15	48779402	MAFR	0.9726	9.97288 $\times 10^{-03}$	0.9604	1.55297 $\times 10^{-04}$	0.1358
*rs*6115375	20	25871801	MAFR	1.0114	0.20729	0.9176	3.32176 $\times 10^{-24}$	0.9999
*rs*6107104	20	25922993	MAFR	1.0114	0.20729	0.9231	1.29474 $\times 10^{-13}$	0.9999

rsID	CHR	Position	TRUE					
			MIA	MIA OR	MIA Pval	Genotype OR	Genotype Pval	Joint PPA
*rs*13410964	2	113843283	WAFR	0.9699	1.7909 $\times 10^{-03}$	0.9559	2.02304 $\times 10^{-05}$	0.9865
*rs*17042838	2	113843337	WAFR	0.9699	1.7909 $\times 10^{-03}$	0.9493	1.01154 $\times 10^{-10}$	0.9999
*rs*2232238	6	29942857	EAS	0.9917	0.5108	0.8849	3.86936 $\times 10^{-21}$	0.9999
*rs*7106136	11	64748278	MAFR	1.0383	1.73644 $\times 10^{-03}$	0.9623	7.13465 $\times 10^{-04}$	0.2482
*rs*10897540	11	64757496	MAFR	1.0383	1.73644 $\times 10^{-03}$	0.9620	2.58913 $\times 10^{-04}$	0.6361
*rs*11853943	15	48779402	SAS	0.9413	3.50647 $\times 10^{-05}$	0.9604	1.55297 $\times 10^{-04}$	0.9973
*rs*6115375	20	25871801	WAFR	0.9939	0.55617	0.9176	3.32176 $\times 10^{-24}$	0.9999
*rs*6107104	20	25922993	WAFR	0.9939	0.55617	0.9231	1.29474 $\times 10^{-13}$	0.9999

Table 41: The list of risk SNPs simulated under the 2500 cases 2500 controls 5-way admixture scenario, with the correponding most informative ancestry (**MIA**) selected, the odds ratio and p-value under the admixture mapping (**MIA OR** and **MIA Pval**, respectively) the genotype association odds ratio and p-value (**Genotype OR** and **Genotype Pval**, respectively) and the PPA for the joint association (**Joint PPA**) using the RFMIX inferences (**RFMIX**) and the true local ancestry (**TRUE**) output by FractalSIM.

rsID	CHR	Position	RFMIX					
			MIA	MIA OR	MIA Pval	Genotype OR	Genotype Pval	Joint PPA
*rs*13410964	2	113843283	MAFR	0.9935	0.05683	0.9488	2.51965 $\times 10^{-24}$	0.9999
*rs*17042838	2	113843337	MAFR	0.9935	0.05683	0.9464	6.08731 $\times 10^{-50}$	0.9999
*rs*2232238	6	29942857	MAFR	0.9963	0.38748	0.9188	4.29497 $\times 10^{-49}$	0.9999
*rs*7106136	11	64748278	MAFR	1.0001	0.73299	0.9778	1.11863 $\times 10^{-05}$	0.0001
*rs*10897540	11	64757496	MAFR	1.0001	0.73299	0.9632	1.89689 $\times 10^{-15}$	0.9999
*rs*11853943	15	48779402	MAFR	0.9968	0.43494	0.9810	7.35299 $\times 10^{-05}$	9.71651 $\times 10^{-05}$
*rs*6115375	20	25871801	WAFR	1.0030	0.24228	0.9579	7.4893 $\times 10^{-27}$	0.9999
*rs*6107104	20	25922993	WAFR	1.0030	0.24228	0.9554	4.8832 $\times 10^{-20}$	0.9999

rsID	CHR	Position	TRUE					
			MIA	MIA OR	MIA Pval	Genotype OR	Genotype Pval	Joint PPA
*rs*13410964	2	113843283	MAFR	1.0397	9.69955 $\times 10^{-07}$	0.9488	2.51965 $\times 10^{-24}$	0.9999
*rs*17042838	2	113843337	MAFR	1.0397	9.69955 $\times 10^{-07}$	0.9464	6.08731 $\times 10^{-50}$	0.9999
*rs*2232238	6	29942857	MAFR	0.9958	0.15924	0.9188	4.29497 $\times 10^{-49}$	0.9999
*rs*7106136	11	64748278	SAS	0.9653	4.41531 $\times 10^{-09}$	0.9778	1.11863 $\times 10^{-05}$	0.9999
*rs*10897540	11	64757496	SAS	0.9653	4.41531 $\times 10^{-09}$	0.9632	1.89689 $\times 10^{-15}$	0.9999
*rs*11853943	15	48779402	WAFR	1.0251	6.91912 $\times 10^{-05}$	0.9810	7.35299 $\times 10^{-05}$	0.9983
*rs*6115375	20	25871801	EAS	0.9915	0.01685	0.9579	7.4893 $\times 10^{-27}$	0.9999
*rs*6107104	20	25922993	EAS	0.9915	0.01685	0.9554	4.8832 $\times 10^{-20}$	0.9999

Table 42: The table lists all the risk SNPs simulated under the 3-way and 5-way simulations and all the tools that were assessed. ✓ indicates a tool detected the risk SNP at the significant threshold, while ✗ indicates the SNP was not found to be significant. The last row indicates the time in seconds each tool took to run the association analysis for chromosome 1, consisting of 48,422 SNPs in the 500 cases and 500 controls cohort.

**Simulation**	**Chr**	**Risk SNP**	**EMMAX**	**GEMMA**	**GCTA**	**GCTA LOCO**	**PLINK Logistic**	**SNPTEST Frequentist**	**SNPTEST Bayesian**	**TRACTOR RFMIX**	**JasMAP RFMIX**	**JasMAP TRUE**
3-way	2	*rs*76091761	✓	✓	✓	✓	✓	✓	✓	✓	✓	✓
(2500 cases, 2500 controls)	6	*rs*79354975	✓	✓	✓	✓	✓	✓	✓	✓	✓	✓
	11	*rs*734171851	✗	✗	✗	✓	✓	✓	✓	✓	✓	✓
	15	*rs*2960806	✗	✗	✗	✗	✗	✗	✗	✗	✓	✓
5-way	2	*rs*13410964	✗	✗	✗	✗	✗	✗	✗	✗	✗	✓
(500 cases, 500 controls)	2	*rs*17042838	✓	✓	✓	✓	✓	✓	✓	✓	✓	✓
	6	*rs*2232238	✓	✓	✓	✓	✓	✓	✓	✓	✓	✓
	11	*rs*7106136	✗	✗	✗	✗	✗	✗	✗	-	✗	✗
	11	*rs*10897540	✗	✗	✗	✗	✗	✗	✗	✗	✗	✓
	15	*rs*11853943	✗	✗	✗	✗	✗	✗	✗	✗	✗	✓
	20	*rs*6115375	✓	✓	✓	✓	✓	✓	✓	✓	✓	✓
	20	*rs*6107104	✓	✓	✓	✓	✓	✓	✓	✓	✓	✓
5-way	2	*rs*13410964	✓	✓	✓	✓	✓	✓	✓	✓	✓	✓
(2500 cases, 2500 controls)	2	*rs*17042838	✓	✓	✓	✓	✓	✓	✓	✓	✓	✓
	6	*rs*2232238	✓	✓	✓	✓	✓	✓	✓	✓	✓	✓
	11	*rs*7106136	✗	✗	✗	✗	✗	✗	✗	✗	✗	✓
	11	*rs*10897540	✓	✓	✓	✓	✓	✓	✓	✓	✓	✓
	15	*rs*11853943	✗	✗	✗	✗	✗	✗	✗	✗	✗	✓
	20	*rs*6115375	✓	✓	✓	✓	✓	✓	✓	✓	✓	✓
	20	*rs*6107104	✓	✓	✓	✓	✓	✓	✓	✓	✓	✓
Speed in seconds: Chromosome 1: 48,422 SNPs (500 cases and 500 controls)			207	550	18	122	1	142	121	1126	729	729

References

D. H. Alexander, J. Novembre, and K. Lange. Fast model-based estimation of ancestry in unrelated individuals. *Genome Research*, 19(9):1655–64, 2009.

E. G. Atkinson, A. X. Maihofer, M. Kanai, A. R. Martin, K. J. Karczewski, M. L. Santoro, J. C. Ulirsch, Y. Kamatani, Y. Okada, H. K. Finucane, K. C. Koenen, C. M. Nievergelt, M. J. Daly, and B. M. Neale. Tractor uses local ancestry to enable the inclusion of admixed individuals in gwas and to boost power. *Nature genetics*, 53(2):195–204, 2021.

D. Awany, I. Allali, S. Dalvie, S. Hemmings, K. S. Mwaikono, N. E. Thomford, A. Gomez, N. Mulder, and E. R. Chimusa. Host and microbiome genome-wide association studies: Current state and challenges. *Frontiers in Genetics*, 10:637–637, 2019.

J. A. Brody, A. C. Morrison, J. C. Bis, J. R. O'Connell, M. R. Brown, J. E. Huffman, D. C. Ames, A. Carroll, M. P. Conomos, S. Gabriel, R. A. Gibbs, S. M. Gogarten, N. Gupta, C. E. Jaquish, A. D. Johnson, J. P. Lewis, X. Liu, A. K. Manning, G. J. Papanicolaou, A. N. Pitsillides, K. M. Rice, W. Salerno, C. M. Sitlani, N. L. Smith, S. R. Heckbert, C. C. Laurie, B. D. Mitchell, R. S. Vasan, S. S. Rich, J. I. Rotter, J. G. Wilson, E. Boerwinkle, B. M. Psaty, and L. A. Cupples. Analysis commons, a team approach to discovery in a big-data environment for genetic epidemiology. *Nature Genetics*, 49(11):1560–1563, 2017.

R. Buchmann and S. Hazelhurst. Genesis manual, university of the witwatersrand, johannesburg. *http://www.bioinf.wits.ac.za/software/genesis/Genesis.pdf*, 2014.

A. Buniello, J. A. L. MacArthur, M. Cerezo, L. W. Harris, J. Hayhurst, C. Malangone, A. McMahon, J. Morales, E. Mountjoy, E. Sollis, D. Suveges, Vrousgou. O., P. L. Whetzel, R. Amode, J. A. Guillen, Riat H. S., S. J. Trevanion, P. Hall, H. Junkins, P. Flicek, T. Burdett, L. A. Hindorff, F. Cunningham, and Parkinson H. The nhgri-ebi gwas catalog of published genome-wide association studies, targeted arrays and summary statistics 2019. *Nucleic Acids Research*, 47(Database issue): 51005–D1012, 2019.

K. M. Burkart, T. Sofer, S. J. London, A. Manichaikul, F. P. Hartwig, Q. Yan, M. S. Artigas, L. Avila, W. Chen, S. D. Thomas, A. A. Diaz, I. P. Hall, B. L. Horta, R. C. Kaplan, C. C. Laurie, A. M. Menezes, J. V. Morrison, E. C. Oelsner, D. Rastogi, S. S. Rich, M. Soto-Quiros, A. M. Stilp, M. D. Tobin, L. V. Wain, J. C. Celedón, and R. Graham Barr. A genome-wide association study in hispanics/latinos identifies novel signals for lung function the hispanic community health study/study of latinos. *American journal of respiratory and critical care medicine*, 198(2):208–219, 2018.

M. C. Campbell and S. A. Tishkoff. African genetic diversity: Implications for human demographic history, modern human origins, and complex disease mapping. *Annual Review of Genomics and Human Genetics*, 9(1):403–433, 2008.

W. Chen, J. M. Brehm, A. Manichaikul, M. H. Cho, N. Boutaoui, Q. Yan, K. M. Burkart, P. L. Enright, J. I. Rotter, H. Petersen, S. Leng, M. Obeidat, Y. Bosse, C. Brandsma, K. Hao, S. S. Rich, R. Powell, L. Avila, M. Soto-Quiros, E. K. Silverman, Y. Tesfaigzi, R. G. Barr, and J. C. Celedon. A genome-wide association study of chronic obstructive pulmonary disease in hispanics. *Annals of the American Thoracic Society*, 12(2):340–348, 2015.

E. Chimusa, N. Zaitlen, M. Daya, M. Moller, P. van Helden, N. Mulder, A. Price, and E. Hoal. Genome-wide association study of ancestry-specific tb risk in the south african coloured population. *Human Molecular Genetics*, 23(3):796–809, 2014.

E. R. Chimusa, S. Dalvie, C. Dandara, A. Wonkam, and G. K. Mazandu. Post genome-wide association analysis: dissecting computational pathway/network-based approaches. *Briefings in Bioinformatics*, 20(2):690–700, 2019.

Wonil Chung and Fei Zou. Mixed-effects models for gaw18 longitudinal blood pressure data. *BMC proceedings*, 8(Suppl 1):S87–S87, 2014.

M. P. Conomos, C. A. Laurie, A. M. Stilp, S. M. Gogarten, C. P. McHugh, S. C. Nelson, T. Sofer, L. Fernández-Rhodes, A. E. Justice, M. Graff, K. L. Young, A. A. Seyerle, C. L. Avery, K. D. Taylor, J. I. Rotter, G. A. Talavera, M. L. Daviglus, S. Wassertheil-Smoller, N. Schneiderman, G. Heiss, R. C. Kaplan, N. Franceschini, A. P. Reiner, J. R. Shaffer, R. G. Barr, K. F. Kerr, S. R. Browning, B. L. Browning, B. S. Weir, M. L. Avilés-Santa, G. J. Papanicolaou, T. Lumley, A. A. Szpiro, K. E. North, K. Rice, T. A. Thornton, and C. C. Laurie. Genetic diversity and association studies in us hispanic/latino populations: Applications in the hispanic community health study/study of latinos. *American journal of human genetics*, 98(1):165–184, 2016.

M. A. Coram, H. Fang, S. I. Candille, T. L. Assimes, and H. Tang. Leveraging multi-ethnic evidence for risk assessment of quantitative traits in minority populations (vol 101, pg 218, 2017). *American Journal of Human Genetics*, 101(4):638–638, 2017.

M. Daya, N. Rafaels, T. M. Brunetti, S. Chavan, A. M. Levin, A. Shetty, C. R. Gignoux, M. P. Boorgula, G. Wojcik, M. Campbell, C. Vergara, D. G. Torgerson, V. E. Ortega, A. Doumatey, H. R. Johnston, N. Acevedo, M. I. Araujo, P. C. Avila, G. Belbin, E. Bleecker, C. Bustamante, L. Caraballo, A. Cruz, G. M. Dunston, C. Eng, M. U. Faruque, T. S. Ferguson, C. Figueiredo, J. G. Ford, W. Gan, P. Gourraud, N. N. Hansel, R. D. Hernandez, E. F. Herrera-Paz, S. Jiménez, E. E. Kenny, J. Knight-Madden, R. Kumar, L. A. Lange, E. M. Lange, A. Lizee, P. Maul, T. Maul, A. Mayorga, D. Meyers, D. L. Nicolae, T. D. O'Connor, R. R. Oliveira, C. O. Olopade, O. Olopade, Z. S. Qin, C. Rotimi, N. Vince, H. Watson, R. J. Wilks, J. G. Wilson, S. Salzberg, C. Ober, E. G. Burchard, L. K. Williams, T. H. Beaty, M. A. Taub, I. Ruczinski, R. A. Mathias, K. C. Barnes, and CAAPA. Association study in african-admixed populations across the americas recapitulates asthma risk loci in non-african populations. *Nature Communications*, 10(1):880–880, 2019.

L. Duncan, H. Shen, B. Gelaye, J. Meijsen, K. Ressler, M. Feldman, R. Peterson, and B. Domingue.

Analysis of polygenic risk score usage and performance in diverse human populations. *Nature Communications*, 10(1):3328–3328, 2019.

E. Evangelou and J. P. A Ioannidis. Meta-analysis methods for genome-wide association studies and beyond. *Nature Reviews Genetics*, 14(6):379–389, 2013.

E. Geza, J. W. Mugo, N. J. Mulder, A. Wonkam, E. R. Chimusa, and G. K. Mazandu. A comprehensive survey of models for dissecting local ancestry deconvolution in human genome. *Briefings in Bioinformatics*, 20(5):1709–1724, 2019.

E. Geza, N. J. Mulder, E. R. Chimusa, and G. K. Mazandu. FRANC: A unified framework for multi-way local ancestry deconvolution with high density snp data. *Briefings in Bioinformatics*, 21 (5):1837–1845, 2020.

L. H. Goetz, L. Uribe-Bruce, D. Quarless, O. Libiger, and N. J. Schork. Admixture and clinical phenotypic variation. *Human heredity*, 77(1/4):73–86, 2014.

R. Hassan, I. Allali, F. E. Agamah, S. S. M. Elsheikh, N. E. Thomford, C. Dandara, and E. R. Chimusa. Drug response in association with pharmacogenomics and pharmacomicrobiomics: Towards a better personalized medicine. *Briefings in Bioinformatics*, 22(4), 2021.

J. N. Hellwege, J. M. Keaton, A. Giri, X. Gao, D. R. Velez Edwards, and T. L. Edwards. Population stratification in genetic association studies. *Current Protocols in Human Genetics*, 95: 1.22.1–1.22.23, 2017.

Gabriel E. Hoffman. Correcting for population structure and kinship using the linear mixed model: Theory and extensions. 8(10):e75707–e75707, 2013.

H. Jeffreys. Theory of probability. *Oxford University Press*, pages 95–103, 1961.

H. M. Kang, J. H. Sul, S. K. Service, N. A. Zaitlen, S. Kong, N. B. Freimer, C. Sabatti, and E. Eskin. Variance component model to account for sample structures in genome-wide association studies. *Nature Genetics*, 42(4):348–354, 2010.

H. Kim, P. B. Shetty, S. Tsavachidis, J. Dong, C. I. Amos, H. B. El-Serag, and A. P. Thrift. Admixture mapping in African Americans identifies new risk loci for HCV-related cirrhosis. *Clinical Gastroenterology and Hepatology*, 2022.

C. Kizil, S. Sariya, Y. A. Kim, F. Rajabli, E. Martin, D. Reyes-Dumeyer, B. Vardarajan, A. Maldonado, J. L. Haines, R. Mayeux, I. Z. Jimenez-Velazquez, I. Santa-Maria, and G. Tosto. Admixture mapping of alzheimer's disease in caribbean hispanics identifies a new locus on 22q13.1. *Molecular Psychiatry*, 27(6):2813–2820, 2022.

A. Korte and A. Farlow. The advantages and limitations of trait analysis with gwas: A review. *Plant Methods*, 9(1):29–29, 2013.

A. Korte, B. J. Vilhjálmsson, V. Segura, A. Platt, Q. Long, and M. Nordborg. A mixed-model approach for genome-wide association studies of correlated traits in structured populations. *Nature Genetics*, 44(9):1066–1071, 2012. ISSN 1061-4036.

N. Li and M. Stephens. Modeling linkage disequilibrium and identifying recombination hotspots using single-nucleotide polymorphism data. *Genetics*, 165(4):2213–2233, 2003.

P. Loh, G. Tucker, B. K. Bulik-Sullivan, B. J. Vilhjálmsson, H. K. Finucane, R. M. Salem, D. I. Chasman, P. M. Ridker, B. M. Neale, B. Berger, N. Patterson, and A. L. Price. Efficient bayesian mixed-model analysis increases association power in large cohorts. *Nature Genetics*, 47(3):284–290, 2015.

J. Marchini and B. Howie. Genotype imputation for genome-wide association studies. *Nature Reviews Genetics*, 11(7):499–511, 2010.

U. M. Marigorta, J. A. Rodríguez, G. Gibson, and A. Navarro. Replicability and prediction: Lessons and challenges from gwas. *Trends in genetics*, 34(7):504–517, 2018.

D. Marnetto, K. Parna, K. Lall, L. Molinaro, F. Montinaro, T. Haller, M. Metspalu, R. Magi, K. Fischer, and L. Pagani. Ancestry deconvolution and partial polygenic score can improve susceptibility predictions in recently admixed individuals. *Nature Communications*, 11(1):1628–1628, 2020.

A. R. Martin, C. R. Gignoux, R. K. Walters, G. L. Wojcik, B. M. Neale, S. Gravel, M. J. Daly, C. D. Bustamante, and E. E. Kenny. Human demographic history impacts genetic risk prediction across diverse populations. *American Journal of Human Genetics*, 100(4):635–649, 2017.

J. J. Mcgrath, P. B. Mortensen, P. M. Visscher, and N. R. Wray. Where gwas and epidemiology meet: Opportunities for the simultaneous study of genetic and environmental risk factors in schizophrenia. *Schizophrenia Bulletin*, 39(5):955–959, 2013.

J. W. Mugo, E. Geza, J. Defo, S. S. M. Elsheikh, G. K. Mazandu, N. J. Mulder, and E. R. Chimusa. A multi-scenario genome-wide medical population genetics simulation framework. *Bioinformatics*, 33 (19):2995–3002, 2017.

B. Pasaniuc, N. Zaitlen, G. Lettre, G. K. Chen, A. Tandon, W. H. L. Kao, I. Ruczinski, M. Fornage, D. S. Siscovick, X. Zhu, E. Larkin, L. A. Lange, L. A. Cupples, Q. Yang, E. L. Akylbekova, S. K. Musani, J. Divers, J. Mychaleckyj, M. Li, G. J. Papanicolaou, R. C. Millikan, C. B. Ambrosone, E. M. John, L. Bernstein, W. Zheng, J. J. Hu, R. G. Ziegler, S. J. Nyante, E. V. Bandera, S. A. Ingles, M. F. Press, S. J. Chanock, S. L. Deming, J. L. Rodriguez-Gil, C. D. Palmer, S. Buxbaum, L. Ekunwe, J. N. Hirschhorn, B. E. Henderson, S. Myers, C. A. Haiman, D. Reich, N. Patterson, J. G. Wilson, and A. L. Price. Enhanced statistical tests for gwas in admixed populations: Assessment using african americans from care and a breast cancer consortium. *PLOS Genetics*, 7 (4):e1001371–e1001371, 2011.

A. Pasha and V. Scaria. Pharmacogenomics in the era of personal genomics: A quick guide to online resources and tools. *Omics for Personalized Medicine*, pages 187–211, 2013.

S. Petrovski and D. B. Goldstein. Unequal representation of genetic variation across ancestry groups creates healthcare inequality in the application of precision medicine. *Genome Biology*, 17(1):157, 2016.

S. L. Pulit, de With, S. A. J, de Bakker, and P. I. W. Resetting the bar : Statistical significance in whole-genome sequencing-based association studies of global populations. *Genetic epidemiology*, 41 (2):145–151, 2017.

S. Purcell, B. Neale, K. Todd-Brown, L. Thomas, M. A. R. Ferreira, D. Bender, J. Maller, P. Sklar, P. I. W. de Bakker, M. J. Daly, and P. C. Sham. Plink: A tool set for whole-genome association and population-based linkage analyses. *The American Journal of Human Genetics*, 81(3):559–575, 2007.

S. Ripke, B. Neale, P. Holmans, D. Collier, I. Agartz, M. Albus, S. Bacanu, J. Bene, E. Bevilacqua, R. Bruggeman, R. Buckner, R. Chan, W. Cheng, N. Cohen, D. Curtis, D. Dikeos, T. Dinan, J. Eriksson, V. Escott-Price, M. Farrell, L. Franke, E. Gershon, S. Godard, L. de Haan, F. Henskens, P. Hoffmann, A. Hofman, M. Ikeda, M. Keller, Y. Kim, J. Knowles, V. Kucinskas, C. Laurent, S. Limborska, M. Macek, B. Maher, S. Marsal, M. Mattheisen, A. McIntosh, C. Meijer, P. Michie, V. Milanova, O. Mors, R. Murray, L. Olsen, G. Papadimitriou, E. Parkhomenko, M. Pato, T. Paunio, A. Pulver, A. Reichenberg, J. Roffman, A. Sanders, E. Scolnick, E. Sigurdsson, J. Silverman, H. So, C. Spencer, J. Strohmaier, S. Tosato, J. Veijola, D. Walsh, D. Wang, B. Webb, M. Weiser, S. Witt, A. Wolen, B. Wormley, D. Blackwood, A. Børglum, H. Ehrenreich, M. Gill, S. McCarroll, A. McQuillin, J. Moran, R. Ophoff, M. Rietschel, B. Riley, P. Sklar, M. Daly, P. Sullivan, L. Crisponi, J. Figueroa, C. A. Haiman, P. Hall, A. Irwanto, M. Johansson, E. Lund, P. H. Peeters, J. Peto, N. Rahman, R. Travis, R. B. van der Luijt, Q. Waisfisz, C. Pato, E. Stahl, G. Belbin, M. H. Schierup, S. Purcell, and D. Chasman. Modeling linkage disequilibrium increases accuracy of polygenic risk scores. *American Journal of Human Genetics*, 97(4):576–592, 2015.

M. Salter-Townshend and S. Myers. Fine-scale inference of ancestry segments without prior knowledge of admixing groups. *Genetics*, 212(3):869–889, 2019.

M. F. Seldin, B. Pasaniuc, and A. L. Price. New approaches to disease mapping in admixed populations. *Nature Reviews Genetics*, 12(8):523–8, 2011.

D. Shriner. Overview of admixture mapping. *Current Protocols in Human Genetics*, 94:1.23.1–1.23.8, 2017.

D. Shriner, A. Adeyemo, and C. N. Rotimi. Joint ancestry and association testing in admixed individuals. *PLOS Computational Biology*, 7(12):e1002325–e1002325, 2011.

G. Sirugo, S. M. Williams, and S. A. Tishkoff. The missing diversity in human genetic studies. *CELL*, 177(4):1080, 2019.

B. R. Swenson, T. Louie, H. J. Lin, R. Méndez-Giráldez, J. E. Below, C. C. Laurie, K. F. Kerr, H. Highland, T. A. Thornton, K. K. Ryckman, C. Kooperberg, E. Z. Soliman, A. A. Seyerle, X. Guo, K. D. Taylor, J. Yao, S. R. Heckbert, D. Darbar, L. E. Petty, B. McKnight, S. Cheng, N. A. Bello, E. A. Whitsel, C. L. Hanis, M. A. Nalls, D. S. Evans, J. I. Rotter, T. Sofer, C. L. Avery, and N. Sotoodehnia. Gwas of qrs duration identifies new loci specific to hispanic/latino populations. *PLOS ONE*, 14(6):e0217796–e0217796, 2018.

H. Tang, D. O. Siegmund, N. Johnson, I. Romieu, and S. J. London. Joint testing of genotype and ancestry association in admixed families. *Genetic Epidemiology*, 34(8):783–791, 2010.

T. A. Thornton and J. L. Bermejo. Local and global ancestry inference and applications to genetic association analysis for admixed populations. *Genetic Epidemiology*, 38(S1), 2014.

D. G. Torgerson, C. R. Gignoux, J. M. Galanter, K. A. Drake, L. A. Roth, B. S. Celeste, S. Huntsman, R. Torres, P. C. Avila, R. Chapela, J. G. Ford, J. R. Rodríguez-Santana, W. Rodríguez-Cintrón, R. D. Hernandez, and E. G. Burchard. Case-control admixture mapping in Latino populations enriches for known asthma-associated genes. *Journal of Allergy and Clinical Immunology*, 130(1): 76–82.e12, 2012.

P. M. Visscher, N. R. Wray, Q. Zhang, P. Sklar, M. I. McCarthy, M. A. Brown, and J. Yang. 10 years of gwas discovery: Biology, function, and translation. *American journal of Human Genetics*, 101(1): 5–22, 2017.

C. A. Winkler, G. W. Nelson, and M. W. Smith. Admixture mapping comes of age. *Annual review of genomics and human genetics*, 11(1):65–89, 2010.

J. Yang, S. H. Lee, M. E. Goddard, and P. M. Visscher. Gcta: A tool for genome-wide complex trait analysis. *American journal of Human Genetics*, 88(1):76–82, 2011.

N. Zaitlen, B. Pasaniuc, S. Sankararaman, G. Bhatia, J. Zhang, A. Gusev, T. Young, A. Tandon, S. Pollack, B. J. Vilhjálmsson, T. L. Assimes, S. I. Berndt, W. J. Blot, S. Chanock, N. Franceschini, P. G. Goodman, J. He, A. J. Hennis, A. Hsing, S.A. Ingles, W. Isaacs, R.A. Kittles, E. A. Klein, L.A. Lange, B. Nemesure, N. Patterson, D. Reich, B. A. Rybicki, J. L. Stanford, V. L. Stevens, S. S. Strom, E. A. Whitsel, J. S. Witte, J. Xu, C. Haiman, J. G. Wilson, C. Kooperberg, D. Stram, A. P. Reiner, H. Tang, and A. L. Price. Leveraging population admixture to explain missing heritability of complex traits. *Nature Genetics*, 46(12):1356–1362, 2014.

X. Zhou and M. Stephens. Genome-wide efficient mixed-model analysis for association studies. *Nature Genetics*, 44(7):821–824, 2012.
